# Single Isomer *N*-Heterocyclic Cyclodextrin Derivatives as Chiral Selectors in Capillary Electrophoresis

**DOI:** 10.3390/molecules26175271

**Published:** 2021-08-30

**Authors:** Ida Fejős, Eszter Kalydi, Edit Luca Kukk, Mimimorena Seggio, Milo Malanga, Szabolcs Béni

**Affiliations:** 1 Department of Pharmacognosy, Semmelweis University, Üllői út 26, H-1085 Budapest, Hungary; fejos.ida@pharma.semmelweis-univ.hu (I.F.); kalydi.eszter@pharma.semmelweis-univ.hu (E.K.); lucakukk@gmail.com (E.L.K.); 2 ChemPhotoLab, Department of Drug Sciences and Health, University of Catania, Viale Andrea Doria 6, I-95125 Catania, Italy; mimiseggio@gmail.com; 3 CycloLab Cyclodextrin R&D Ltd., Illatos út 7, H-1097 Budapest, Hungary; malanga@cyclolab.hu

**Keywords:** enantioseparation, single isomer cationic cyclodextrin, NMR, amino acid, enantiomer migration order

## Abstract

In order to better understand the chiral recognition mechanisms of positively charged cyclodextrin (CD) derivatives, the synthesis, the p*K_a_* determination by ^1^H nuclear magnetic resonance (NMR)-pH titration and a comparative chiral capillary electrophoretic (CE) study were performed with two series of mono-substituted cationic single isomer CDs. The first series of selectors were mono-(6-*N*-pyrrolidine-6-deoxy)-β-CD (PYR-β-CD), mono-(6-*N*-piperidine-6-deoxy)-β-CD (PIP-β-CD), mono-(6-*N*-morpholine-6-deoxy)-β-CD (MO-β-CD) and mono-(6-*N*-piperazine-6-deoxy)-β-CD (PIPA-β-CD), carrying a pH-adjustable moiety at the narrower rim of the cavity, while the second set represented by their quaternarized, permanently cationic counterparts: mono-(6-*N*-(*N*-methyl-pyrrolidine)-6-deoxy)-β-CD (MePYR-β-CD), mono-(6-*N*-(*N*-methyl-piperidine)-6-deoxy)-β-CD (MePIP-β-CD), mono-(6-*N*-(*N*-methyl-morpholine)-6-deoxy)-β-CD (MeMO-β-CD) and mono-(6-*N*-(4,4-*N,N*-dimethyl-piperazine)-β-CD (diMePIPA-β-CD). Based on pH-dependent and selector concentration-dependent comparative studies of these single isomer *N*-heterocyclic CDs presented herein, it can be concluded that all CDs could successfully be applied as chiral selectors for the enantiodiscrimination of several negatively charged and zwitterionic model racemates. The substituent-dependent enantiomer migration order reversal of dansylated-valine using PIP-β-CD contrary to PYP-β-CD, MO-β-CD and PIPA-β-CD was also studied by ^1^H- and 2D ROESY NMR experiments.

## 1. Introduction

Chiral recognition has undisputed importance in separation sciences due to the conceivable differences in the effect of enantiomers of biologically active molecules. Thus, there is a perpetual need for developing valuable methods for enantioseparation. The numerous advantages of chiral capillary electrophoresis (CE) over the extensively used high-performance liquid chromatography (HPLC) or gas chromatography (GC) make it an excellent analytical tool due to its fast method development and optimization by screening various parameters in a short time [1,2].

Analytical scale chiral separation in CE is commonly achieved by adding chiral selectors—most frequently cyclodextrins (CDs)—to the background electrolyte (BGE). In order to enhance the chiral separation ability of native CDs, a wide variety of CD derivatives are available through synthetic modifications. The majority of commercially available CDs are randomly substituted ones, consisting of a complex mixture of isomers with various degrees and positions of substitution. As high reproducibility is essential in method development, there is an ever-growing tendency to use single isomer derivatives (SIDs) as chiral selectors [3,4], as those also facilitate the understanding of the chiral recognition mechanisms.

With the application of charged CDs, neutral analytes become resolvable even without intrinsic electrophoretic mobility, furthermore, the charged moiety provides an extra site for enantiomer recognition by forming electrostatic interactions with ionic guests [3,5]. 

To date, charged CD selectors are predominantly represented by anionic derivatives [4], as the majority of the analytes possess basic character, and due to the fact that cationic selectors exhibit adsorption properties to the negatively charged wall of the silica capillary. This may lead to decreased resolution and also result in reduced reproducibility. Nevertheless, using positively charged derivatives as chiral selectors has the advantage of shorter analysis time, owing to higher effective mobility towards the cathode [6,7]. 

The charge of the resolving agent (as well as the analyte) is either permanent or pH-dependent. When the selector is a strong acid or base, the method development is simplified as the electrostatic analyte-selector interaction is ensured in the entire pH range, consequently, the selectivity can be determined as the charged resolving agent migration model (CHARM-model) describes [8]. On the contrary, the protonation state of weak electrolytes depends on the BGE pH, which offers the opportunity to fine-tune the analyte-selector intermolecular interactions in order to enhance separation selectivity [3,9,10]. This approach is lying on the theory that by finding the optimal experimental conditions, the difference in the formation degree of diastereomeric complexes can be maximized [11].

In this work, we present the synthesis, characterization and enantiorecognition study of two series of cationic SIDs. The first set of selectors were mono-substituted β-CD derivatives with four different *N*-heterocycles, namely mono-(6-*N*-pyrrolidine-6-deoxy)-β-CD (PYR-β-CD), mono-(6-*N*-piperidine-6-deoxy)-β-CD (PIP-β-CD), mono-(6-*N*-morpholine-6-deoxy)-β-CD (MO-β-CD) and mono-(6-*N*-piperazine-6-deoxy)-β-CD (PIPA-β-CD), representing weak electrolytes bearing the basic moiety at the narrower rim of the cavity. The quaternarization of the first set resulted in permanent cationic derivatives carrying positive charge irrespectively of the pH, namely mono-(6-*N*-(*N*-methyl-pyrrolidine)-6-deoxy)-β-CD (MePYR-β-CD), mono-(6-*N*-(*N*-methyl-piperidine)-6-deoxy)-β-CD (MePIP-β-CD), mono-(6-*N*-(*N*-methyl-morpholine)-6-deoxy)-β-CD (MeMO-β-CD) and mono-(6-*N*-(4,4-*N,N*-dimethyl-piperazine)-β-CD (diMePIPA-β-CD). Following the p*K_a_* determination of the adjustable cationic derivatives by ^1^H NMR-pH titration, their applicability as chiral selectors for the separation of 22 ionic model analytes including dansylated amino acids (Dns-AAs), pesticides and nonsteroidal anti-inflammatory drugs (NSAIDs) at different pH values were investigated. Although the synthesis and some application of the pH-adjustable derivatives have already been described [12,13,14,15,16,17,18], their utilization as chiral selector in CE was only discussed for PIP-β-CD and PYR-β-CD before, for the separation of ofloxacin [13], meptanizol and its intermediates [14] or Dns-AAs and carboxylic acids [16], respectively. A systematic comparison of their chiral resolving ability has not been described before. Furthermore, to the best of our knowledge, this is the first report on the synthesis and characterization of the whole set of the *N*-methylated counterparts of the abovementioned CD-derivatives, only MePYR-β-CD was described as chiral selector before [16]. Besides the pH-dependent enantioselectivity, their concentration-dependent chiral recognition behavior was studied and compared for the first time.

## 2. Results and Discussion

### 2.1. Synthetic Procedures

**Figure 1:**
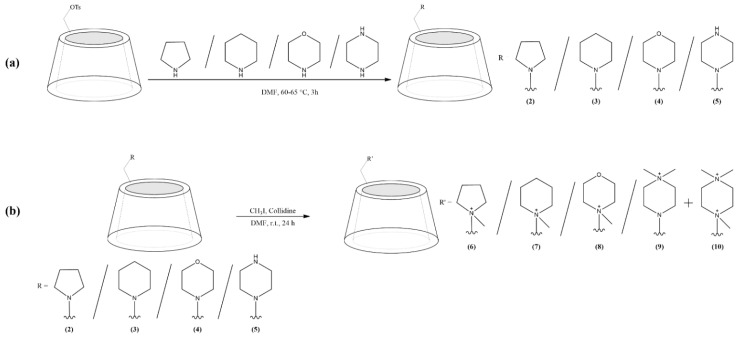
Synthetic schemes and structures of the *N*-heterocycle-substituted β-CD derivatives. (**a**) pH-adjustable derivatives: mono-(6-*N*-pyrrolidine-6-deoxy)-β-CD (PYR-β-CD) (**2**), mono-(6-*N*-piperidine-6-deoxy)-β-CD (PIP-β-CD) (**3**), mono-(6-*N*-morpholine-6-deoxy)-β-CD (MO-β-CD) (**4**), mono-(6-*N*-piperazine-6-deoxy)-β-CD (PIPA-β-CD) (**5**) and (**b**) permanently cationic derivatives: mono-(6-*N*-(*N*-methyl-pyrrolidine)-6-deoxy)-β-CD (MePYR-β-CD) (**6**), mono-(6-*N*-(*N*-methyl-piperidine)-6-deoxy)-β-CD (MePIP-β-CD) (**7**), mono-(6-*N*-(*N*-methyl-morpholine)-6-deoxy)-β-CD (MeMO-β-CD) (**8**), mono-(6-*N*-(4,4-*N,N*-dimethyl-piperazine)-β-CD (diMePIPA-β-CD) (**9**). The compounds 2-5 in (**b**) are the same as that in (**a**).

In this work, two sets of selectors (the pH-adjustable as well as the permanently cationic derivatives) were synthesized according to the schemes shown in Figure 1 (and see Section 3.2). The structures of the final compounds were elucidated by using 1D- and 2D NMR techniques and confirmed by MALDI-TOF-MS (for detailed analytical data see Supplementary Materials Figure S1–S48).

### 2.2. Determination of pK_a_ of the pH-Adjustable CD-Derivatives by ^1^H NMR-Titration

The first set of synthesized CDs bear tertiary amine *N*-heterocycle functions at a single 6-*O* position, thus these selectors exist in two or (in the case of piperazine due to a further secondary amine function) three protonation forms, depending on the pH. The subject of our study was to explore the pH-adjustable chiral selectivity profile of these CD derivatives, thus, to select proper BGE pH values in CE experiments, the acid-base profiling of compounds **2**, **3**, **4** and **5** were conducted. As is shown in Table 1., the experimentally obtained values (see Figure S49–S56 for detailed data) were consistently lower by 1–1.5 p*K_a_* units compared to the predicted ones by Chem Sketch software. 

As the basic moieties can be considered as completely protonated at the pH of p*K_a_*−1 and are found predominantly in the neutral form at the pH of p*K_a_*+1, based on the titration results CE experiments were performed both at pH 10.0, where the selectors are present in non-charged forms and at pH 6.0, where all the studied tertiary amine CDs are positively charged, except for MO-β-CD (which is 42.0% charged contrary to the rest, which is >99.5%). Therefore, pH 4.75 BGE was also used in order to study the fully charged form of MO-β-CD as well.

### 2.3. CE Experiments

**Figure 2:**
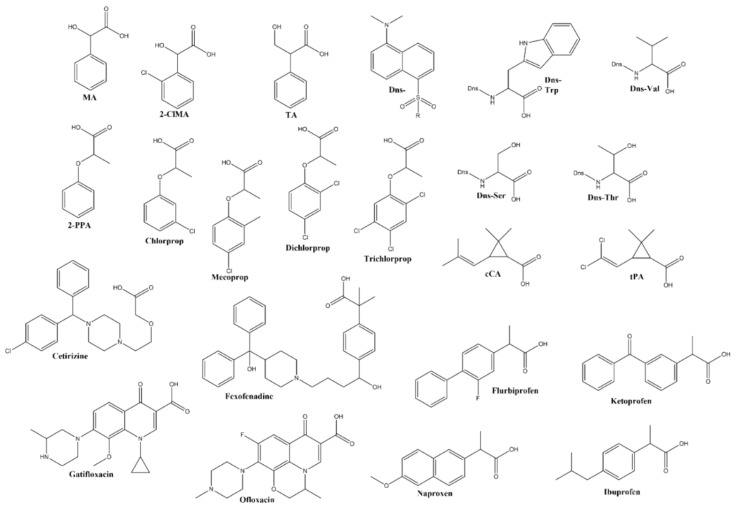
Chemical structures of the test analytes. Organic acids [mandelic acid (MA), 2-chloromandelic acid (2-ClMA), tropic acid (TA), cis-chrysanthemic acid (cCA) and trans-permetrinic acid (tPA)], pesticides [2-phenoxypropionic acid (2-PPA) 2-(3-chlorophenoxy)propionic acid (chlorprop), 2-(2,4-dichlorophenoxy)propanoic acid (dichlorprop), 2-(4-chloro-2-methylphenoxy)propionic acid (mecoprop), 2-(2,4,5-trichlorophenoxy)propionic acid (trichlorprop)], Dns-AAs (Dns-Ser, Dns-Thr, Dns-Trp, Dns-Val), NSAIDs (flurbiprofen, ibuprofen, ketoprofen, naproxen) antihistamines (cetirizine, fexofenadine) and fluoroquinolone antibiotics (gatifloxacin, ofloxacin).

Following the p*K_a_* determination of the mono-substituted CD derivatives, their chiral separation behavior was investigated by CE applying various analytes (Figure 2). Regarding their structure, the model compounds could be categorized into six distinct groups: organic acids [mandelic acid (MA), 2-chloromandelic acid (2-ClMA), tropic acid (TA), cis-chrysanthemic acid (cCA) and trans-permetrinic acid (tPA)], pesticides [2-phenoxypropionic acid (2-PPA) 2-(3-chlorophenoxy)propionic acid (chlorprop), 2-(2,4-dichlorophenoxy)propanoic acid (dichlorprop), 2-(4-chloro-2-methylphenoxy)propionic acid (mecoprop), 2-(2,4,5-trichlorophenoxy)propionic acid (trichlorprop)], Dns-AAs (Dns-Ser, Dns-Thr, Dns-Trp, Dns-Val), NSAIDs (flurbiprofen, ibuprofen, ketoprofen, naproxen) antihistamines (cetirizine, fexofenadine) and fluoroquinolone antibiotics (gatifloxacin, ofloxacin). The chiral recognition ability of the mono-6-*N*-heterocycle-β-CDs and the *N*-methylated mono-6-*N*-heterocycle-β-CDs was thoroughly investigated and summarized in Table 2, Table 3 and Table 4 and Table S1 and furthermore compared with the native- and the amino-β-CDs (Table S2).

#### 2.3.1. Enantioseparation of the Mono-6-*N*-heterocycle-β-CDs (Selectors **2**, **3**, **4**, **5**)

##### Enantioseparation at pH 6.0

Based on the acid-base profiling of the first set of CDs, pH 6.0 phosphate buffer was chosen for the enantioseparation studies, where the tertiary amine CDs are positively charged and most of the analytes possess a negative charge, thus ionic interactions may enhance the diastereomeric complex formation. Cetirizine, fexofenadine, gatifloxacin and ofloxacin have several ionizable functional groups. At pH 6.0 the carboxyl groups are predominantly anionic (the p*K_a_*s of the antibiotics are 5.4–5.7), while the basic groups are predominantly cationic (p*K_a_*s between 6.2–9.0), and this latter may hinder the complex formation. In order to study the selector concentration-dependent chiral discrimination, four CD concentration levels were applied in the range of 0.5–5 mM. The relatively low intrinsic solubility of PIP-β-CD and MO-β-CD hindered the application of 5 mM selector concentration. The obtained resolution values are summarized in Table 2.

In the case of PYR-β-CD, partial or baseline separation could be achieved with 14 model compounds, among which baseline separation could be reached for 9 model racemates (see Table 2). While all the studied racemates of organic acids, pesticides and Dns-AAs, none of the NSAIDs, antihistamines and fluoroquinolone antibiotics could be enantioseparated. Selector concentration-dependent chiral recognition was observed in case of MA, 2-ClMA, TA and Dns-AAs, where the enantioresolution improved by increasing the concentration of the selector (Figure S57A). An opposite trend was observed for cCA and tPA, where the highest resolution values were obtained using 1 mM CD with outstanding enantioselectivity. PYR-β-CD was studied previously by Xiao et al. [9], which correlates with our findings on the CD concentration-dependent enantioselectivity for MA, 2-PPA, chlorprop, dichlorprop, Dns-Ser and Dns-Val as well. 

The PIP-β-CD also showed enantioselectivity towards 14 model compounds, however, only six analytes exhibited baseline separation (see Table 2). Contrary to PYR-β-CD, enantiomeric recognition could be achieved for the enantiomers of ibuprofen and gatifloxacin, but no separation was observed with dichlorprop and Dns-Trp. Remarkable enantioselectivity was obtained for cCA, tPA, chlorprop, trichlorprop, and Dns-Ser using 2.5 mM selector. Ofloxacin could not be enantiorecognized under these experimental conditions, which observation is consistent with a previous study using PIP-β-CD at pH 3.0 [6]. 

With MO-β-CD (being partially protonated at pH 6.0), 12 of the model compounds showed enantiorecognition among which 5 were baseline separated. As the concentration increased, the resolution generally improved, except for trichlorprop and Dns-Thr, where the maximum resolution was reached at 1 mM CD. The cCA, tPA and Dns-Ser enantiomers could be recognized at all CD concentrations.

PIPA-β-CD exhibited partial separation for 11 racemates, among which 3 (cCA, chlorprop and Dns-Thr) were baseline separated (see Table 2). The enantiomers of the zwitterionic fexofenadine could only be resolved by PIPA-β-CD at 0.5 mM concentration, similarly to the gatifloxacin-PIP-β-CD system. Furthermore, the determination of the enantioresolution of the herbicides at 5 mM selector concentration was rather challenging due to the formation of a plateau between the adjacent peaks. This dynamic process needs further investigation.

A general conclusion drawn for the first set of CD derivatives at pH 6.0 is that these selectors showed concentration-dependent enantioselectivity for most of the racemates and PYR-β-CD was found to be the most effective selector followed by the PIP-β-CD.

##### Enantioseparation at pH 4.75

As MO-β-CD possesses a significantly lower p*K_a_* value compared to the studied selectors (p*K_a_* 5.86), the application of a pH 4.75 acetate BGE was necessary to investigate the enantiorecognition of its monocationic form. The enantioselectivity changed dramatically in the case of Dns-AAs and herbicides. Significant improvement in enantioresolutions could be observed in the case of Dns-Val, 2-PPA, chlorprop and trichlorprop (*Rs* 5.31, 5.41, 6.47 and 3.14, respectively), while MO-β-CD lost its selectivity towards the other Dns-AAs and dichlorprop at pH 4.75 (see Table S1). 

##### Enantioseparation at pH 10.0

In order to clarify the role of the protonation state/charge of the selectors on the enantioseparation, a pH 10.0 BGE was chosen, where the CDs are uncharged, and all of the analytes possess a negative charge. Thus, the ionic interactions are negligible and only the hydrophobic interactions and hydrogen-bonding interactions are responsible for complex formation and concomitant enantiorecognition. In this pH-dependent enantioselectivity study, the bottleneck of the experimental work was the solubility of the CDs, as it dropped strikingly, thus a maximum of 0.5 mM selector concentration could only be applied. Contrary to that, Xiao et al. studied the pH-dependent enantioselectivity of PYR-β-CD previously in the pH 6.0–9.0 range at 5 mM selector concentration [9]. 

Comparing the pH 10.0 results to those obtained at pH 6.0 using the same selector concentration, only decreased enantiorecognition was observed, without baseline separation of any racemate (see Table S1). PYR-β-CD and MO-β-CD could partially separate the Dns-Ser enantiomers. PIP-β-CD failed to resolve any of the six racemates separated at pH 6.0, confirming the importance of the ionic interactions. However, an additional test analyte, fexofenadine was recognized at pH 10.0, possibly due to the lack of the repelling positive charges of the analyte and the CD. PIPA-β-CD was found to be the most effective chiral selector at pH 10.0, as four out of the seven racemates separated at pH 6.0 with 0.5 mM selector concentration could be partially resolved moreover, PIPA-β-CD exhibited moderate enantioselectivity toward the two ampholyte antihistamines, cetirizine and fexofenadine. In the light of these results, the ionic interaction is the key factor that governs the enantioresolution, nevertheless, hydrophobic interactions and hydrogen-bonding interactions are also responsible for diastereomeric host–guest complex formation.

#### 2.3.2. Enantioseparation of the *N*-Methylated Mono-6-*N*-heterocycle-β-CDs (Selectors **6**, **7**, **8**, **9**)

##### Enantioseparation at pH 6.0

The chiral selectivity of the permanently charged *N*-methylated analogs was studied at pH 6.0 to get a deeper insight into the effect of the CD sidechain *N*-methylation on the enantioselectivity. Results are summarized in Table 3.

MePYR-β-CD allowed the recognition of 11 test racemates. Compared to the tertiary amine analogue PYR-β-CD, MePYR-β-CD lost its selectivity towards the enantiomers of MA, TA and dichlorprop. Reduced enantioselectivity could be observed for several analytes, similarly to the result of Xiao et al. [9]. The quaternarization of PYR-β-CD led to the separation of ketoprofen enantiomers. 

The MePIP-β-CD was found to be the most versatile selector among the permanently cationic CDs resolving 14 analytes. MePIP-β-CD exhibited similar chiral selectivity as PIP-β-CD, however, no separation was observed for 2-ClMA and gatifloxacin, while dichlorprop and ketoprofen could partially be resolved by this quaternary derivative.

MeMO-β-CD resulted in 13 partial separations, with similar selectivity pattern to MePIP-β-CD. Comparing to MO-β-CD, the *N*-methylated analog MeMO-β-CD could differentiate MA, flurbiprofen and ketoprofen enantiomers. MeMO-β-CD was the only *N*-heterocyclic CD derivative capable to discriminate flurbiprofen enantiomers at pH 6.0 BGE, highlighting the importance of the side chain type in the chiral selectivity profile. 

The diMePIPA-β-CD represented the lowest chiral resolving ability among the studied *N*-heterocyclic CDs at pH 6.0, only seven test analyte enantiomers could be partially separated, nevertheless, mecoprop and Dns-Thr showed baseline separation. Comparing with its tertiary amine analog, the enantioselectivity towards the zwitterionic fexofenadine disappeared by the *N*-methylation, highlighting that not the positive charge but the possible H-bonding is responsible for the enantiospecific interaction. 

During the CE measurements using the second set of CDs, a marked color change was observed in the inlet vial. As a result of the synthetic procedure iodide, the counter ion can undergo redox reactions during the electrophoresis.

##### Enantioseparation at pH 10.0

The quaternary ammonium-type derivatives carrying a positive charge over the entire pH range, therefore at pH 10.0 both selectors and selectands are fully ionized with opposite charges and the higher selector solubility (compared to the ionizable CD derivatives) allows the application of 0.5–5 mM concentrations for a pH-dependent enantioselectivity study (Table 4). The majority of the racemates separated at pH 6.0 could also be resolved at pH 10.0 with the appropriate selectors (as expected due to the unchanged ionization states of the selectors and selectands), however, some unexpected enantioselectivity was observed (as the applied running buffers were different, influencing the overall CE conditions and thereby resulting in altered separation ability of the system). The best results were achieved by MePYR-β-CD enabling the discrimination of the same number of racemates at both pHs, with higher resolutions at pH 10.0 (11 enantiorecognitions, 3 baseline separations). Enhanced enantioresolutions were observed in the case of 2-PPA, Dns-Thr and Dns-Val, where baseline separation could be reached, however with longer analysis times (Figure S57). Minor enantioresolution enhancements could be achieved in the case of cCA, tPA, Dns-Trp and ketoprofen enantiomers, moreover, flurbiprofen enantiomers could also be discriminated. The zwitterionic antibiotics and antihistamines lose their positive charges under basic conditions, thus enabling enhanced ionic interactions.

Utilizing MeMO-β-CD at pH 10.0 for organic acids and for pesticides (2-PPA, chlorprop) resulting in higher resolution values, moreover remarkable gain in chiral separation for the Dns-AAs could also be achieved. Under basic conditions, MeMO-β-CD exhibited additional enantiorecognition towards Dns-Trp.

As opposed to MePYR-β-CD and MeMO-β-CD, MePIP-β-CD markedly underperformed at pH 10.0. Its enantioselectivity dropped and only 8 analytes were enantiorecognized instead of the 14 obtained at pH 6.0.

In the case of diMePIPA-β-CD, the pH change resulted in diverse chiral resolution behavior. Additional enantiorecognition could be observed with 2-ClMA, TA, and 2-PPA, moreover, the selector concentration-dependent chiral discrimination properties altered with tPA and trichlorprop.

Comparing all the results listed above it can be concluded that the *N*-methylation assigns additional enantiorecognition to PYR-β-CD and MO-β-CD towards the Dns-AAs, while the methylation of PIP-β-CD fails to deliver additional benefits when using the same test pool of analytes. Under the light of these results, chiral selectivity could be fine-tuned through the quaternarization of the *N*-heterocycles.

#### 2.3.3. Comparison of the Enantioseparation Performance of the Native and the Acyclic Derivatives

In order to study the effect of the heterocyclic moiety itself on the enantioselectivity, the resolving ability of native β-CD and its acyclic amino-β-CD derivatives, mono-(6-*N*-amino-6-deoxy)-β-CD (A-β-CD), its dimethylated analog mono-(6-*N,N*-dimethylamino)-β-CD (DMA-β-CD) and the permanently cationic mono-(6-*N,N*,*N*-trimethylammonium)-β-CD (TMA-β-CD) were also investigated at pH 6.0 where all derivatives are positively charged. Results are summarized in Table S2.

Applying the native β-CD, only partial separation could be observed for selected herbicides and Dns-AAs, corroborating the contribution of the heterocyclic substitution to the enantiorecognition. Among the acyclic amines, A-β-CD exhibited enantiorecognition with nine racemates, among which three analytes were baseline separated. In the case of Dns-AAs, the repulsion between the amino groups (that of the dansyl and the CD) hinders inclusion complexation [19,20] leading to poor enantioselectivity.

The tertiary amine DMA-β-CD showed enantioselectivity towards seven racemates, among which baseline separation could be achieved only with cCA. Comparing with the *N*-heterocyclic CDs, DMA-β-CD exhibited limited enantiorecognition, while PYR-β-CD, PIP-β-CD, MO-β-CD and PIP-β-CD were capable to separate 14, 14, 12 and 11 racemates, respectively. These results interpret that the *N*-heterocyclic moieties assign additional enantioselectivity.

The quaternary TMA-β-CD was the least effective selector from all the studied CDs under these conditions. It allowed the chiral recognition of three test analytes only, without any baseline separation. As these racemates are easily separable with all of the permanently charged derivatives with even higher resolution, the significance of the *N*-heterocyclic moiety on the chiral resolution is inevitable.

#### 2.3.4. Enantiomer Migration Order (EMO)

**Figure 3:**
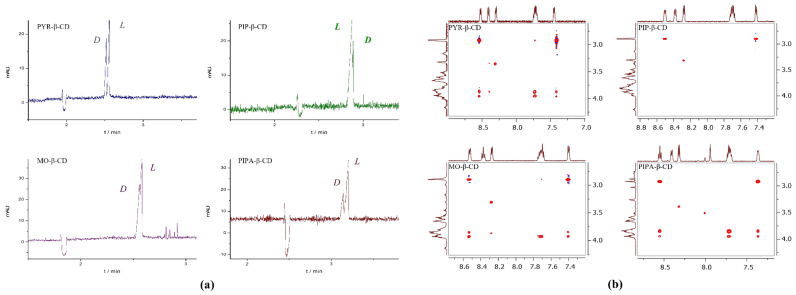
(**a**) Substitution-dependent EMO reversal applying 2.5 mM PYR-β-CD, PIP-β-CD MO-β-CD or PIPA-β-CD at pH 6.0 (48.5/40 cm, 50 µm id capillary, 20 mM NaH_2_PO_4_-NaOH pH 6.0 BGE, 25 kV; 25 °C; 200 nm.) (**b**) Partial 2D ROESY spectra of Dns-Val and 6-*N*-heterocycle-β-CDs showing the section of the feasible interactions between the aromatic region of Dns-Val and the CDs.

The migration order of the enantiomers can hold great significance, as the main component may disturb the chiral analysis due to peak distortion [21,22]. Thus, the determination (and the manipulation) of the EMO could be a crucial point of method development. The EMO was determined by spiking the racemate sample with the individual enantiomers in the case of MA and 2-ClMA using PYR-β-CD resulting in *R, S* migration order at pH 6.0. While *D-, L*- migration order of Dns-Thr, Dns-Trp and Dns-Val was observed for all the studied CDs, PIP-β-CD showed an EMO reversal for Dns-Val at pH 6.0 (Figure 3a). To get a deeper insight into the substituent-dependent enantioselectivity and to clarify the EMO reversal of Dns-Val, complex mobilities and apparent complex stability constants were determined (Supplementary Material, Section 4., Table S3). The obtained results indicate that the enantioselectivity originates from both binding affinity- and intrinsic mobility differences of the diastereomeric complexes [23].

### 2.4. NMR Experiments

The molecular mechanism of EMO reversal was further investigated by ^1^H and 2D ROESY NMR experiments (see Figure S58–S69). Enantioselective interactions were observed for *D,L*-Dns-Val/PYR-β-CD, MO-β-CD and the PIPA-β-CD system, as the aromatic resonances of the dansyl moiety as well as the methyl resonances of the AA moiety showed diastereomeric splitting (see Figures S59, S66 and S68 in SI). Complexation-induced chemical shift changes were also observed in the range of Δδ = 0.03–0.09 ppm. In accordance with the ^1^H NMR data, 2D ROESY spectra confirmed an inclusion-type complexation of the dansyl moiety indicated by intermolecular cross-peaks between the inner protons (H-3 and H-5) of the hosts. Due to similar cross-peaks intensities and overlapping of H-3 and H-5 with H-6 resonances, no conclusive information could be derived on the direction of the penetration of Dns-Val. The broad ^1^H resonances of the *N*-heterocycles preclude the investigation of the heterocycle in the enantiorecognition. Additional intermolecular correlations between the methyl resonances of valine and the cavity protons of PYR-β-CD and MO-β-CD indicated the co-existence of a further inclusion geometry of Dns-Val. To clarify the stoichiometry of Dns-Val and MO-β-CD the Job’s method of continuous variation was applied. The results suggested a 1:1 stoichiometry in which both inclusion geometries co-exist (Figures S70–S72).

Contrary to the finding with PYR-, MO- and PIPA-β-CDs, PIP-β-CD failed to discriminate Dns-Val enantiomers, as neither diastereomeric splitting in ^1^H NMR nor intermolecular interactions were detected in the 2D ROESY experiment (see Figure 3b). Consequently, the EMO reversal of Dns-Val in the PIP-β-CD system originated from the lack of dansyl moiety inclusion and a concomitant much weaker interaction with the selector. Most likely this undetected intermolecular interaction is responsible for the successful enantioseparation in CE, however, it could not be evidenced by NMR, highlighting the high sensitivity of CE for the detection of weak enantioselective interactions [24].

## 3. Materials and Methods

### 3.1. Materials

6-Mono-*O*-tosyl-β-CD was the product of CycloLab Ltd. (Budapest, Hungary). Reagents as *N,N*-dimethylformamide (DMF), acetone, 2-propanol, NH_3_ were of reagent grade and sourced from Molar Chemicals Ltd. (Halásztelek, Hungary), piperidine, piperazine, pyrrolidine, morpholine, iodomethane (CH_3_I), collidine were of reagent grade and purchased from Merck KGaA (Darmstadt, Germany), potassium hydroxide, hydrochloric acid, sodium hydroxide, phosphoric acid, acetic acid, Tris, sodium dihydrogen phosphate and sodium tetraborate were of reagent grade and sourced from Sigma-Aldrich (St. Louis, MO, USA). D_2_O (99.9% D atom) and silica gel (0.0063–0.200 mm for column chromatography) were purchased from Merck KGaA (Darmstadt, Germany). The chiral test analytes used in the CE measurements were obtained from Sigma-Aldrich except for mandelic acid, which was obtained from Merck KGaA.

### 3.2. Synthetic Procedures

#### 3.2.1. Synthesis of Mono-(6-*N*-heterocycle-6-deoxy)-β-CDs (Compounds **2**, **3**, **4**, **5**)

General procedure: 6-Mono-*O*-tosyl-β-CD (10 g) was added portion-wise to a stirred solution of DMF (100 mL) containing an excess of the corresponding base (50 mL or 25 g in case of piperazine) at 60–65 °C under inert atmosphere. The reactions mixtures were heated at 60–65 °C for 3 h. The progress of the reactions was monitored by TLC (1,4-dioxane:25% NH_3_(aq) = 10:7). The solvent was removed by rotary evaporator at 50 °C and the orange viscous product was precipitated with 2-propanol (50 mL). The solid was filtered on a glass filter (porosity 3) and washed with 2-propanol (3 × 50 mL) yielding a yellowish powder. To remove the traces of tosylate and the residual base, the material was suspended in water (5 g in 5 mL), dissolved by setting the pH to 4 with 1 M HCl solution, then precipitated with 25% NH_3_ (aq) (10 mL). Finally, to get rid of the residual solvent traces, the product was dialyzed against deionized water for two days. The yields of the final products were 55–63%.

#### 3.2.2. Synthesis of Mono-(6-*N*-(*N*-methylheterocycle)-6-deoxy)-β-CDs (Compounds **6**, **7**, **8**, **9**, **10**)

General procedure: Collidine (1.5 mL) and CH_3_I (1 mL) were added in sequence to a stirred solution of mono-(6-*N*-heterocycle-6-deoxy)-β-CD (3 g) in DMF (30 mL). The reaction mixture was stirred at room temperature overnight. The progress of the reaction was monitored by TLC (1,4-dioxane:25% NH_3_(aq) = 10:7). The solvent was removed by rotary evaporator at 50 °C and the obtained residue was precipitated with acetone (30 mL). The yellowish solid was filtered on a glass filter (porosity 3) and washed with acetone (3 × 30 mL). To remove the UV active impurities, the crude product (2.5 g) was solubilized in DMF (2.5 mL) and precipitated with acetone (10 mL). Finally, to get rid of the residual solvent traces, the product was dialyzed against deionized water for two days. In the case of compound (**5**), column chromatography was used to separate the final compound (**9**) from the over methylated dicationic sideproduct (**10**) The column was prepared using 150 g silica gel (5 × 30 cm) and freshly prepared methanol:ammonium-acetate:acetic acid = 100:1:0.1 was used as eluent (eluent A) to collect compound **9**, then 20 *v/v*% water was added to eluent A to elute compound **10**. The yields of the final products were 40–52%.

### 3.3. NMR Experiments

All NMR experiments were recorded at 298 K on a 600 MHz Varian DDR NMR spectrometer equipped with a 5 mm inverse-detection gradient (IDPFG) probe.

For the pH-dependent series of ^1^H NMR spectra, the solvent mixture was H_2_O:D_2_O = 9:1 by volume, the constant ionic strength and the buffer capacity were adjusted using 0.05 M NaCl and 0.05 M H_3_PO_4_. An appropriate amount of the corresponding CD was weighed to obtain a 1 mM solution and finally, methanol was added (0.05 mM) as a chemical shift reference (3.31 ppm). The pH was adjusted by using 1 M NaOH. The water resonance was diminished by the *dpfgse* pulse sequence [25]. The pH measurements were performed in 25 mL vessels with proper stirring, before transferring 600 μL solutions into the NMR tubes. The pH meter readings were recorded using a Metrohm pH meter, equipped with a Metrohm 6.0234.110 combined glass electrode.

The p*K_a_* values were calculated using the equation (1) for compounds **2**, **3**, **4** and equation (2) for compound **5**, where $\delta_{obs}$ is the observed chemical shift of a given nucleus, appearing at the frequency of the weighted average of $\delta_{L}$, $\delta_{HL}$ and $\delta_{H_{2}L}$, the limiting chemical shifts of the species simultaneously present [26].

(1) $$\delta_{obs}=\frac{\delta_{L}+\delta_{HL}\times K\left[H^{+}\right]}{1+K\left[H^{+}\right]}=\frac{\delta_{L}+\delta_{HL\;}\times 10^{pK_{a}-pH}}{1+10^{pK_{a\;}-pH}}$$

(2) $$\delta_{obs}=\frac{\delta_{L}+\delta_{HL}\times 10^{pK_{a}1-pH}+\delta_{H_{2}L}\times 10^{pK_{a}1-pH}\times 10^{pK_{a}2-pH}}{1+10^{pK_{a}-pH}+10^{pK_{a}1-pH}\times 10^{pK_{a}2-pH}}$$

### 3.4. CE Experiments

All CE experiments were performed on an HP ^3D^CE (Agilent Technologies, Waldbronn, Germany), equipped with a photodiode array detector and the Chemstation software (Openlab CDS Chemstation Edition Rev. C.01.04, Agilent Technologies, Waldbronn, Germany) for data handling. An untreated fused silica capillary (50 µm id, 48.5 cm total 40 cm effective length) was purchased from Agilent. Conditioning of new capillaries was conducted by flushing with 1 M NaOH for 30 min followed by 0.1 M NaOH and water for 60 min each. Prior to all runs, the capillary was preconditioned by flushing with water (1 min), 0.1 M NaOH (3 min), water (2 min) and BGE (3 min). The running buffers were 20 mM NaH_2_PO_4_ adjusted to pH 6.0 by 1 M NaOH, 20 mM acetic acid adjusted to pH 4.75 by 1 M Tris and 20 mM Na_2_B_4_O_7_ adjusted to pH 10.0 by 1 M NaOH. The BGE contained CDs at various concentrations (0.5–5 mM). The temperature of the capillary was set to 25 °C, UV detection was performed at 200 nm and 25 kV voltage was applied. Samples were injected hydrodynamically (40 mbar,·3 s). The stock solutions of the test racemates were prepared in methanol:water 1:1 (1 mg/mL) and appropriate dilutions with water were used to prepare sample solutions. The resolutions were obtained from a single electrophoretic run. In the case of enantioresolution the *R_S_* values were calculated with the Equation (3):

(3) $$R_{S}=\frac{2\left(t_{R}-t_{S}\right)}{w_{R}+w_{S}}$$

where *w_R_* and *w_S_* stand for the extrapolated peak widths at the baseline.

The enantiomer migration order was determined for MA, 2-ClMA, Dns-Thr, Dns-Trp and Dns-Val, by spiking the stock solutions with the individual enantiomers. In the case of EMO determination, all the separations were run in triplicate.

## 4. Conclusions

This paper reports the syntheses, the acid-base profiling and the chiral capillary electrophoretic study of single isomer *N*-heterocyclic CDs, that carries the positively charged functions exclusively on their primary side and are unmodified on the secondary rim. The chiral recognition ability of the *N*-heterocyclic CD derivatives was investigated and compared with their unmodified analog native β-CD and the acyclic, amino-β-CDs using 22 racemic model compounds at various pHs. Among the *N*-heterocyclic CD derivatives, MO-β-CD, PIPA-β, MePIP-β-CD, MeMO-β-CD, diMePIPA- β-CD were investigated in chiral CE for the first time. PYR-β-CD was found to be the most effective selector, followed by the PIP-β-CD at pH 6.0. 

The *N*-methylation of these CDs altered their enantiorecognition properties, and it was found to be advantageous in the case of MePIP-β-CD and MeMO-β-CD. The EMO was also investigated for MA, 2-ClMA, Dns-Thr, Dns-Trp and Dns-Val. For the Dns-Val/PYR-β-CD or MO-β-CD or PIPA-β-CD and the PIP-β-CD system, complex mobilities and apparent complex stability values were determined and ^1^H and 2D ROESY NMR experiments conducted in order to study the substituent-dependent EMO reversal. Our results highlighted the significance of the *N*-heterocyclic substituent moiety on the complex formation, the chiral resolution and the EMO on SIDs.

## Figures and Tables

**Table 1:** Predicted and Measured p*K*_a_ Values of the pH-Adjustable CD Derivatives.

Compounds	Predicted p*K_a_*	Measured p*K_a_*
PYR-β-CD	9.50	8.78 ± 0.01
PIP-β-CD	9.37	8.43 ± 0.01
MO-β-CD	7.15	5.86 ± 0.01
PIPA-β-CD	4.50 9.26	3.05 ± 0.02 9.08 ± 0.01

**Table 2:** Summary of chiral separations of the tested model racemates with the four ionizable *N*-heterocyclic CDs (the first set of CD derivatives) at pH 6.0, Indicating the maximum resolution values (*Rs*).

	PYR-β-CD				PIP-β-CD			MO-β-CD			PIPA-β-CD			
	0.5 mM	1 mM	2.5 mM	5 mM	0.5 mM	1 mM	2.5 mM	0.5 mM	1 mM	2.5 mM	0.5 mM	1 mM	2.5 mM	5 mM
**MA**	-	0.41	1.00	**1.93**	-	-	0.54	-	-	-	-	-	-	0.36
**2-ClMA**	-	0.55	1.22	**2.36**	-	-	0.60	-	-	-	-	-	0.36	0.50
**TA**	-	-	0.49	0.85	-	0.25	0.64	-	-	-	-	-	-	-
**cCA**	**1.80**	**8.57**	**6.26**	**5.37**	0.91	**3.50**	**4.77**	1.40	**2.56**	**3.14**	0.42	1.46	**1.51**	0.98
**tPA**	1.09	**4.47**	**2.63**	**2.45**	0.54	**1.50**	**1.65**	0.64	**1.50**	**1.69**	0.53	0.53	0.67	0.45
**2-PPA**		0.78	**1.96**	**1.89**	-	0.51	0.99	-	0.97	1.28	-	0.43	0.82	plateau
**Chlorprop**	0.49	**1.66**	**3.31**	0.52	0.35	1.33	**2.67**	-	1.06	**1.70**	0.49	0.70	**1.50**	plateau
**Mecoprop**	0.35	0.21	1.34	0.67	0.17	0.62	0.90		1.15	**1.53**	-	0.28	0.47	plateau
**Dichlorprop**	-	0.41	0.67	0.94	-	-	-	-	-	1.01	-	-	-	plateau
**Trichlorprop**	0.63	n.a.	1.05	0.53	-	**1.52**	**1.65**	-	1.09	0.94	1.04	0.64	1.42	plateau
**Dns-Ser**	0.72	**1.68**	**2.57**	**3.14**	0.81	**1.77**	**2.49**	0.87	1.08	**2.30**	0.56	0.82	0.96	1.11
**Dns-Thr**	0.37	1.21	**1.87**	1.16	-	1.12	**1.51**	-	1.19	0.56	0.28	1.05	**1.56**	1.01
**Dns-Trp**	-	-	1.09	1.13	-	-	-	-	-	0.99	-	-	-	-
**Dns-Val**	-	0.67	**2.07**	**2.08**	-	-	1.15	-	0.15	0.65	0.08	0.63	0.79	1.08
**Ibuprofen**	-	-	-	-	0.17	0.20	0.28	-	-	1.02	-	-	-	-
**Fexofenadine**	-	-	-	-	-	-	-	-	-	-	0.77	n.a.	-	-
**Gatifloxacin**	-	-	-	-	0.39	-	-	n.a.	n.a.	n.a.	n.a.	n.a.	n.a.	-

Legend: -: no separation, n.a.: electropherogram could not be evaluated (due to peak distortion caused by the proximity of the EOF). CE conditions: 48.5/40 cm, 50 µm id capillary, 0.5–5 mM CD in 20 mM NaH_2_PO_4_-NaOH pH 6.0 buffer, 25 kV; 25 °C; 200 nm.

**Table 3:** Chiral separations of the tested model racemates with the four permanently positively charged *N*-heterocyclic CD derivatives (second set) at pH 6.0, indicating the maximum resolution values (*Rs*).

	MePYR-β-CD				MePIP-β-CD				MeMO-β-CD				diMePIPA-β-CD			
	0.5 mM	1 mM	2.5 mM	5 mM	0.5 mM	1 mM	2.5 mM	5 mM	0.5 mM	1 mM	2.5 mM	5 mM	0.5 mM	1 mM	2.5 mM	5 mM
**MA**	-	-	-	-	-	-	0.35	0.57	-	0.16	0.17	n.a.	-	-	-	-
**TA**	-	-	-	-	-	-	-	0.62	-	-	-	n.a.	-	-	-	-
**cCA**	-	0.42	0.95	1.32	-	0.56	0.63	0.70	0.32	0.42	n.a.	n.a.	-	n.a.	n.a.	n.a.
**tPA**	0.41	0.50	0.44	0.51	0.32	0.46	0.51	0.48	0.62	n.a.	n.a.	n.a.	0.29	n.a.	-	-
**2-PPA**	-	-	0.49	0.67	-	-	0.41	0.60	-	-	0.34	0.35	-	n.a.	n.a.	n.a.
**Chlorprop**	-	-	0.67	0.54	-	-	-	0.37	-	0.15	0.42	0.56	0.19	n.a.	0.46	-
**Mecoprop**	-	0.38	0.82	1.17	0.36	0.52	0.81	1.10	0.31	0.46	0.62	0.97	0.09	n.a.	0.47	1.57
**Dichlorprop**	-	-	-	-	-	-	0.24	0.14.	-	-	-	-	-	-	-	-
**Trichlorprop**	-	-	0.82	1.11	0.42	0.58	0.78	0.89	0.56	0.68	1.07	0.84	0.37	-	-	-
**Dns-Ser**	0.67	1.22	1.56	1.66	0.74	0.94	1.65	1.77	0.73	0.86	1.13	n.a.	0.41	n.a.	n.a.	n.a.
**Dns-Thr**	-	0.45	0.93	1.27	-	0.46	0.97	1.36	0.22	0.36	0.60	0.83	0.21	n.a.	0.52	1.75
**Dns-Trp**	-	-	-	0.51	-	-	-	-	-	-	-	-	-	-	-	-
**Dns-Val**	0.39	0.55	0.62	0.93	0.32	0.81	0.77	0.92	0.22	0.25	0.56	0.57	0.25	0.41	0.50	1.27
**Flurbiprofen**	-	-	-	-	-	-	-	-	0.29	0.82	n.a.	n.a.	-	-	-	-
**Ibuprofen**	-	-	-	-	0.64	0.64	n.a.	n.a.	-	-	0.85	n.a.	-	-	-	-
**Ketoprofen**	-	-	-	0.24	0.09	0.23	0.30	0.36	0.18	0.22	0.27	n.a.	-	-	-	-

Legend: -: no separation, n.a.: electropherogram could not be evaluated (due to peak distortion caused by the proximity of the EOF). CE conditions: 48.5/40 cm, 50 µm id capillary, 0.5–5 mM CD in 20 mM NaH_2_PO_4_-NaOH pH 6.0 buffer, 25 kV; 25 °C; 200 nm.

**Table 4:** Chiral separations of the tested model racemates with the four permanently positively charged *N*-heterocyclic CD derivatives (second set) at pH 10.0, indicating the maximum resolution values (*Rs*).

	MePYR-β-CD				MePIP-β-CD				MeMO-β-CD				diMePIPA-β-CD			
	0.5 mM	1 mM	2.5 mM	5 mM	0.5 mM	1 mM	2.5 mM	5 mM	0.5 mM	1 mM	2.5 mM	5 mM	0.5 mM	1 mM	2.5 mM	5 mM
**MA**	-	-	-	-	-	-	-	-	-	-	-	0.66	-	-	-	n.a.
**2-ClMA**	-	-	-	-	-	-	-	-	-	-	-	-	0.25	-	-	-
**TA**	-	-	-	-	-	-	-	-	-	-	-	-	-	0.56	n.a.	0.76
**cCA**	-	0.31	0.32	1.41	-	-	0.26	0.30	0.10	0.35	0.50	0.85	-	-	-	n.a.
**tPA**	0.56	0.47	0.52	0.79	n.a.	n.a.	n.a.	0.72	0.43	0.51	0.76	1.34	-	0.44	0.53	0.50
**2-PPA**	-	-	0.58	1.80	-	-	-	-	-	-	0.13	0.54	-	0.45	n.a.	n.a.
**Chlorprop**	-	-	0.07	n.a.	-	-	-	-	-	n.a.	0.47	0.63	-	0.31	0.76	n.a.
**Mecoprop**	-	0.32	0.59	1.11	-	-	0.35	0.44	0.10	0.41	0.54	0.84	-	0.41	0.42	n.a.
**Trichlorprop**	0.31	0.53	0.72	1.06	-	0.43	0.50	0.55	0.26	0.43	0.66	0.70	-	0.21	0.57	n.a.
**Dns-Ser**	0.33	0.60	0.57	n.a.	-	-	0.85	1.50	0.39	0.56	1.16	2.64	-	0.55	n.a.	n.a.
**Dns-Thr**	-	0.36	0.57	1.51	-	-	0.45	0.54	-	0.17	0.34	1.75	-	0.44	2.07	1.69
**Dns-Trp**	-	-	0.34	0.59	-	-	-	-	-	-	-	0.24	-	-	-	n.a.
**Dns-Val**	-	0.28	0.57	1.63	-	-	0.19	0.32	-	-	0.48	1.22	-	0.17	0.70	n.a.
**Flurbiprofen**	0.11	0.19	0.08	0.17	n.a.	n.a.	n.a.	n.a.	0.17	0.2	0.68	n.a.	-	-	n.a.	n.a.
**Ketoprofen**	-	-	0.28	0.30	-	-	0.17	0.20	-	0.15	0.13	0.35	-	-	0.19	n.a.

Legend: -: no separation, n.a.: electropherogram could not be evaluated (due to peak distortion caused by the proximity of the EOF). CE conditions: 48.5/40 cm, 50 µm id capillary, 0.5–5 mM CD in 20 mM Na_2_B_4_O_7_-NaOH pH 10.0 buffer, 25 kV; 25 °C; 200 nm.

## Data Availability

Not applicable.

## Supplementary Materials

The following are available online, Figure S1: 1H NMR spectrum of PYR-β-CD with integration, Figure S2. DEPT-edited HSQC spectrum of PYR-β-CD with assignment, Figure S3. 2D COSY spectrum of PYR-β-CD, Figure S4. 2D TOCSY spectrum of PYR-β-CD, Figure S5. HMBC spectrum of PYR-β-CD, Figure S6. MALDI-TOF-MS spectrum of PYR-β-CD, Figure S7. 1H NMR spectrum of PIP-β-CD with integration, Figure S8. DEPT-edited HSQC spectrum of PIP-β-CD with assignment, Figure S9. 2D COSY spectrum of PIP-β-CD, Figure S10. 2D TOCSY spectrum of PIP-β-CD, Figure S11. HMBC spectrum of PIP-β-CD, Figure S12. MALDI-TOF-MS spectrum of PIP-β-CD, Figure S13. 1H NMR spectrum of MO-β-CD with integration, Figure S14. DEPT-edited HSQC spectrum of MO-β-CD with assignment, Figure S15. 2D COSY spectrum of MO-β-CD, Figure S16. 2D TOCSY spectrum of MO-β-CD, Figure S17. HMBC spectrum of MO-β-CD, Figure S18. MALDI-TOF-MS spectrum of MO-β-CD, Figure S19. 1H NMR spectrum of PIPA-β-CD with integration, Figure S20. DEPT-edited HSQC spectrum of PIPA-β-CD with, Figure S21. 2D COSY spectrum of PIPA-β-CD, Figure S22. 2D TOCSY spectrum of PIPA-β-CD, Figure S23. HMBC spectrum of PIPA-β-CD, Figure S24. MALDI-TOF-MS spectrum of PIPA-β-CD, Figure S25. 1H NMR spectrum of MePYR-β-CD with integration, Figure S26. DEPT-edited HSQC spectrum of Me-PYR-β-CD with assignment, Figure S27. 2D COSY spectrum of Me-PYR-β-CD, Figure S28. 2D TOCSY HSQC spectrum of Me-PYR-β-CD, Figure S29. HMBC spectrum of Me-PYR-β-CD, Figure S30. MALDI-TOF-MS spectrum of Me-PYR-β-CD, Figure S31. 1H NMR spectrum of MePIP-β-CD with integration, Figure S32. DEPT-edited HSQC spectrum of MePIP-β-CD with assignment, Figure S33. 2D COSY spectrum of MePIP-β-CD, Figure S34. 2D TOCSY spectrum of MePIP-β-CD, Figure S35. HMBC spectrum of MePIP-β-CD, Figure S36. MALDI-TOF-MS spectrum of MePIP-β-CD, Figure S37. 1H NMR spectrum of MeMO-β-CD with integration, Figure S38. DEPT-edited HSQC spectrum of Me-MO-β-CD with assignment, Figure S39. 2D COSY spectrum of Me-MO-β-CD, Figure S40. 2D TOCSY spectrum of Me-MO-β-CD, Figure S41. HMBC spectrum of Me-MO-β-CD, Figure S42. MALDI-TOF-MS spectrum of MeMO-β-CD, Figure S43. 1H NMR spectrum of MePIPA-β-CD with integration, Figure S44. DEPT-edited HSQC spectrum of Me-PIPA-β-CD with assignment, Figure S45. 2D COSY spectrum of Me-PIPA-β-CD, Figure S46. 2D TOCSY spectrum of Me-PIPA-β-CD, Figure S47. HMBC spectrum of Me-PIPA-β-CD, Figure S48. MALDI-TOF-MS spectrum of Me-PIPA-β-CD, Figure S49. 1H NMR-titration of PYR-β-CD for the determination of the pKa., Figure S50. 1H chemical shift of Hβ of PYR-β-CD plotted as a function of pH for the determination of the pKa, Figure S51. 1H NMR-titration of PIP-β-CD for the determination of the pKa, Figure S52. 1H chemical shift of H5’ of PIP-β-CD plotted as a function of pH for the determination of the pKa, Figure S53. 1H NMR-titration of MO-β-CD for the determination of the pKa., Figure S54. 1H chemical shift of H5’ of MO-β-CD plotted as a function of pH for the determination of the pKa, Figure S55. 1H NMR-titration of PIPA-β-CD for the determination of the pKa, Figure S56. 1H chemical shift of H5’ of PIPA-β-CD plotted as a function of pH for the determination of the pKa, Table S1. Enantioseparation of the tested model racemates with ionizable N-heterocyclic CDs (the first set of CD derivatives) at pH 10.0 and at pH 4.75, indicating the maximum resolution values (Rs), Table S2. Enantioseparation of the tested model racemates with native β-CD, mono-6-amino-β-CD (A-β-CD), mono-6-N,N-dimethyl-amino-β-CD (DMA-β-CD), and mono-6-N,N,N-trimethyl-amino-β-CD (TMA-β-CD), at pH 6.0, indicating the maximum resolution values (Rs), Figure S57. Representative electropherograms of the enantioseparation of Dns-Val demonstrating the effect of the pH on the enantioresolution with 5 mM MePYR-β-CD, Table S3. The enantiomer migration order, the complex mobilities and the apparent complex stability constants of the first and second migrating Dns-Val enantiomers at pH 6.0 phosphate buffer, Figure S58. Stacked 1H NMR spectra of D,L-Dns-Val/PYR-β-CD system in 20 mM NaH2PO4 buffer at pH* 6.0 with increasing PYR-β-CD concentration, Figure S59. Stacked partial 1H NMR spectra of D,L-Dns-Val/PYR-β-CD system in 20 mM NaH2PO4 buffer at pH* 6.0 with increasing PYR-β-CD concentration, Figure S60. 2D ROESY spectrum of D,L-Dns-Val/PYR-β-CD system with intermolecular correlations emphasized and structure of the compounds, Figure S61. Stacked 1H NMR spectra of D,L-Dns-Val/PIP-β-CD system in 20 mM NaH2PO4 buffer at pH* 6.0 with increasing PIP-β-CD concentration, Figure S62. Stacked partial 1H NMR spectra of D,L-Dns-Val/PIP-β-CD system in 20 mM NaH2PO4 buffer at pH* 6.0 with increasing PIP-β-CD concentration, Figure S63. 2D ROESY spectrum of D,L-Dns-Val/PIP-β-CD, Figure S64. Stacked 1H NMR spectra of D,L-Dns-Val/MO-β-CD system in 20 mM NaH2PO4 buffer at pH* 6.0 with increasing MO-β-CD concentration, Figure S65. Stacked partial 1H NMR spectra of D,L-Dns-Val/MO-β-CD system in 20 mM NaH2PO4 buffer at pH* 6.0 with increasing MO-β-CD concentration, Figure S66. 2D ROESY spectrum of D,L-Dns-Val/MO-β-CD system, with intermolecular correlations emphasized and structure of the compounds, Figure S67. Stacked 1H NMR spectra of D,L-Dns-Val/PIPA-β-CD system in 20 mM NaH2PO4 buffer at pH* 6.0 with increasing PIPA-β-CD concentration, Figure S68. Stacked partial 1H NMR spectra of D,L-Dns-Val/PIPA-β-CD system in 20 mM NaH2PO4 buffer at pH* 6.0 with increasing PIPA-β-CD concentration, Figure S69. 2D ROESY spectrum of D,L-Dns-Val/PIPA-β-CD system, with intermolecular correlations emphasized and structure of the compounds, Figure S70. Stacked series of 1H NMR spectra of Dns-Val/MO-β-CD system with increasing molar fraction of MO-β-CD from top to bottom, Figure S71. Chemical shift changes of the aromatic protons of Dns-Val upon changing the mole fraction of Dns-Val in the presence of MO-β-CD, Figure S72. Job’s plot derived from the chemical shift changes of H6 proton of Dns-Val in presence of MO-β-CD.

## Sample Availability

Samples of the compounds (**2–9**) are available from the authors.
