# Supplementary material for: Single Isomer N-Heterocyclic Cyclodextrin Derivatives as Chiral Selectors in Capillary Electrophoresis
Source: Molecules. 2021 Aug 30;26(17):5271. doi: 10.3390/molecules26175271 (PMC8434369; doi:10.3390/molecules26175271)
Supplement: Supplementary file 1 [file molecules-26-05271-s001.zip › molecules-1352073-supplementary.pdf]

Supplementary Materials: Single Isomer *N*-Heterocyclic Cyclodextrin Derivatives as Chiral Selectors in Capillary Electrophoresis

Ida Fejős[1], Eszter Kalydi[1], Luca Edit Kukk[1], Mimimorena Seggio[2], Milo Malanga[3] and Szabolcs Béni[1*]

Description: The supplementary information contains the analytical results to follow the preparation of single isomer *N*-heterocyclic cyclodextrins: the NMR spectra and MS spectra used for the structure elucidation of mono-(6-*N*-pyrrolidine-6-deoxy)-β-CD (PYR-β-CD), mono-(6-*N*-piperidine-6-deoxy)-β-CD (PIP-β-CD), mono-(6-*N*-morpholine-6-deoxy)-β-CD (MO-β-CD), mono-(6-*N*-piperazine-6-deoxy)-β-CD (PIPA-β-CD), mono-(6-*N*-(*N*-methyl-pyrrolidine)-6-deoxy)-β-CD (MePYR-β-CD), mono-(6-*N*-(*N*-methyl-piperidine)-6-deoxy)-β-CD (MePIP-β-CD), mono-(6-*N*-(*N*-methyl-morpholine)-6-deoxy)-β-CD (MeMO-β-CD) and mono-(6-*N*-(4,4-*N*,*N*-dimethyl-piperazine)-β-CD (diMePIPA-β-CD). It contains the determination of pK_a of the pH-adjustable CD-derivatives by ^{1}H NMR-titration and its evaluation by Microcal Origin.

The supplementary information summarizes the enantioseparations with β-CD, mono-6-amino-β-CD (A-β-CD), mono-6-*N*,*N*-dimethyl-amino-β-CD (DMA-β-CD) and mono-6-*N*,*N*,*N*-trimethyl-amino-β-CD (TMA-β-CD), the complex mobilities and the apparent complex stability constants of Dns-Val enantiomers with the *N*-heterocyclic cyclodextrins. The supplementary information demonstrates the host-guest complex formation of Dns-Val by NMR.

1. Characterization of the selectors

Mono-(6-*N*-pyrrolidine-6-deoxy)-β-CD (PYR-β-CD) (2)

^{1}H NMR (600 MHz, D_2O): δ (ppm) 5.19-5.01 (m, 7H, H1), 4.23 (td 3J = 9.8 Hz 3J = 2.6 Hz, 1H, H-3), 4.08-3.44 (m, 44H, H-2, H-3, H-4, H-5, H-6, H-6', H-α,eq), 3.23-3.07 (m, 2H, H-α,ax), 2.24-1.96 (m, 4H, H-β).

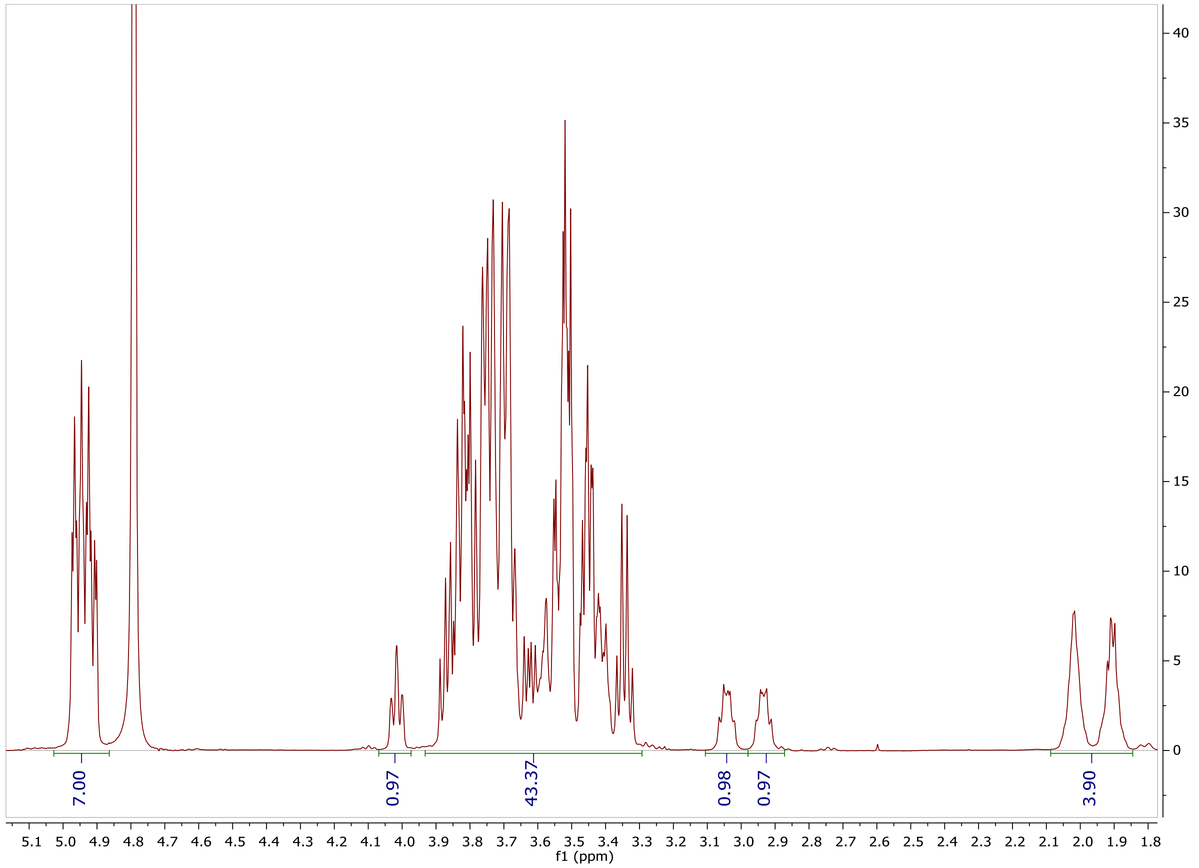

Figure S1. ^{1}H NMR spectrum of PYR-β-CD with integration (600 MHz, 298 K, D_2O).

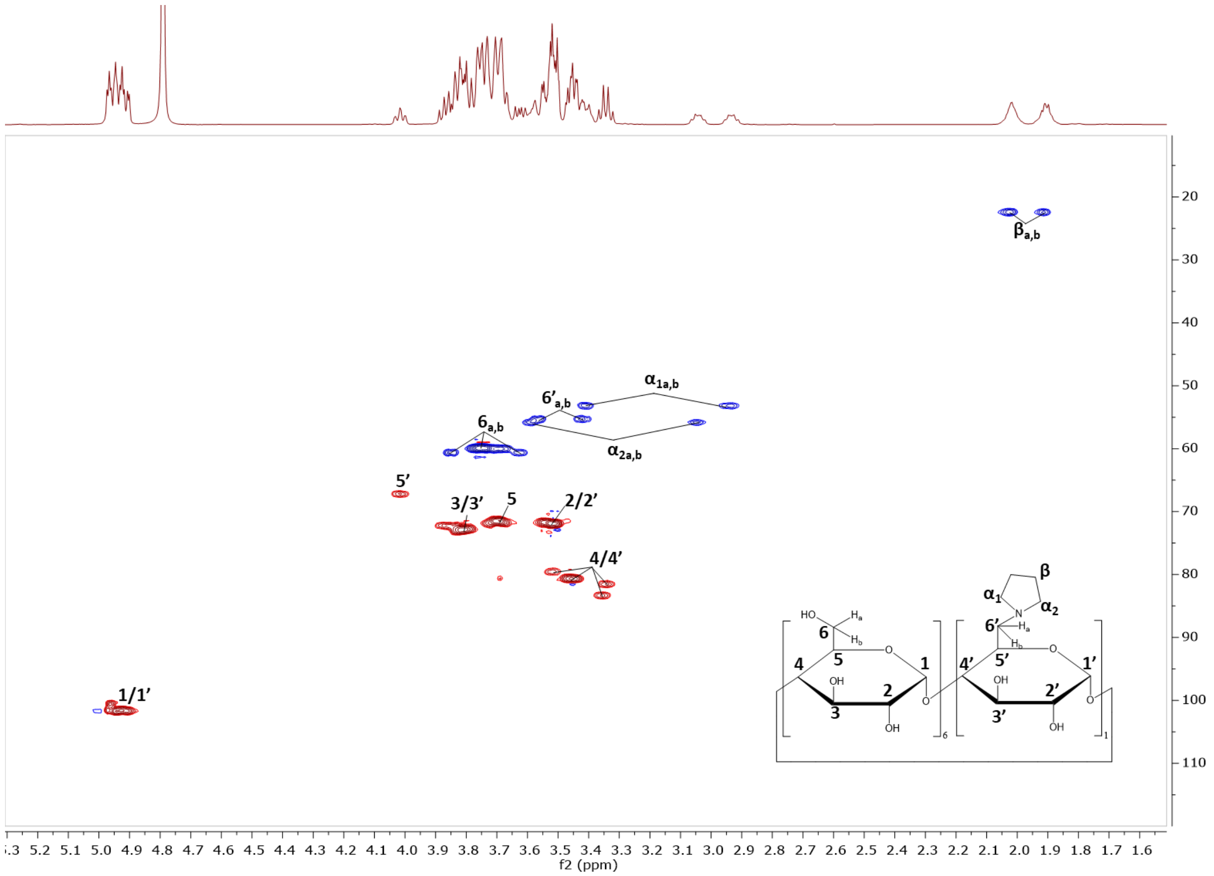

Figure S2. DEPT-edited HSQC spectrum of PYR-β-CD with assignment (600 MHz, 298 K, D_2O).

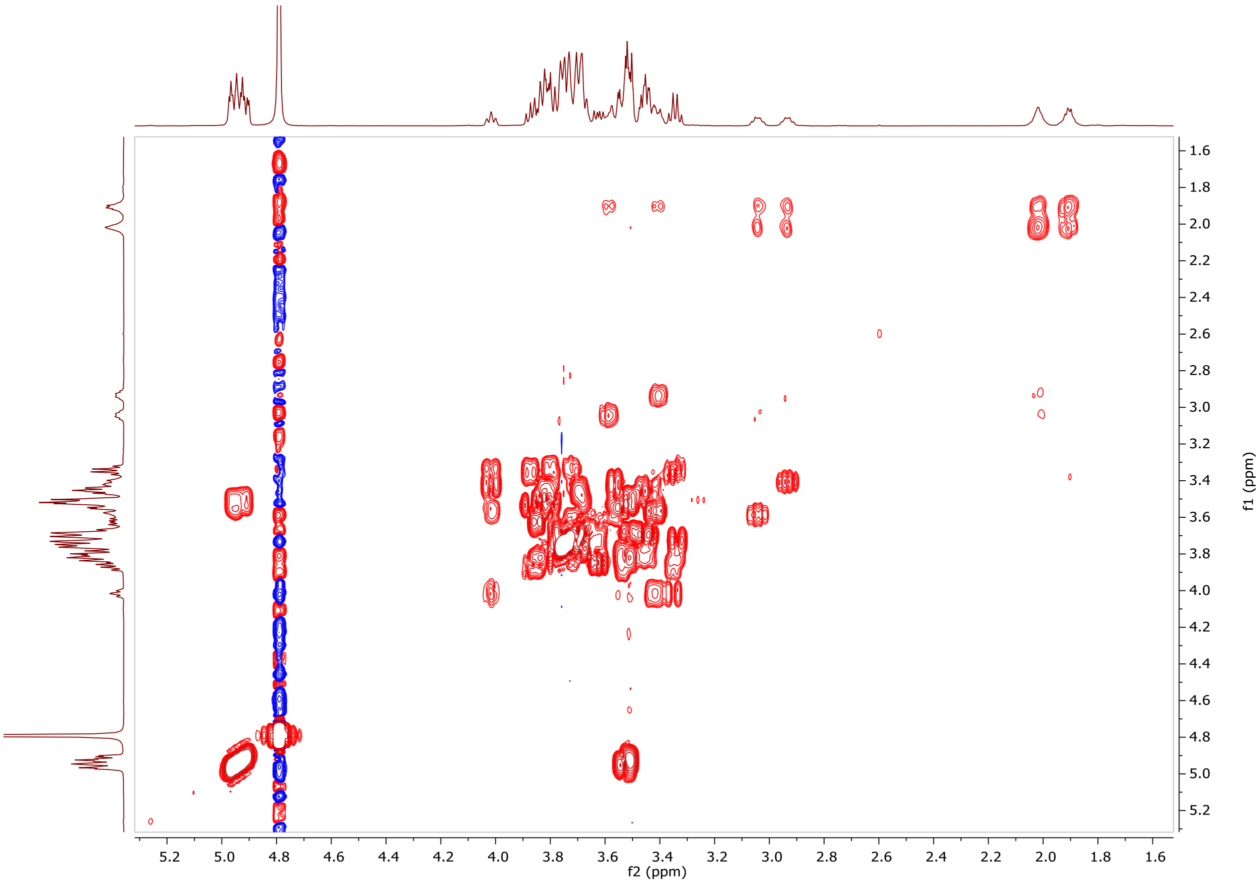

Figure S3. 2D COSY spectrum of PYR-β-CD (600 MHz, 298 K, D_2O).

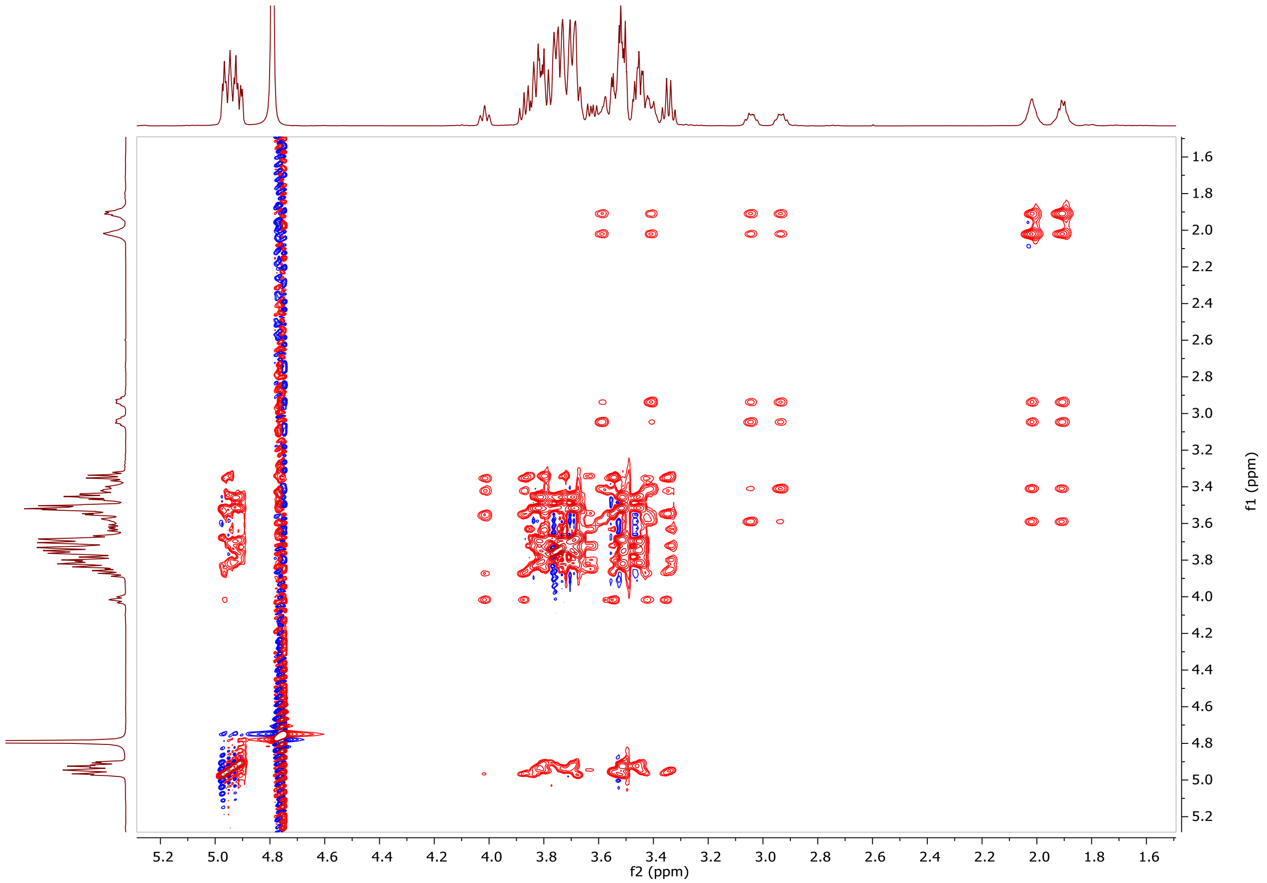

Figure S4. 2D TOCSY spectrum of PYR-β-CD (600 MHz, 298 K, D_2O).

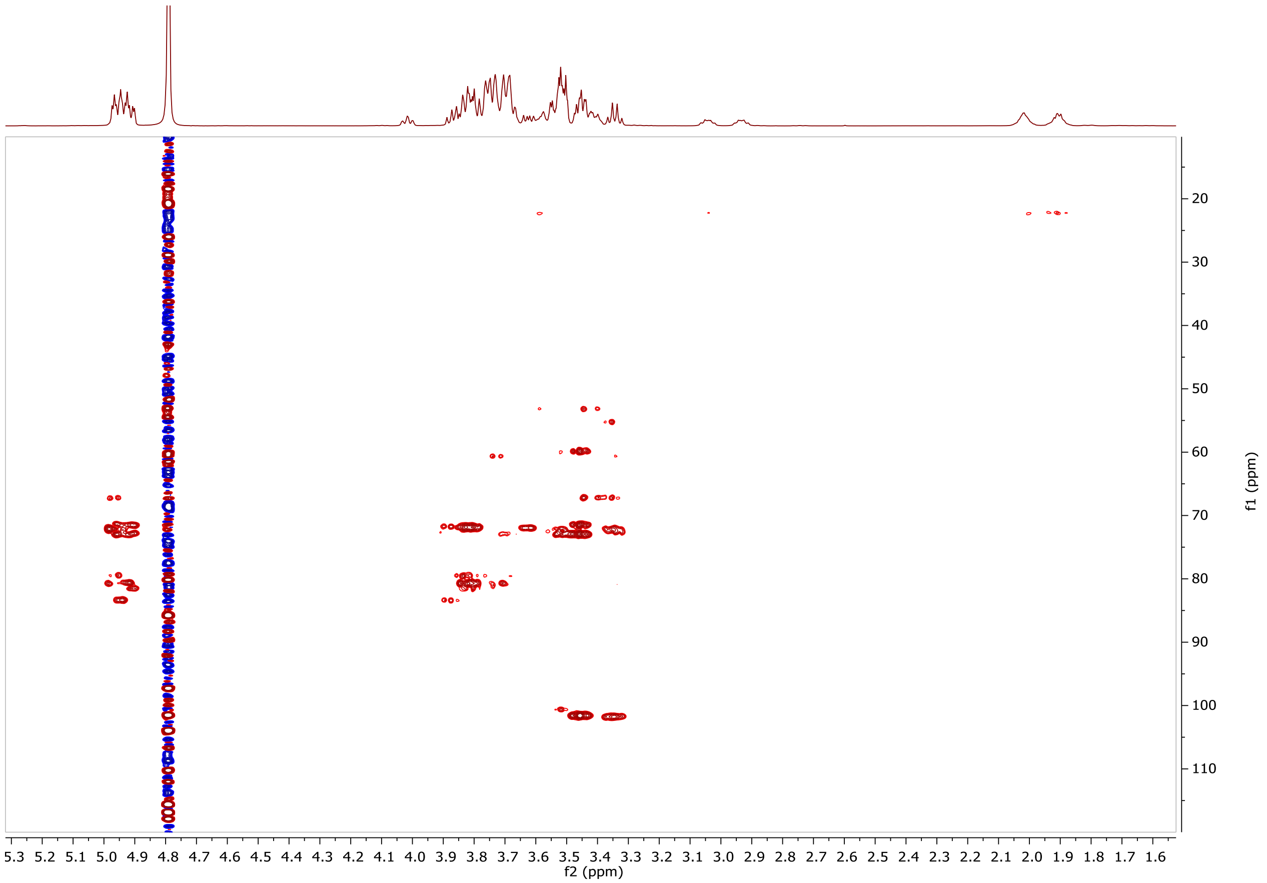

Figure S5. HMBC spectrum of PYR-β-CD (600 MHz, 298 K, D_2O).

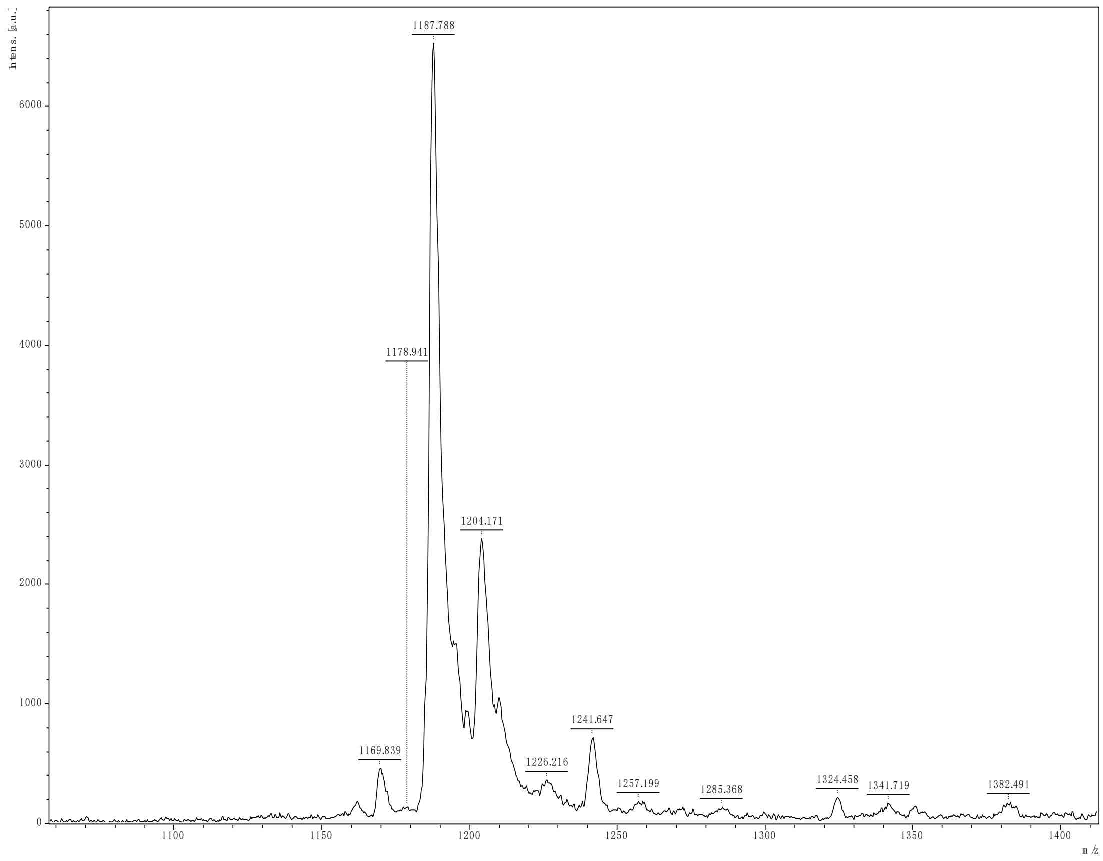

Figure S6. MALDI-TOF-MS spectrum of PYR-β-CD.

Mono-(6-*N*-piperidine-6-deoxy)-β-CD (PIP-β-CD) (3)

^{1}H NMR (600 MHz, D_2O): δ (ppm) 5.00-4.87 (m, 8H, H-1), 4.17 (t, J = 10 Hz, 1H, H-3), 4.03-3.29 (m, 43H, H-1, H-2, H-3, H-4, H-5, H-6a,b, Hα,eq, Hβ), 3.29-3.16 (dd, 3J= 10.5 Hz, 2J = 13.6 Hz, 1H, H-6'a), 2.87 (td, 3J = 12.6 Hz, 2J= 12.6 Hz, 3J = 3.2 Hz, 1H, H-α, ax), 2.78 (td, 3J = 12.6 Hz, 3J = 2.9 Hz, 2J = 12.6 Hz), 1H, H-α, ax), 1.80 (t, 3J = 14.3 Hz, 2J = 14.3 Hz, 2H, H-β,eq), 1.74-1.50 (m, 3H, H-β,ax, H-γ,eq), 1.39-1.27 (m, 1H, H-γ,ax).

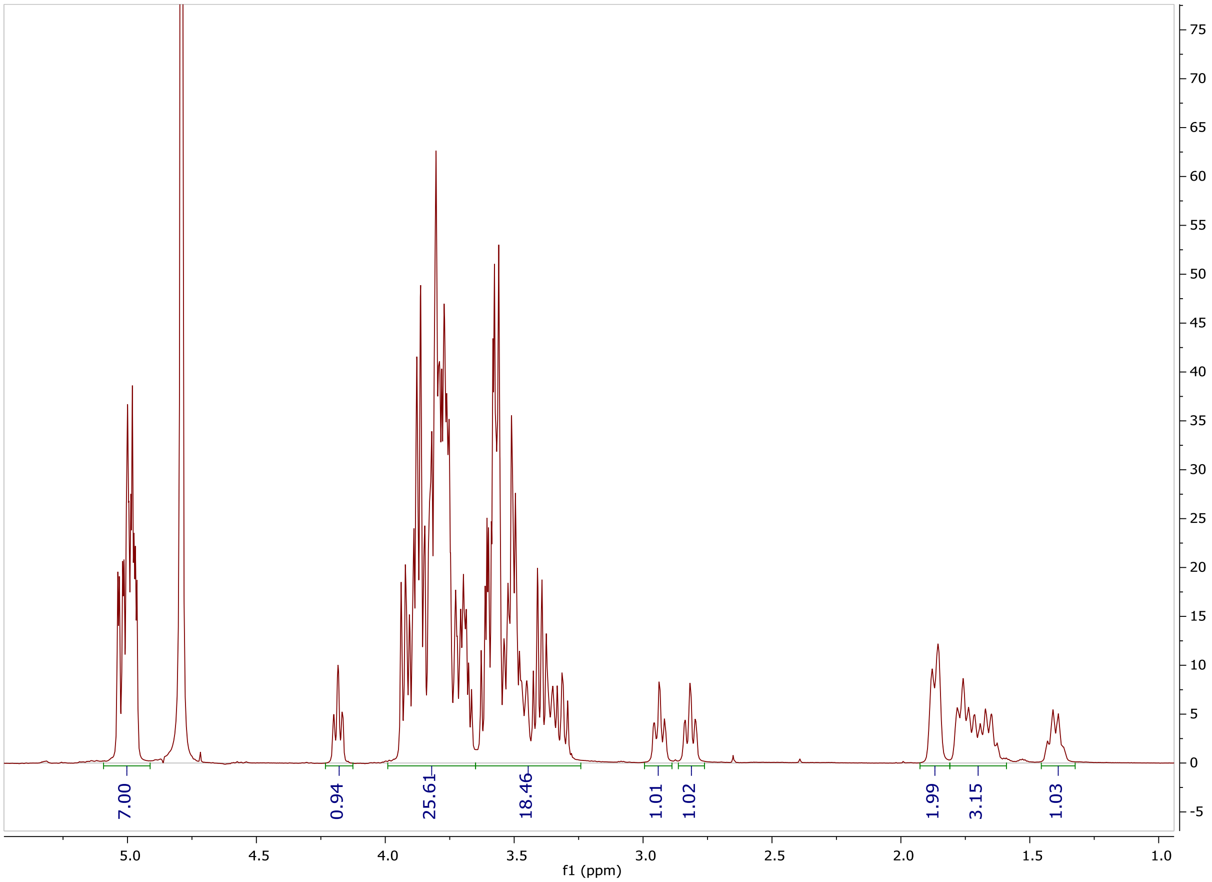

Figure S7. ^{1}H NMR spectrum of PIP-β-CD with integration (600 MHz, 298 K, D_2O).

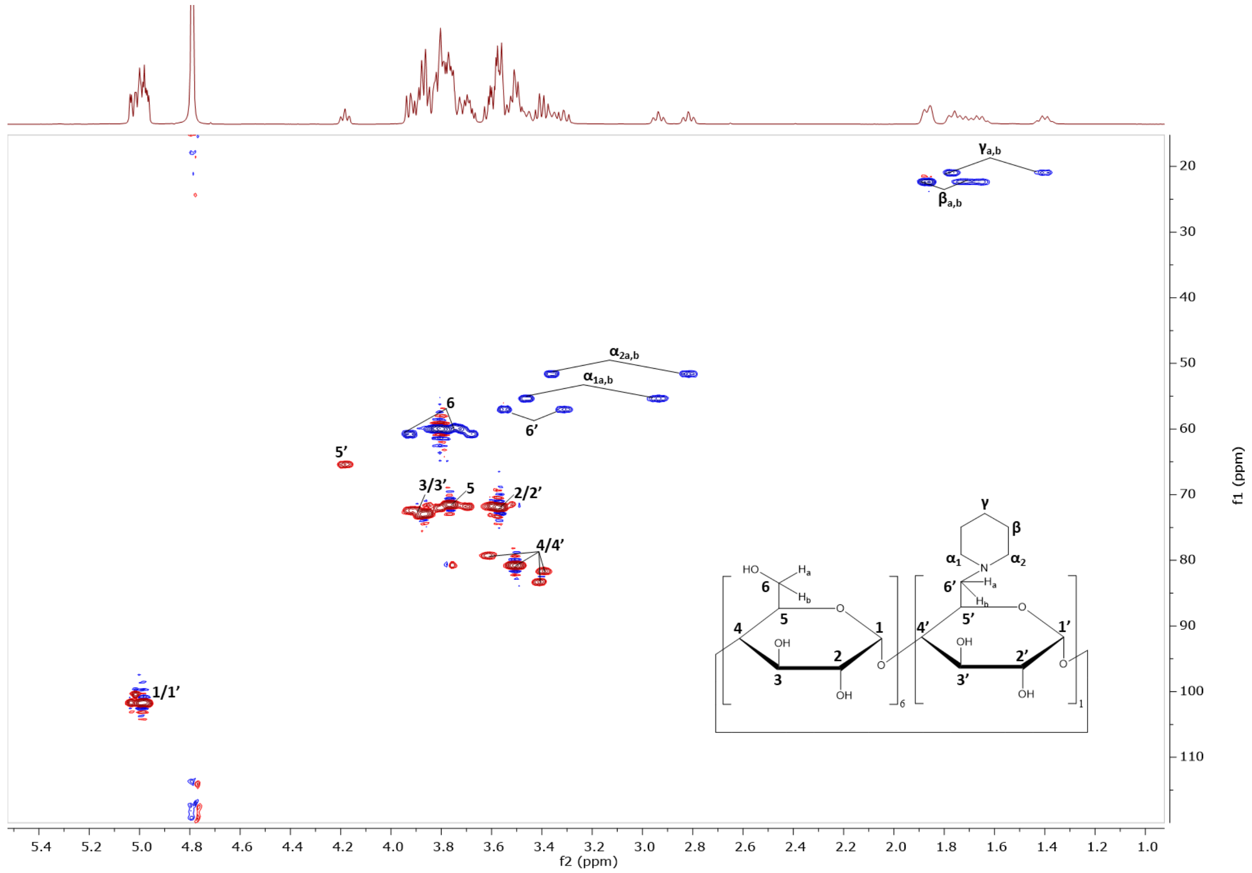

Figure S8. DEPT-edited HSQC spectrum of PIP-β-CD with assignment (600 MHz, 298 K, D_2O).

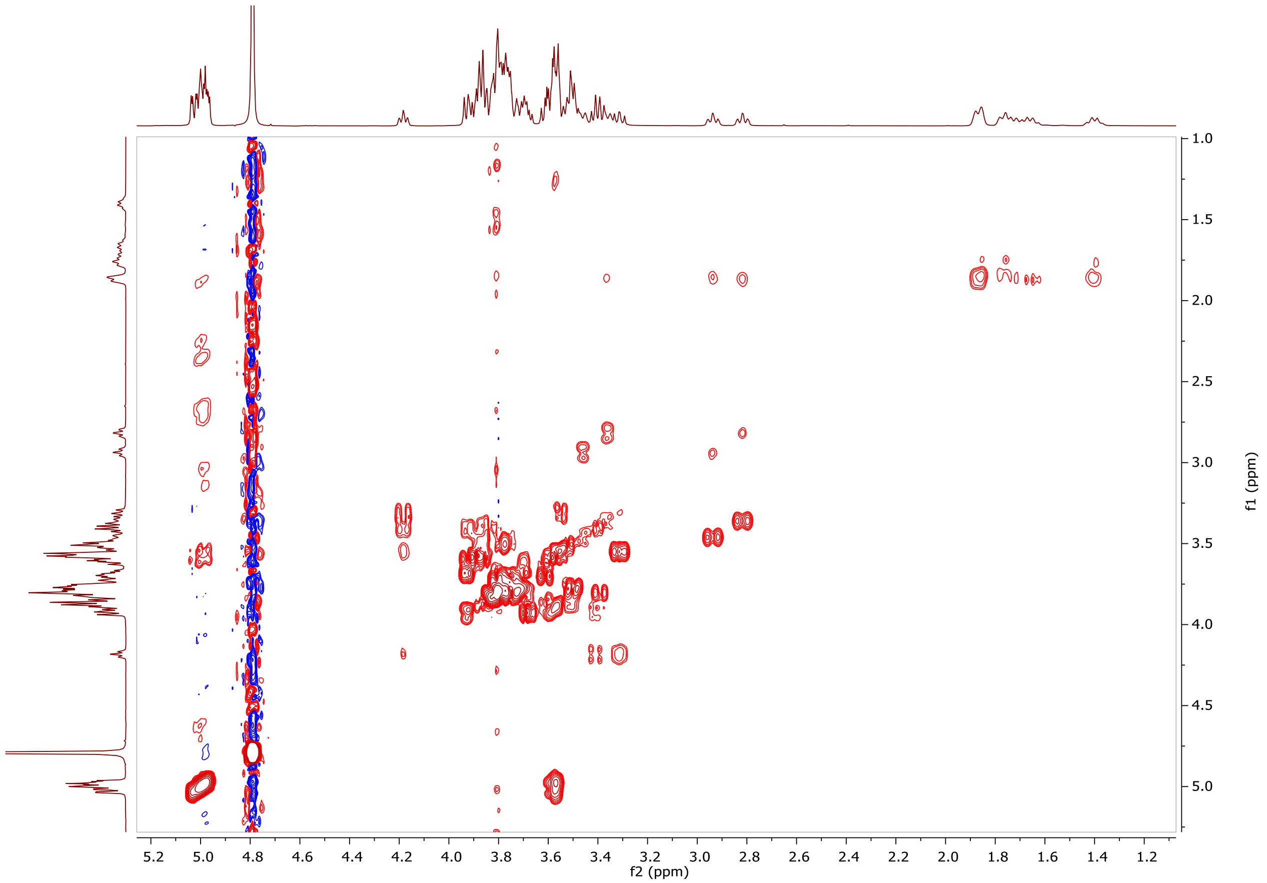

Figure S9. 2D COSY spectrum of PIP-β-CD (600 MHz, 298 K, D_2O).

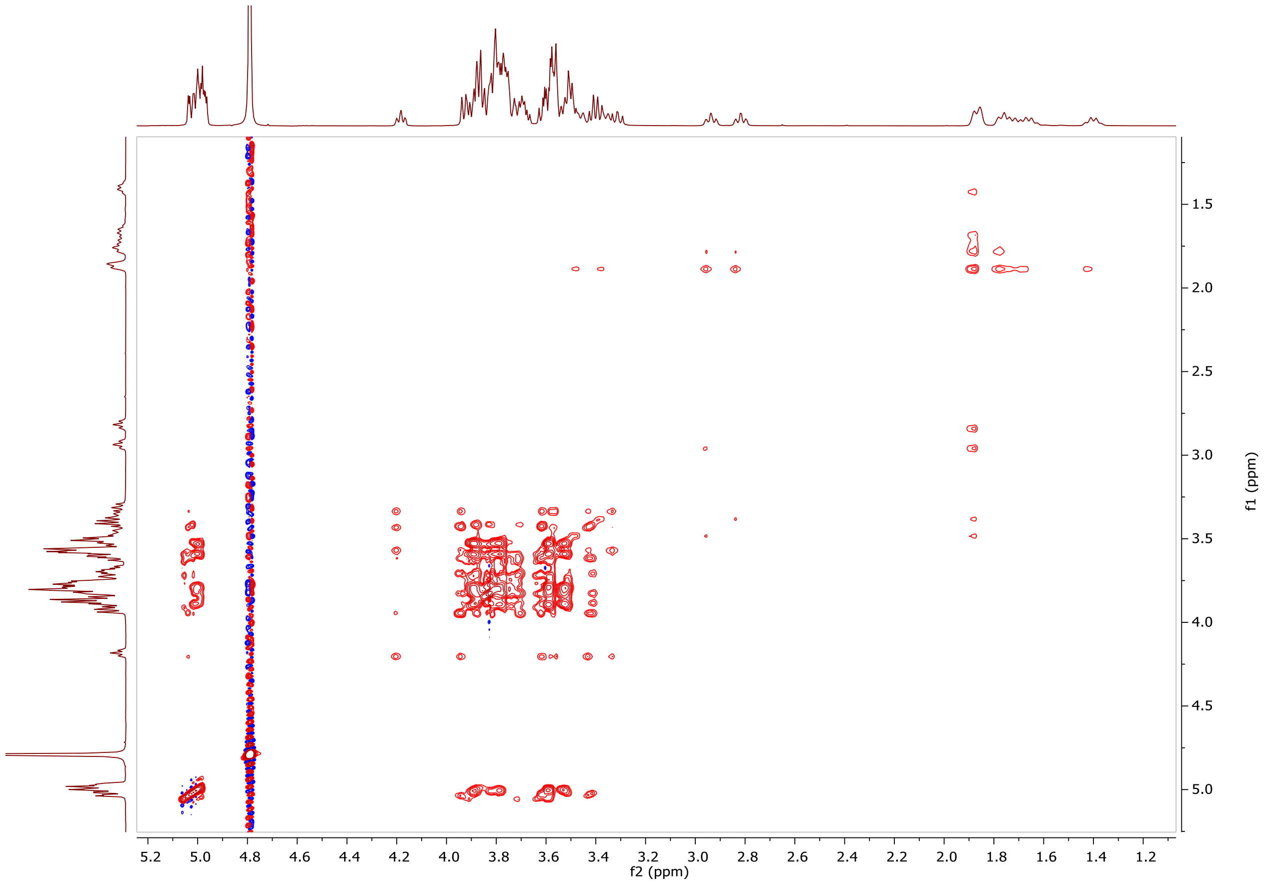

Figure S10. 2D TOCSY spectrum of PIP-β-CD (600 MHz, 298 K, D_2O).

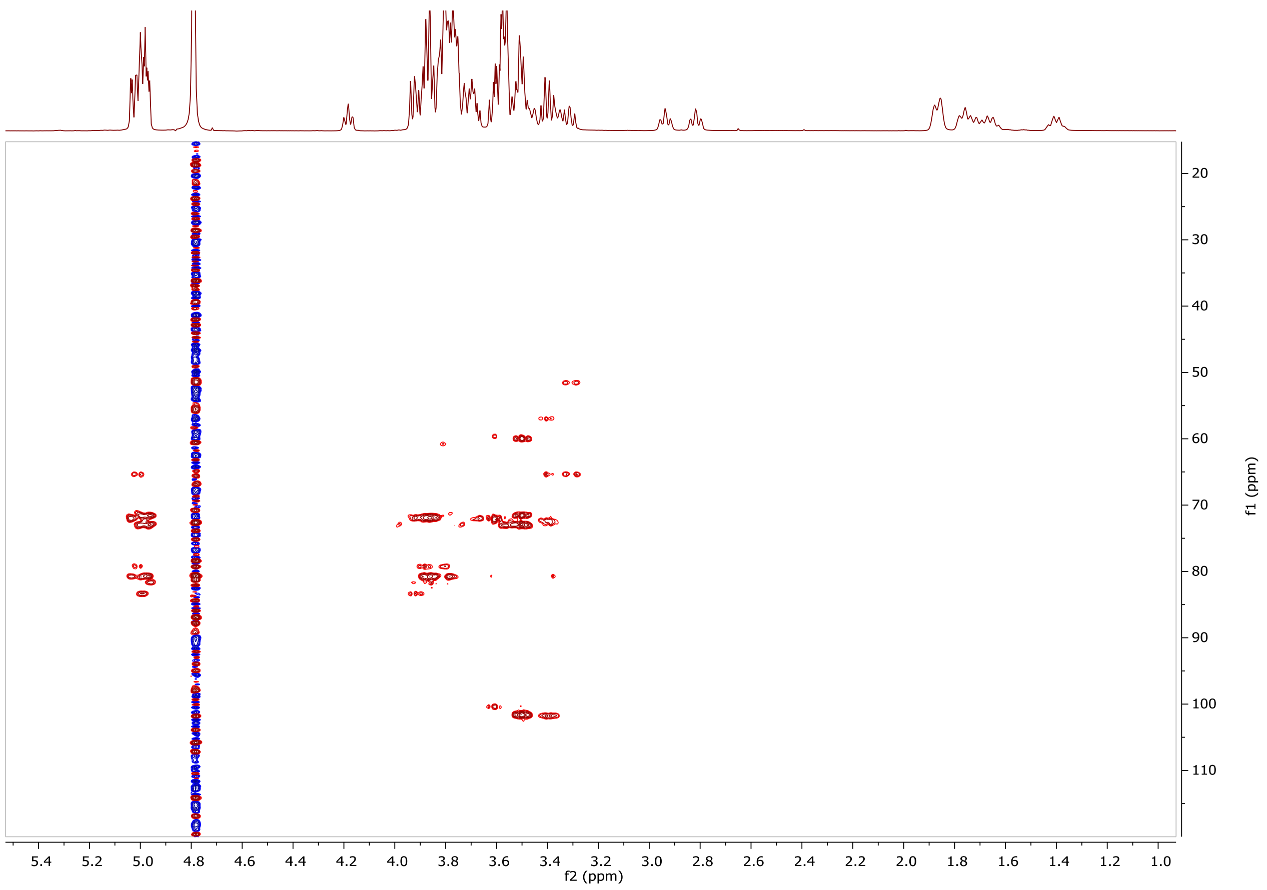

Figure S11. HMBC spectrum of PIP-β-CD (600 MHz, 298 K, D_2O).

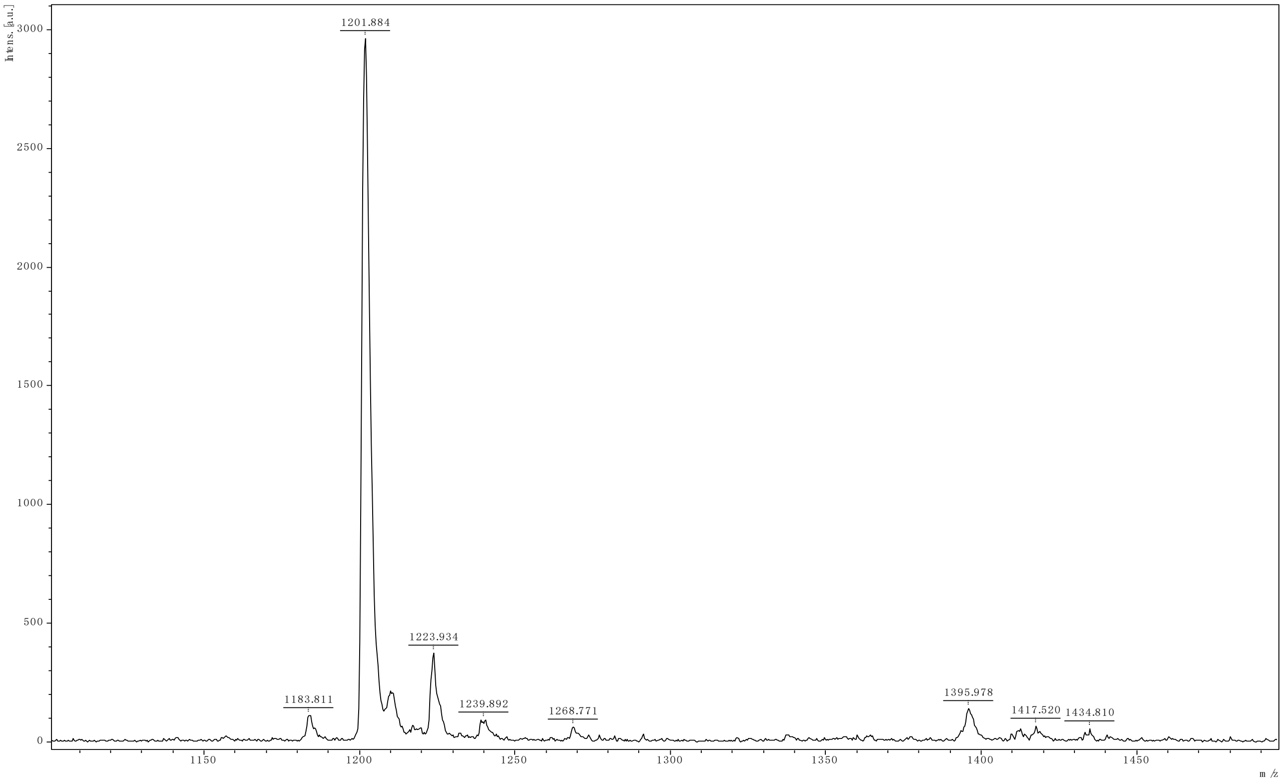

Figure S12. MALDI-TOF-MS spectrum of PIP-β-CD.

Mono-(6-*N*-morpholine-6-deoxy)-β-CD (MO-β-CD) (4)

^{1}H NMR (600 MHz, D_2O): δ (ppm) 5.15-5.01 (m, 7H, H-1), 4.35 (td, 3J = 9.9 Hz, 3J = 2.5 Hz, 1H, H-3), 4.21-4.06 (m, 2H, H-β(equ)), 4.06-3.37 (m, 46H, H-2, H-3, H-4, H-5, H-6, H-6’, H-β, H-α(equ)), 3.37-3.16 (m, 2H, H-α(ax)).

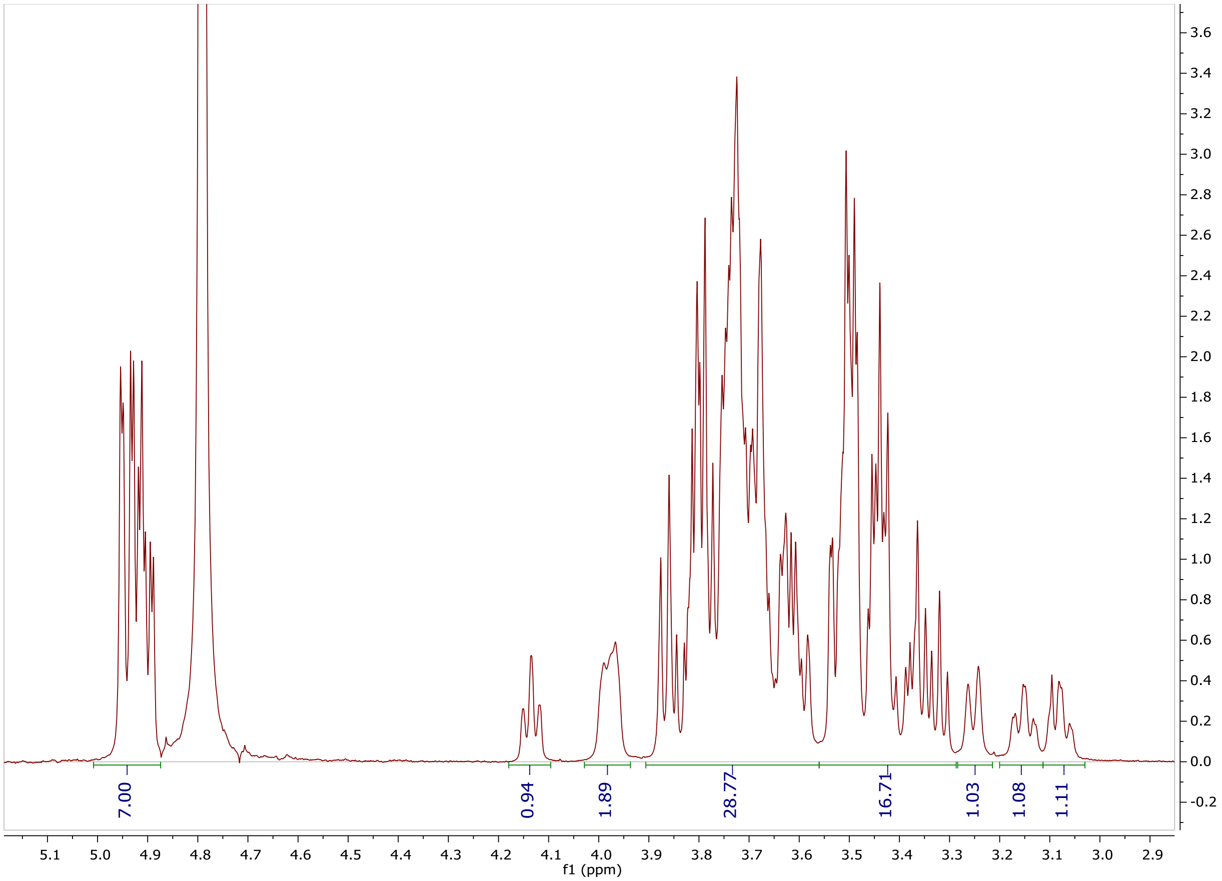

Figure S13. ^{1}H NMR spectrum of MO-β-CD with integration (600 MHz, 298 K, D_2O).

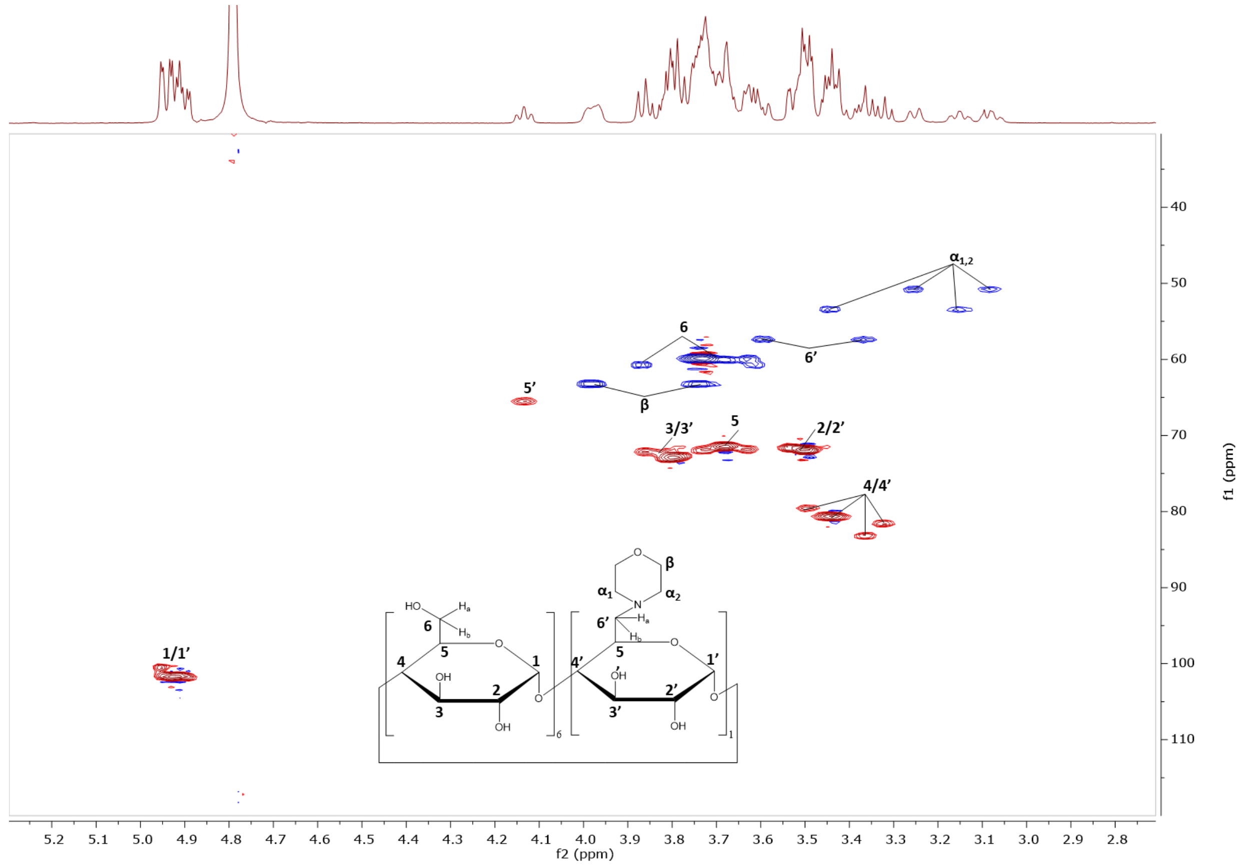

Figure S14. DEPT-edited HSQC spectrum of MO-β-CD with assignment (600 MHz, 298 K, D_2O).

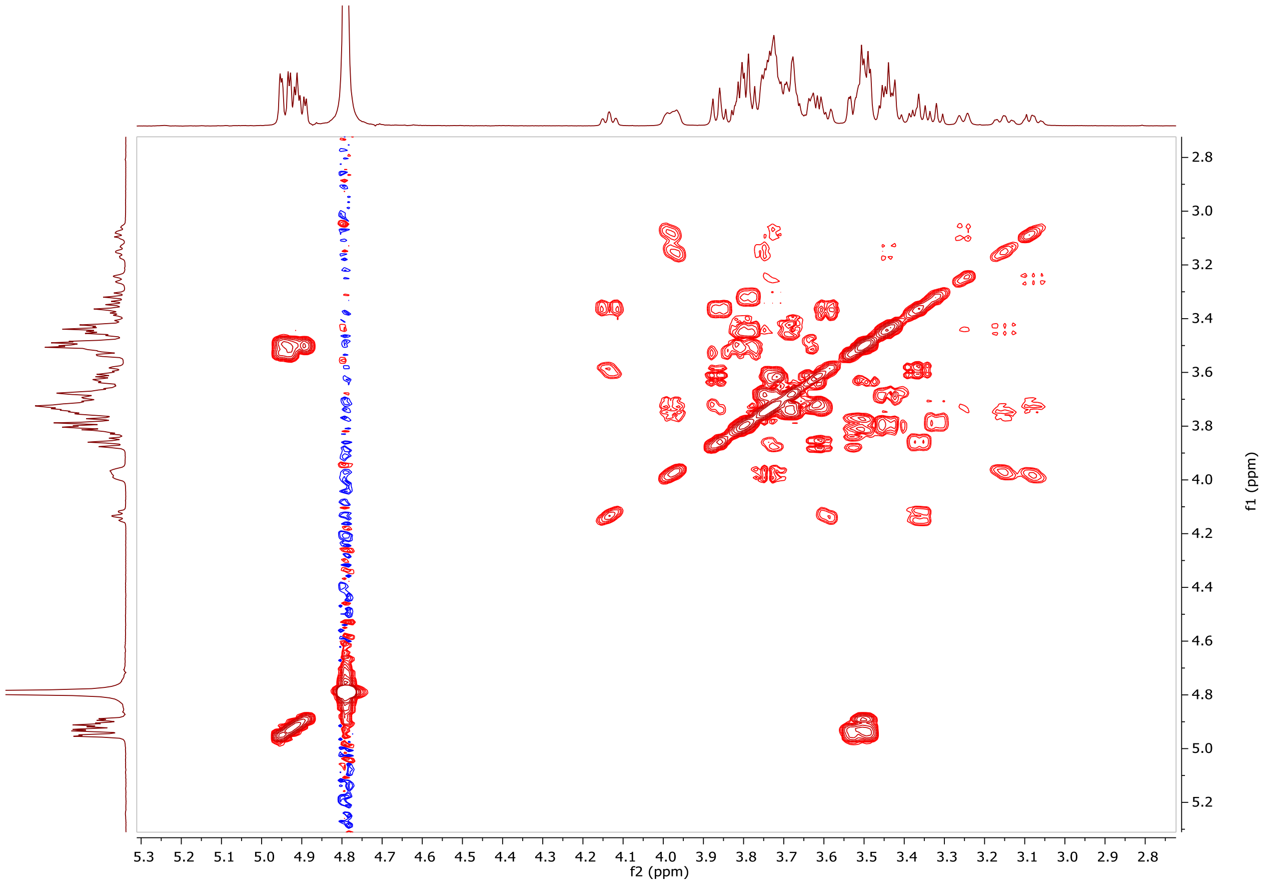

Figure S15. 2D COSY spectrum of MO-β-CD (600 MHz, 298 K, D_2O).

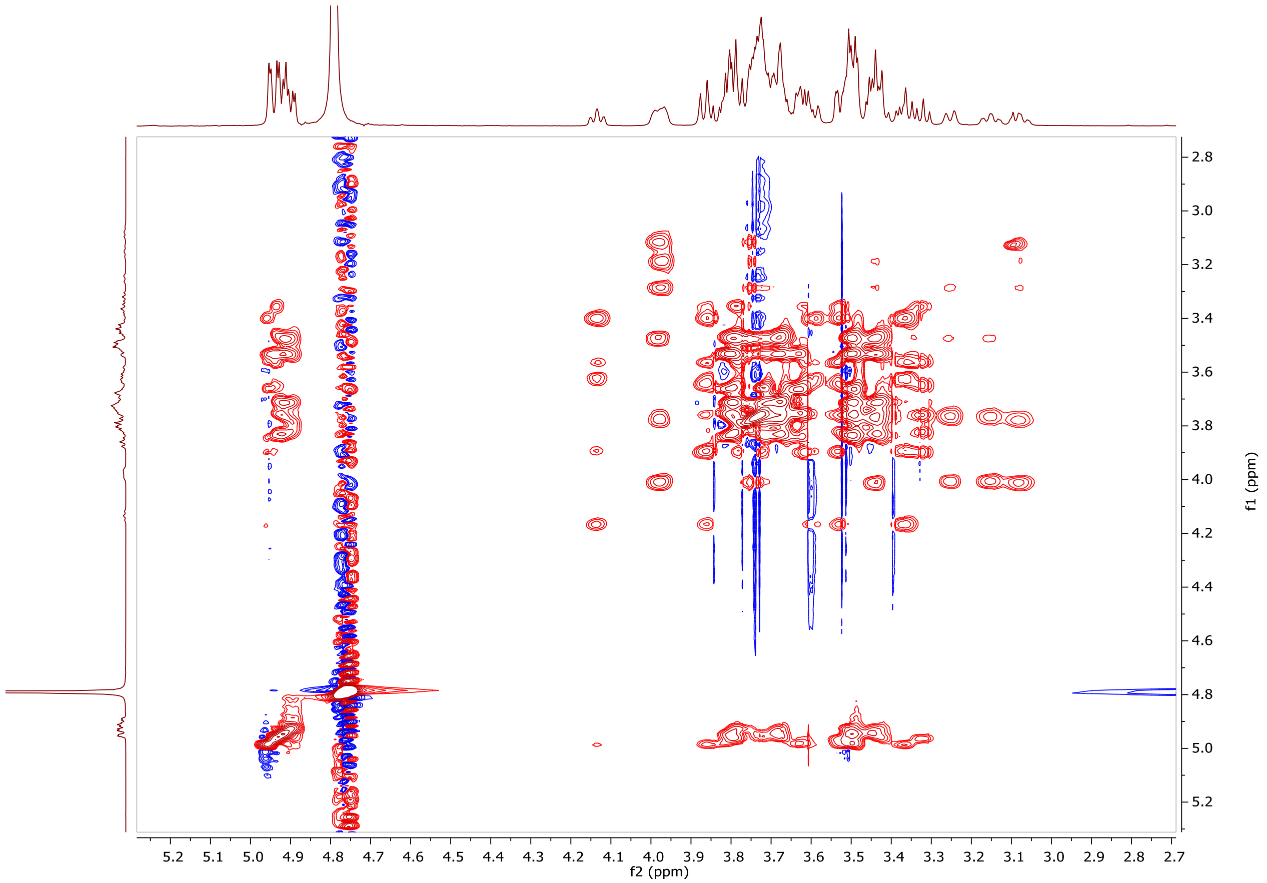

Figure S16. 2D TOCSY spectrum of MO-β-CD (600 MHz, 298 K, D_2O).

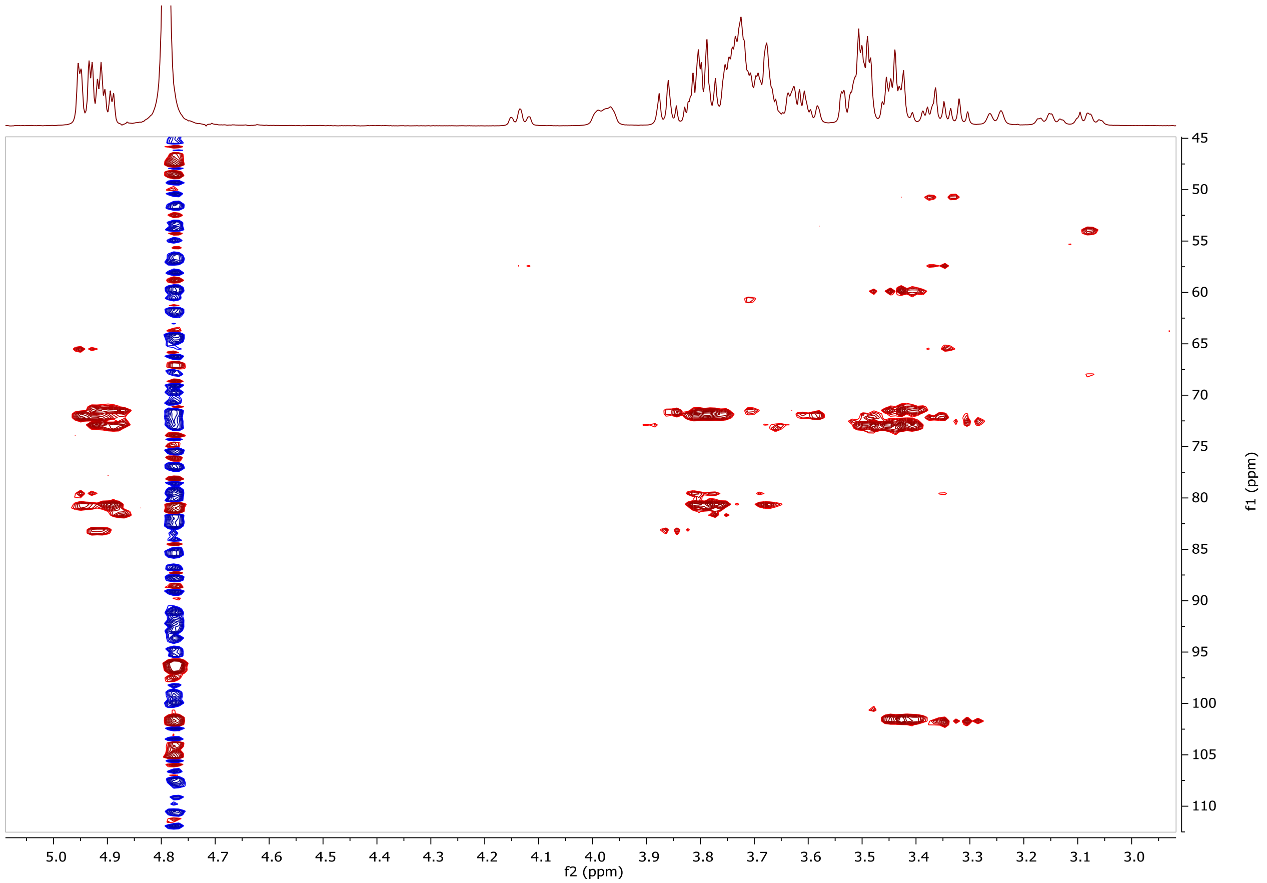

Figure S17. HMBC spectrum of MO-β-CD (600 MHz, 298 K, D_2O).

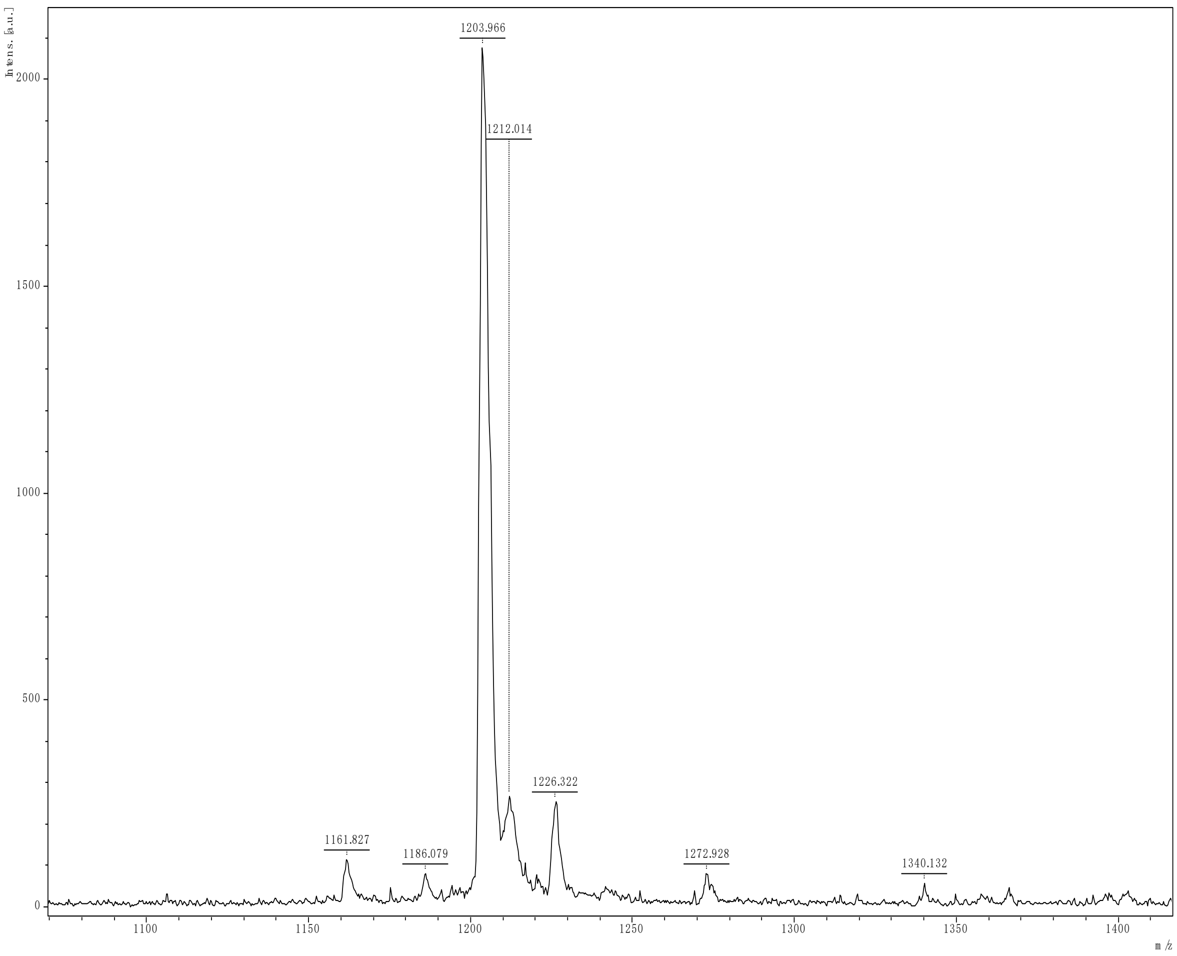

Figure S18. MALDI-TOF-MS spectrum of MO-β-CD.

Mono-(6-*N*-piperazine-6-deoxy)-β-CD (PIPA-β-CD) (5)

^{1}H NMR (600 MHz, D_2O): δ (ppm) 5.15-5.03 (m, 7H, H-1), 4.25 (t, 3J = 9.3 Hz, 1H, H-5'), 3.97 (t, 7H, H3), 3.94-3.82 (m, 19H, H-5, H-6), 3.71-3.63 (m, 7H, H-2), 3.63-3.54 (m, 6H, H-4), 3.54-3.48(t, 3J = 9,4 Hz, C4'), 3.33-3.21 (m, 4H, Hβ), 2.96-2.71 (m, 7H, C6', Cα, overlapping with traces of DMF).

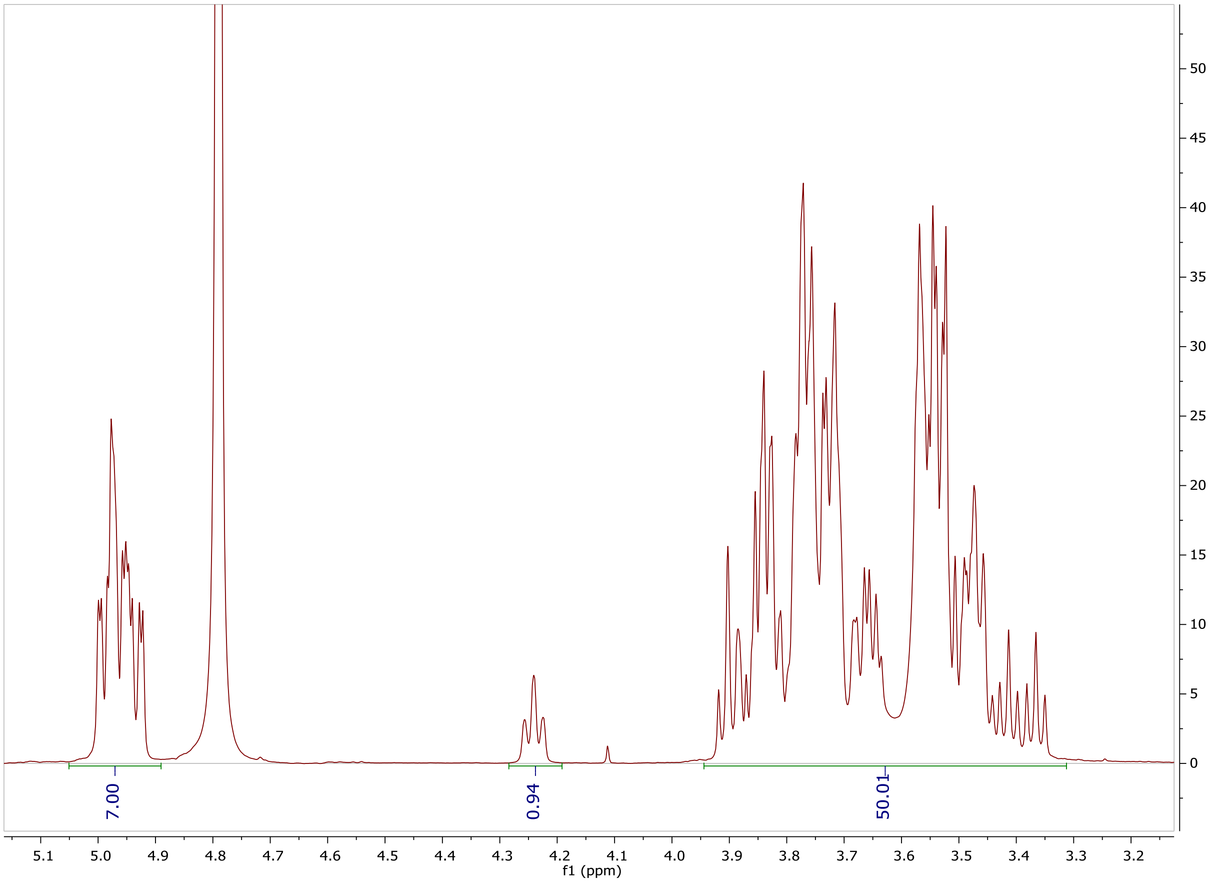

Figure S19. ^{1}H NMR spectrum of PIPA-β-CD with integration (600 MHz, 298 K, D_2O).

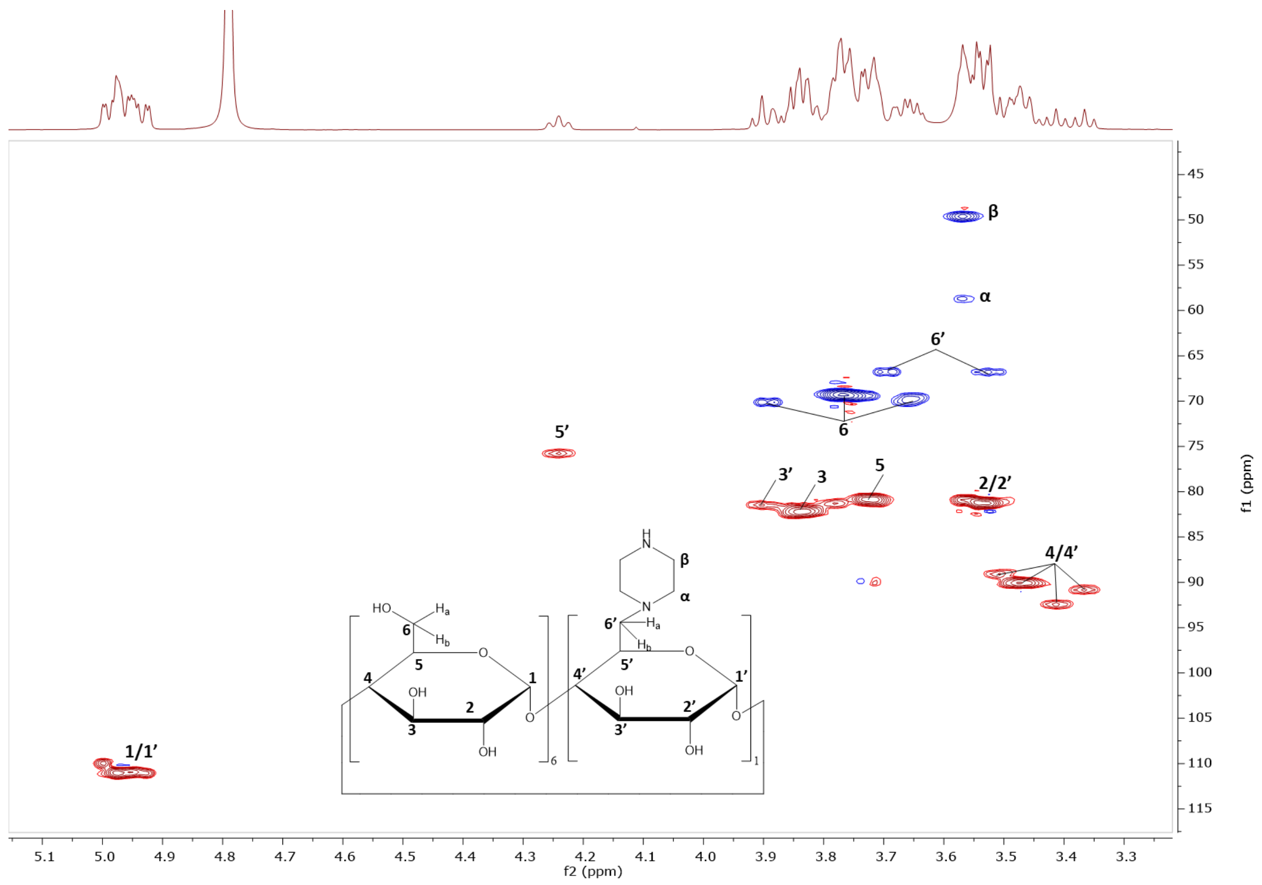

Figure S20. DEPT-edited HSQC spectrum of PIPA-β-CD with assignment (600 MHz, 298 K, D_2O).

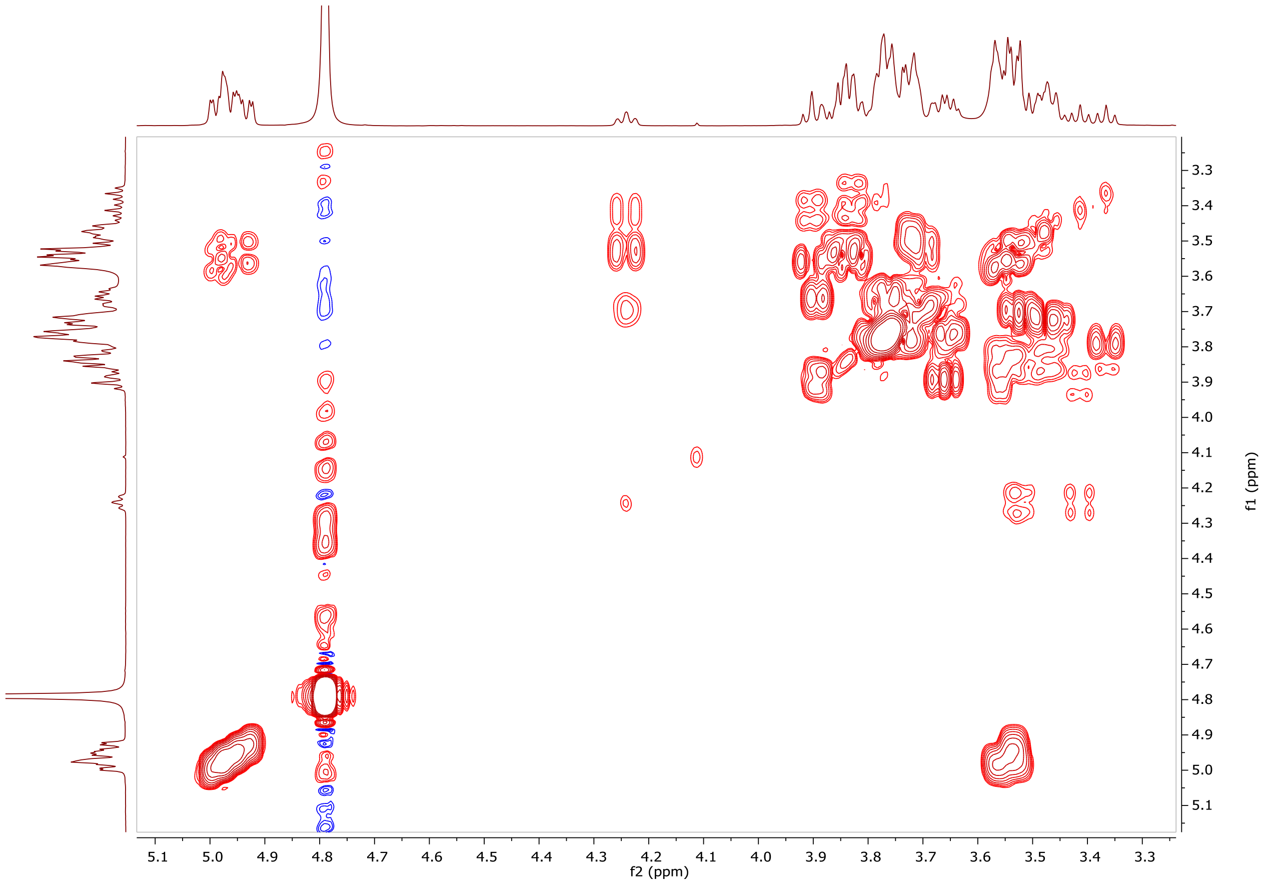

Figure S21. 2D COSY spectrum of PIPA-β-CD (600 MHz, 298 K, D_2O).

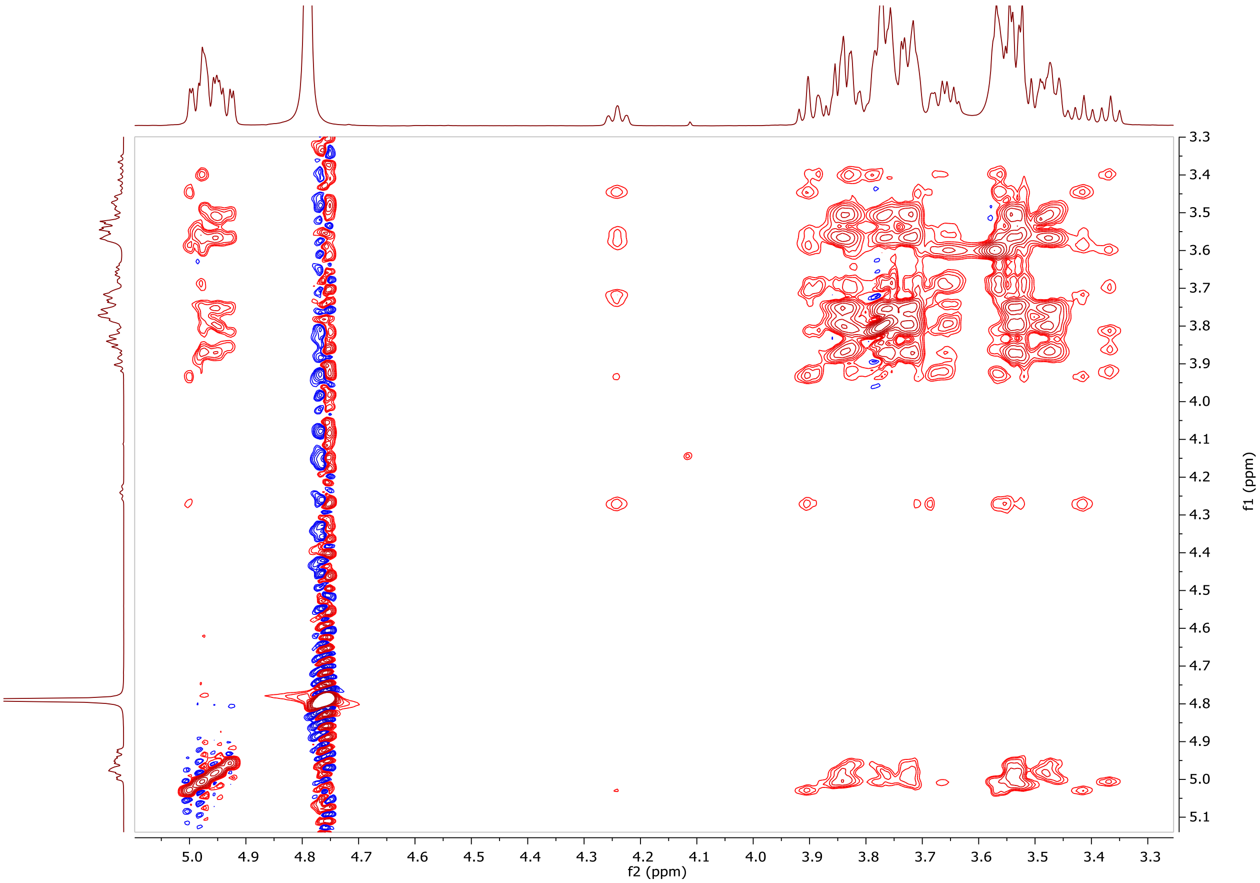

Figure S22. 2D TOCSY spectrum of PIPA-β-CD (600 MHz, 298 K, D_2O).

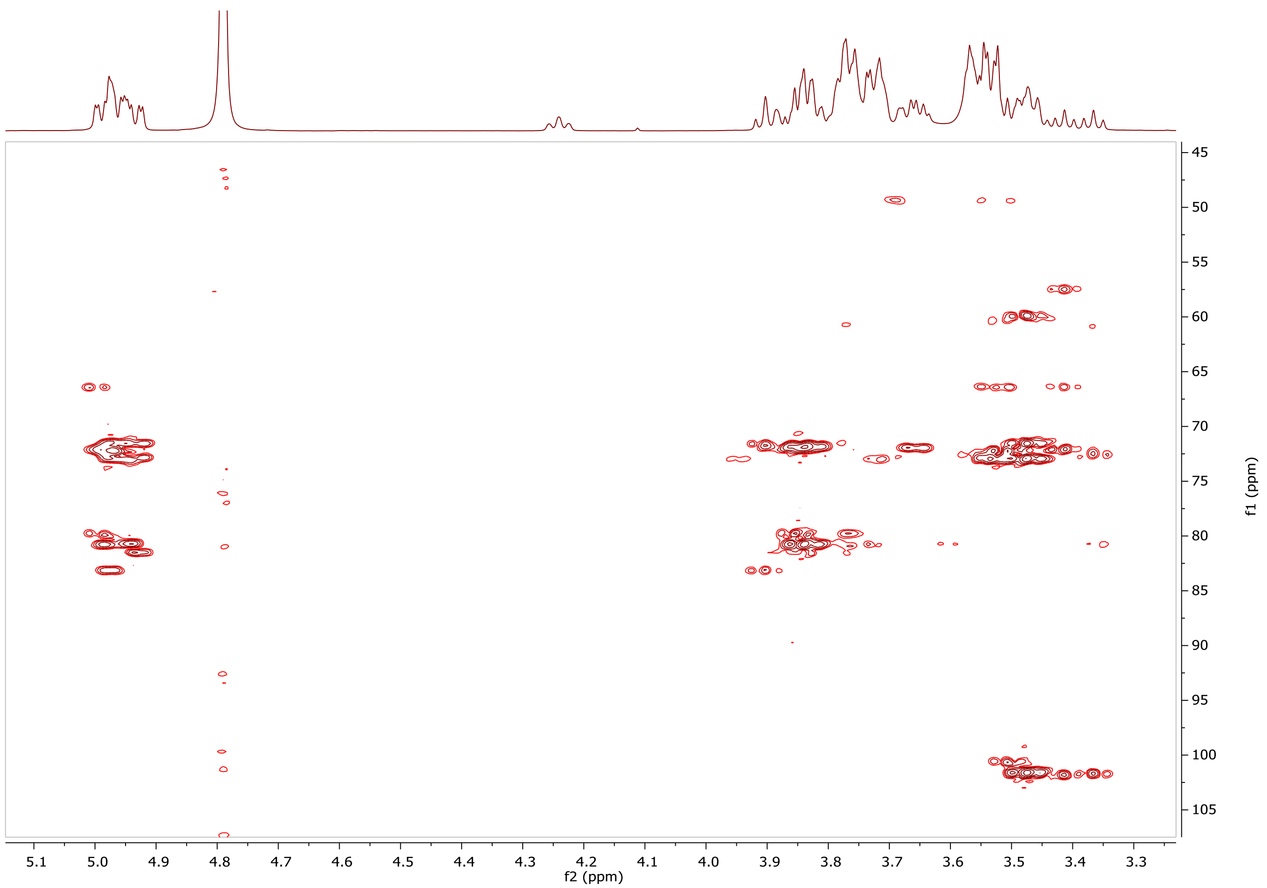

Figure S23. HMBC spectrum of PIPA-β-CD (600 MHz, 298 K, D_2O).

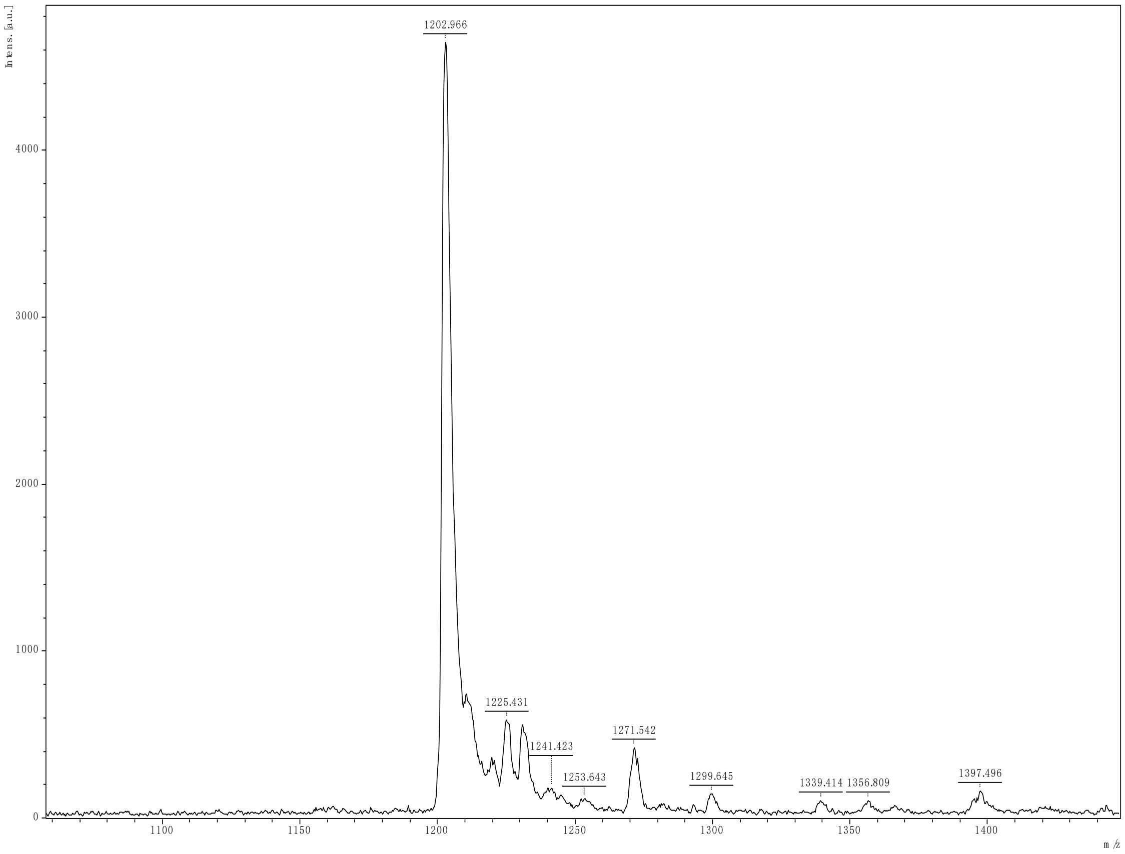

Figure S24. MALDI-TOF-MS spectrum of PIPA-β-CD.

Mono-(6-*N*-(*N*-methylpyrrolidine)-6-deoxy)-β-CD (MePYR-β-CD (6)

^{1}H NMR (600 MHz, D_2O): δ (ppm) 5.23-5.01 (m, 7H, H-1), 4.51 (t, 3J = 9.4 Hz, 1H, H-5’), 4.16-3.46 (m, 45H, H-2, H-3, H-4, H-5, H-6, H-α), 3.15 (s, 3H, CH_3), 2.33-2.16 (m, 4H, H-β).

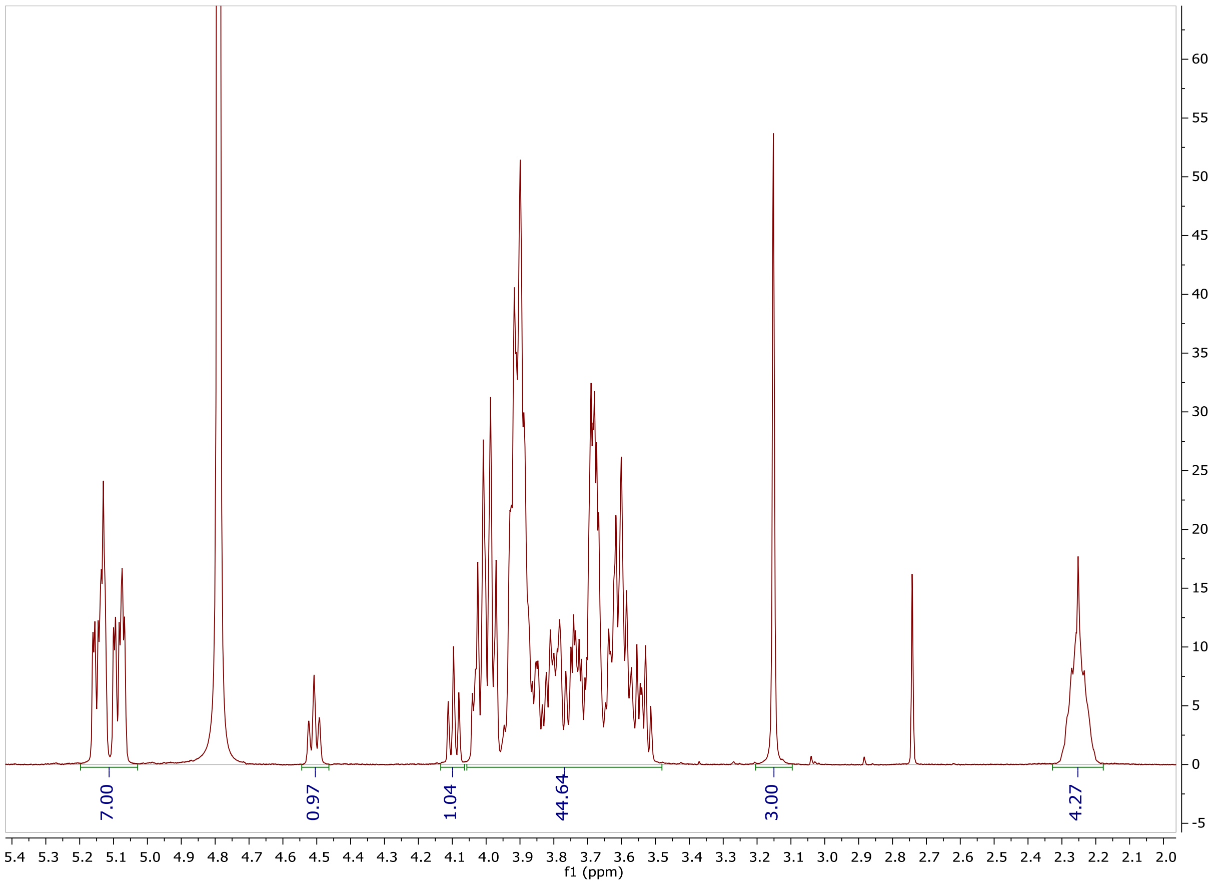

Figure S25. ^{1}H NMR spectrum of MePYR-β-CD with integration (600 MHz, 298 K, D_2O).

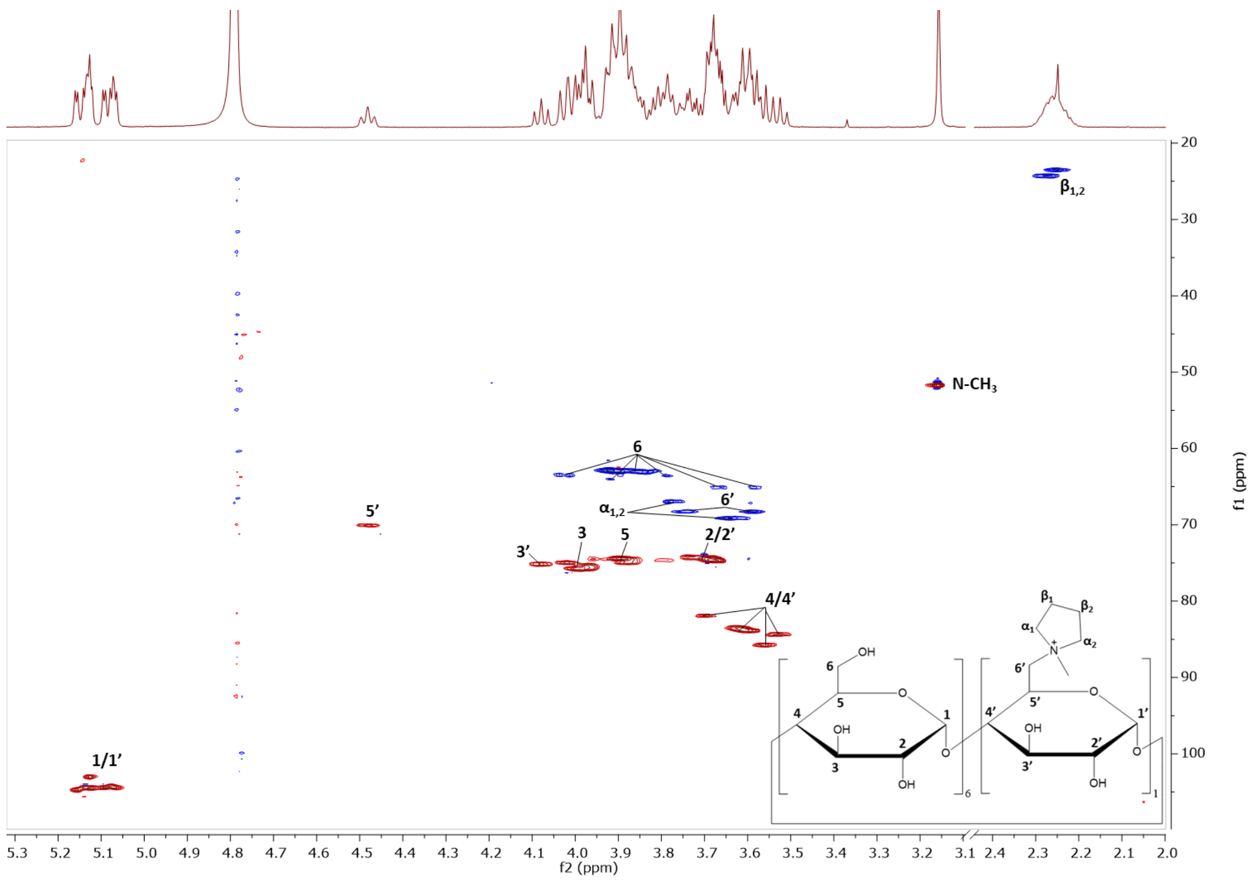

Figure S26. DEPT-edited HSQC spectrum of Me-PYR-β-CD with assignment (600 MHz, 298 K, D_2O).

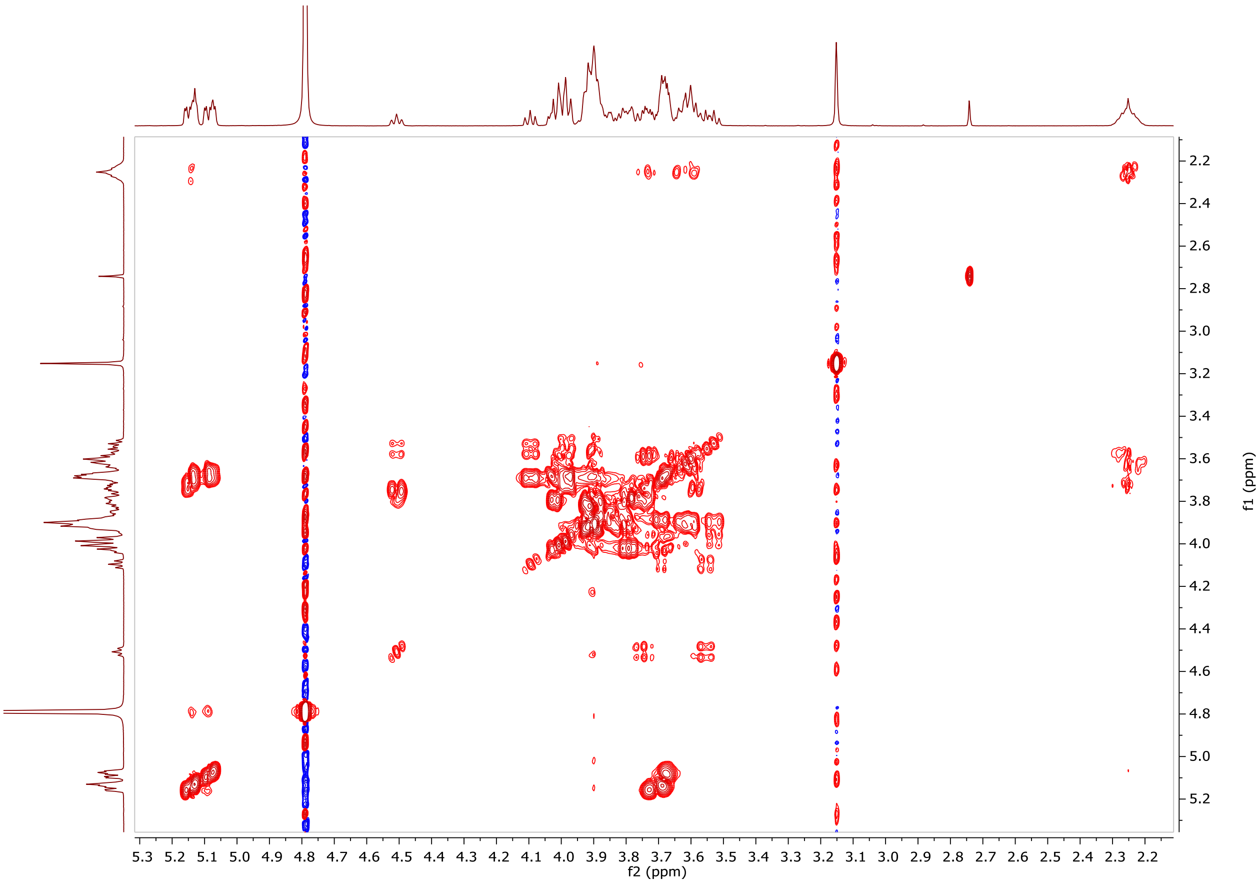

Figure S27. 2D COSY spectrum of Me-PYR-β-CD (600 MHz, 298 K, D_2O).

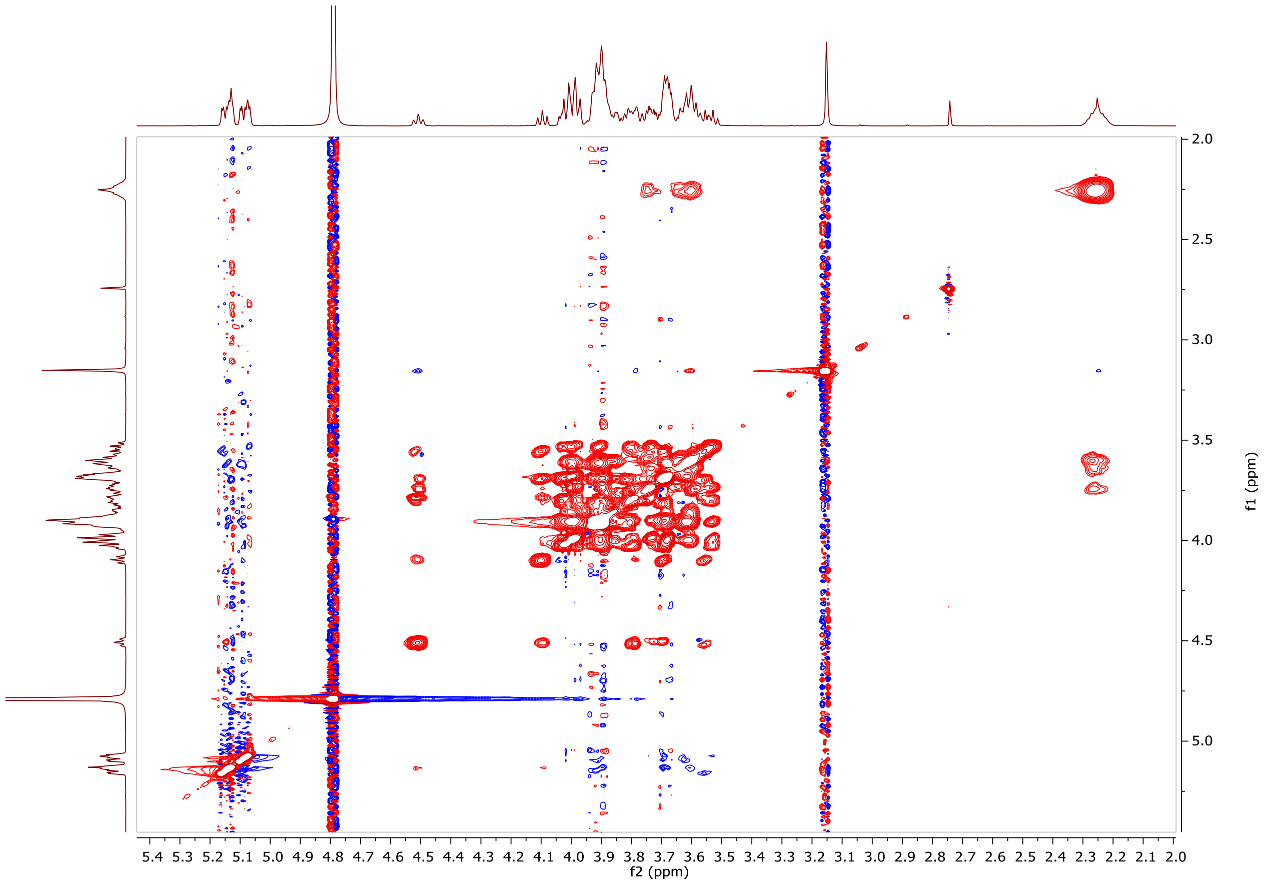

Figure S28. 2D TOCSY HSQC spectrum of Me-PYR-β-CD (600 MHz, 298 K, D_2O).

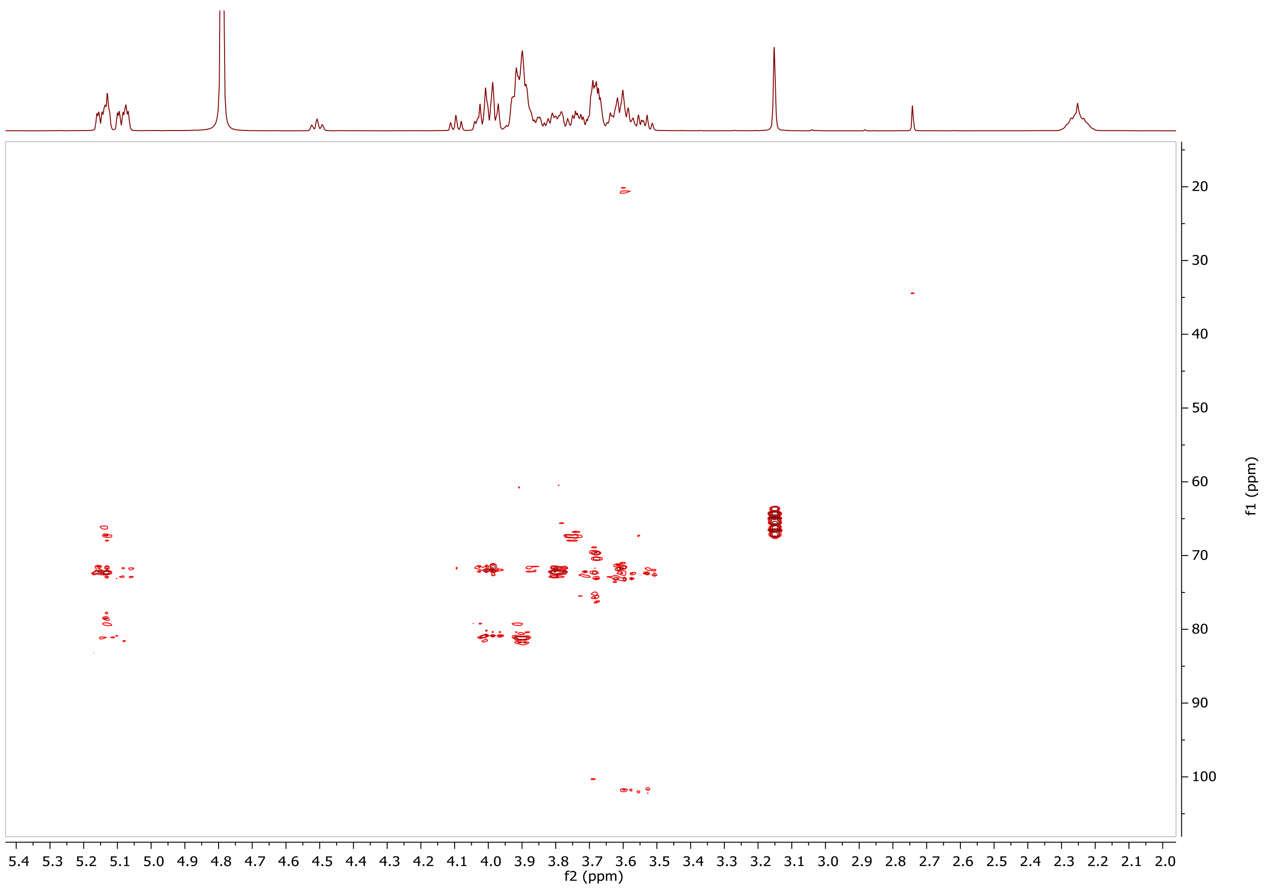

Figure S29. HMBC spectrum of Me-PYR-β-CD (600 MHz, 298 K, D_2O).

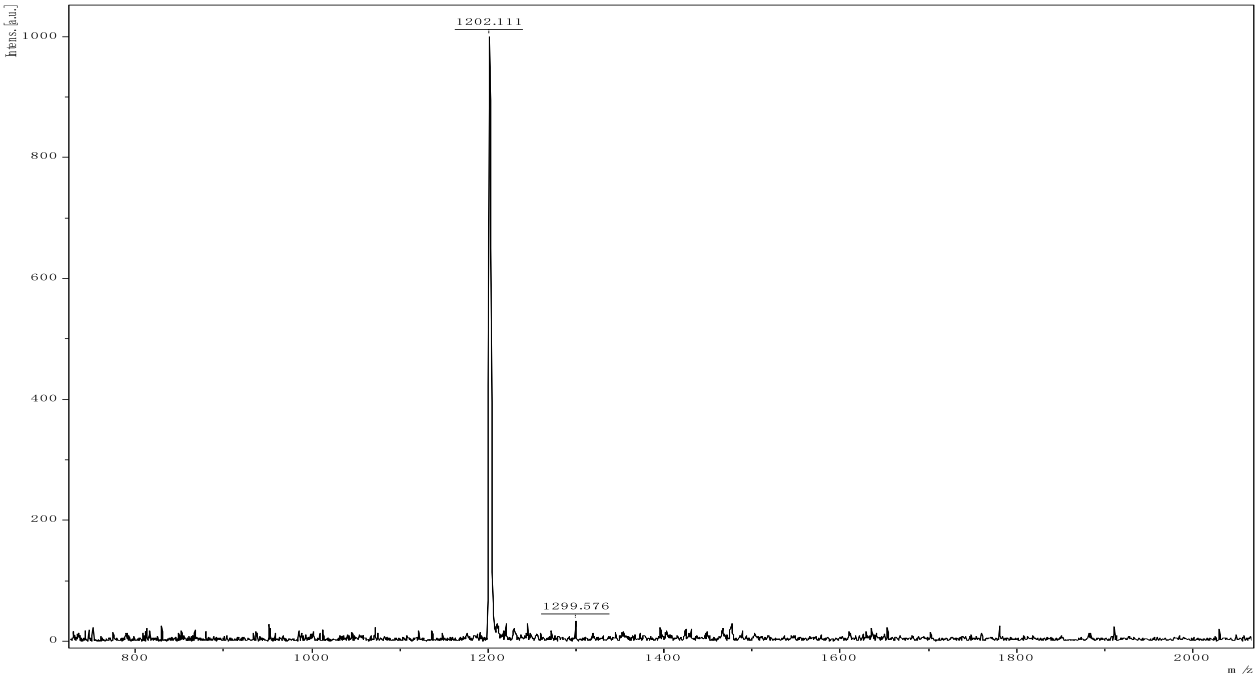

Figure S30. MALDI-TOF-MS spectrum of Me-PYR-β-CD.

Mono-(6-*N*-(*N*-methylpiperidine)-6-deoxy)-β-CD (MePIP-β-CD) (7)

^{1}H NMR (600 MHz, D_2O): δ (ppm) 5.22-5.02 (m, 7H, H-1), 4.55 (t, 3J = 9.5 Hz, 1H, H-3), 4.15-3.36 (m, 46H, H-2, H-3, H-4, H-6, H-6', H-α), 3.19 (s, 3H, CH_3), 2.02-1.86 (m, 4H, H-β), 1.78-1.57 (m, 2H, H-γ).

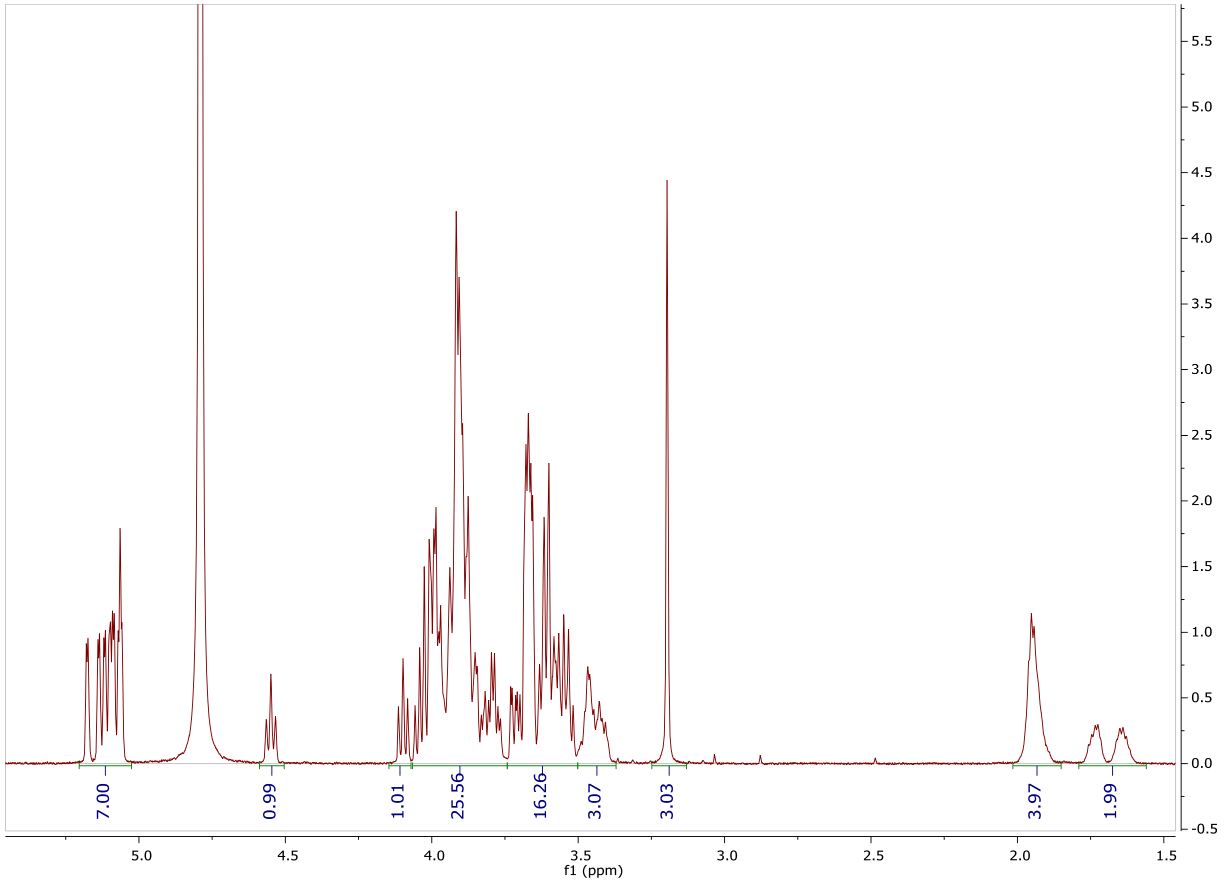

Figure S31. ^{1}H NMR spectrum of MePIP-β-CD with integration (600 MHz, 298 K, D_2O).

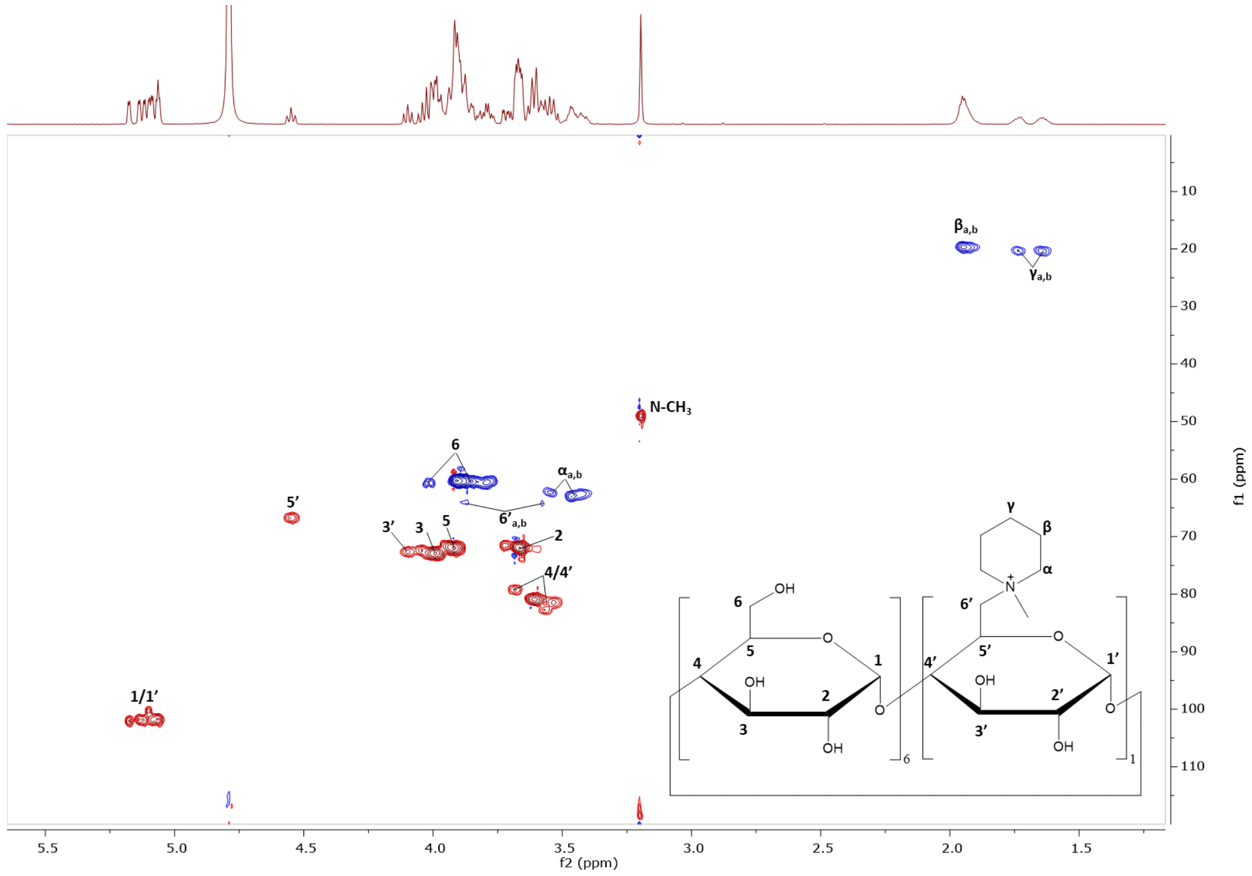

Figure S32. DEPT-edited HSQC spectrum of MePIP-β-CD with assignment (600 MHz, 298 K, D_2O).

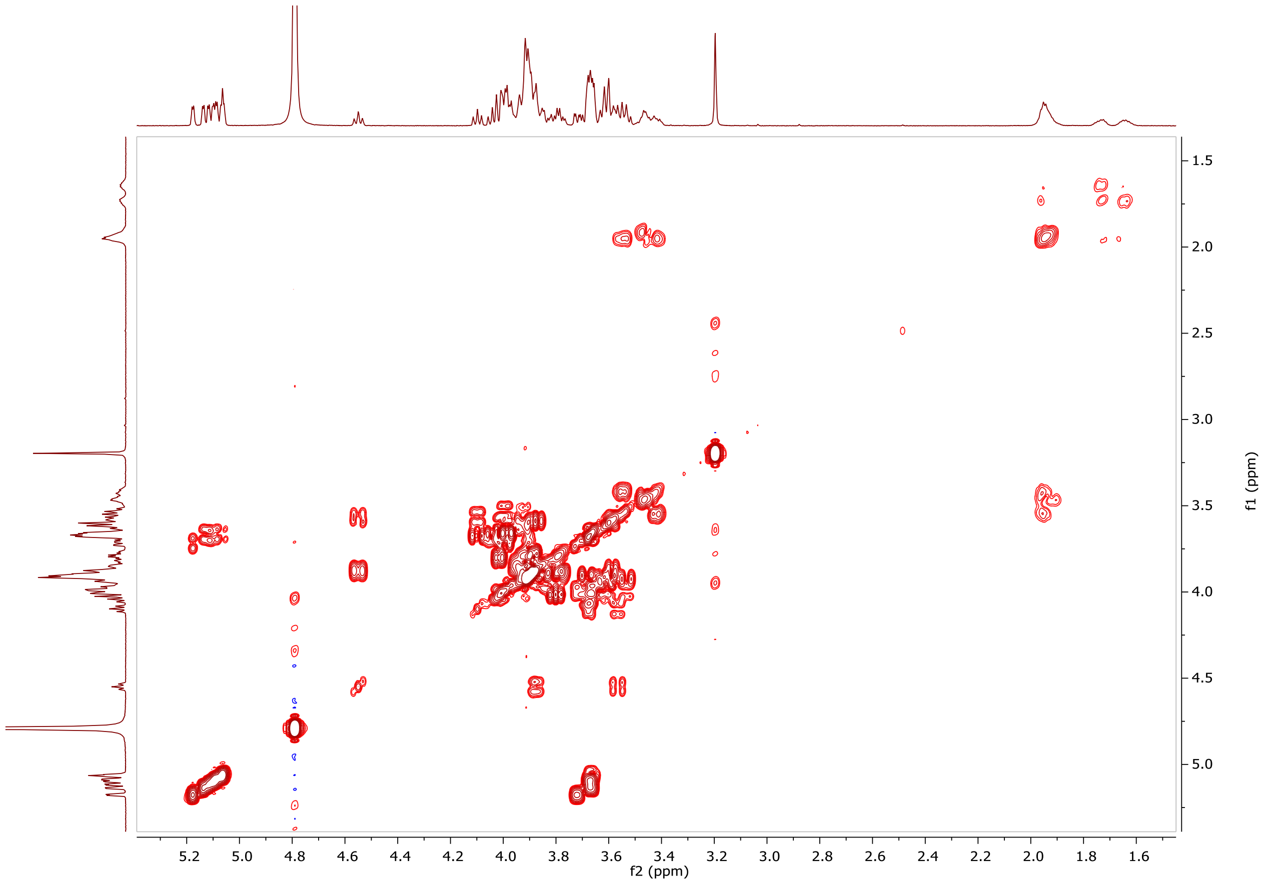

Figure S33. 2D COSY spectrum of MePIP-β-CD (600 MHz, 298 K, D_2O).

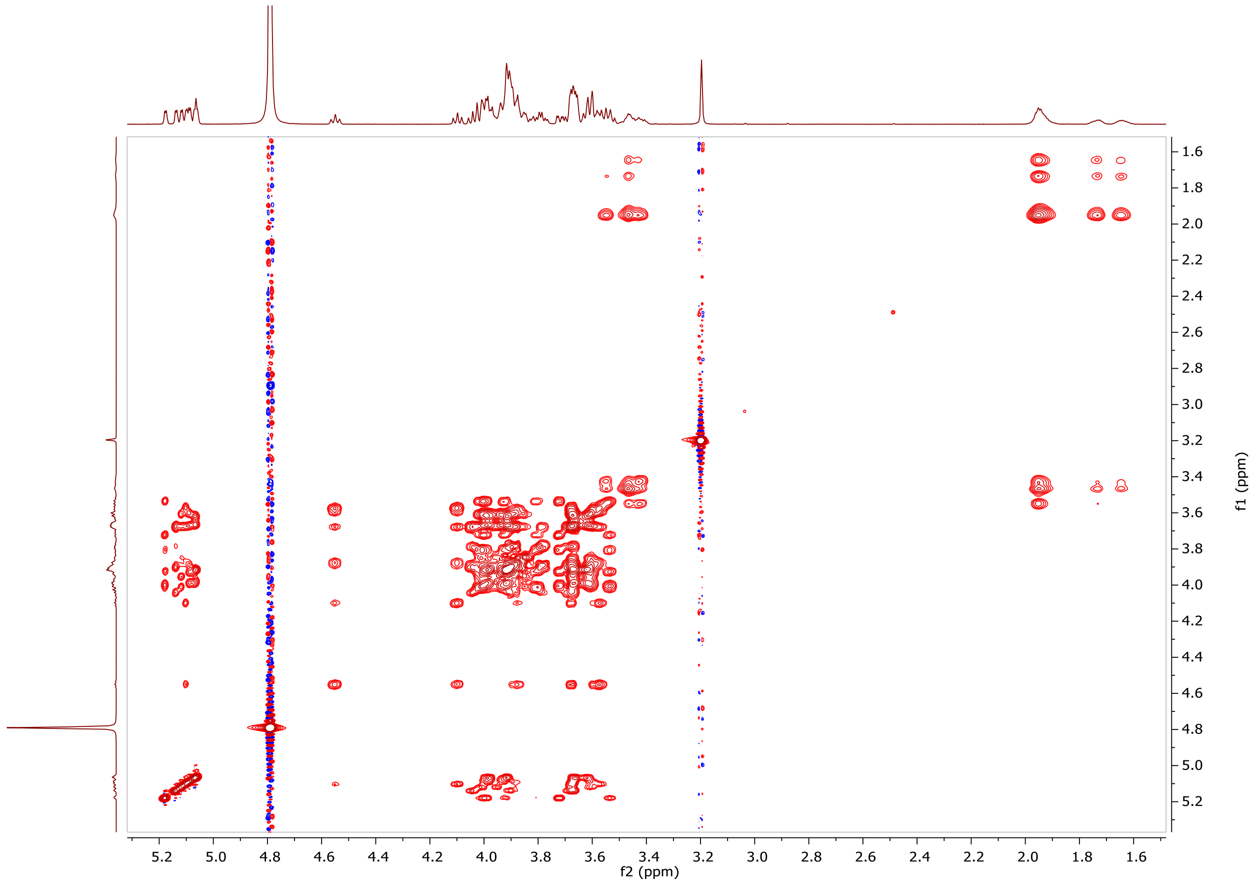

Figure S34. 2D TOCSY spectrum of MePIP-β-CD (600 MHz, 298 K, D_2O).

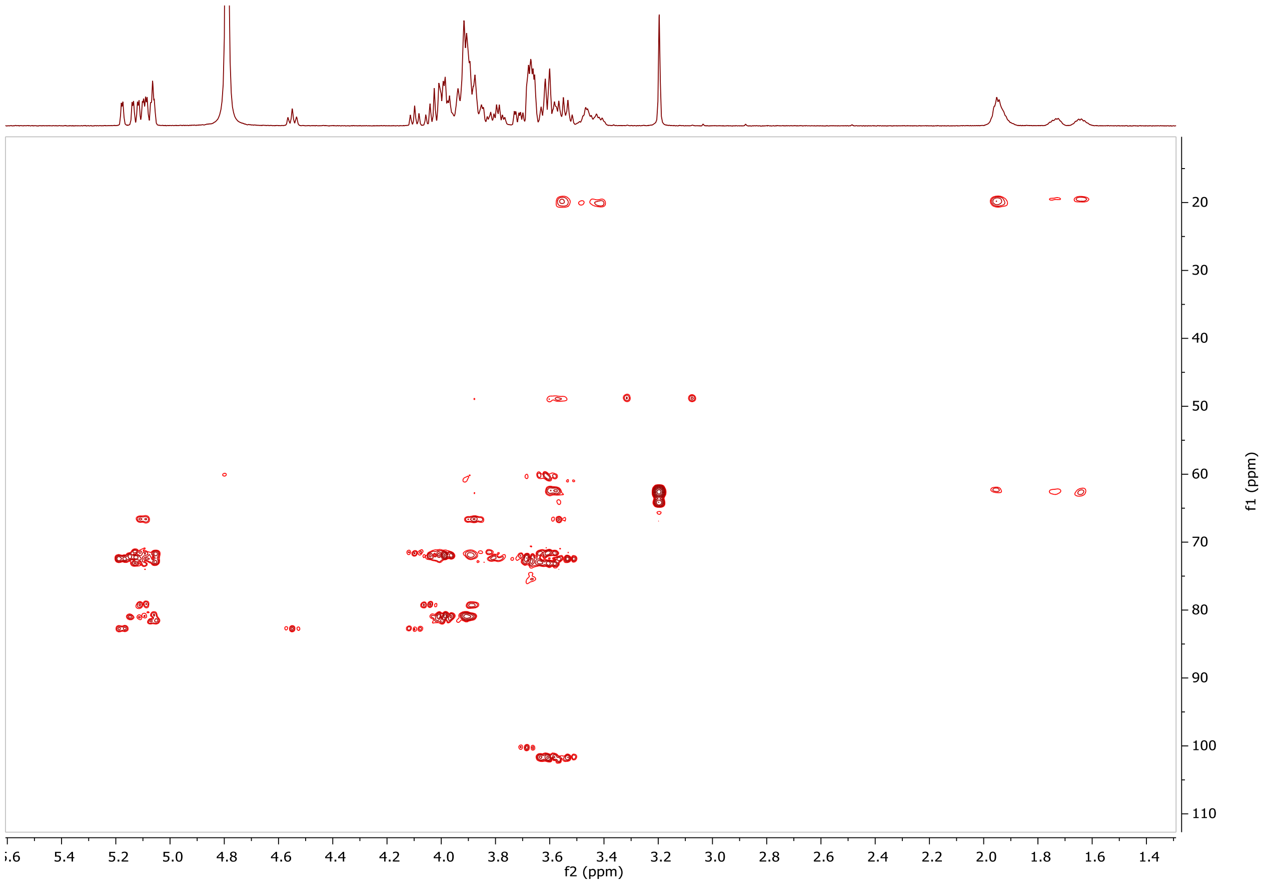

Figure S35. HMBC spectrum of MePIP-β-CD (600 MHz, 298 K, D_2O).

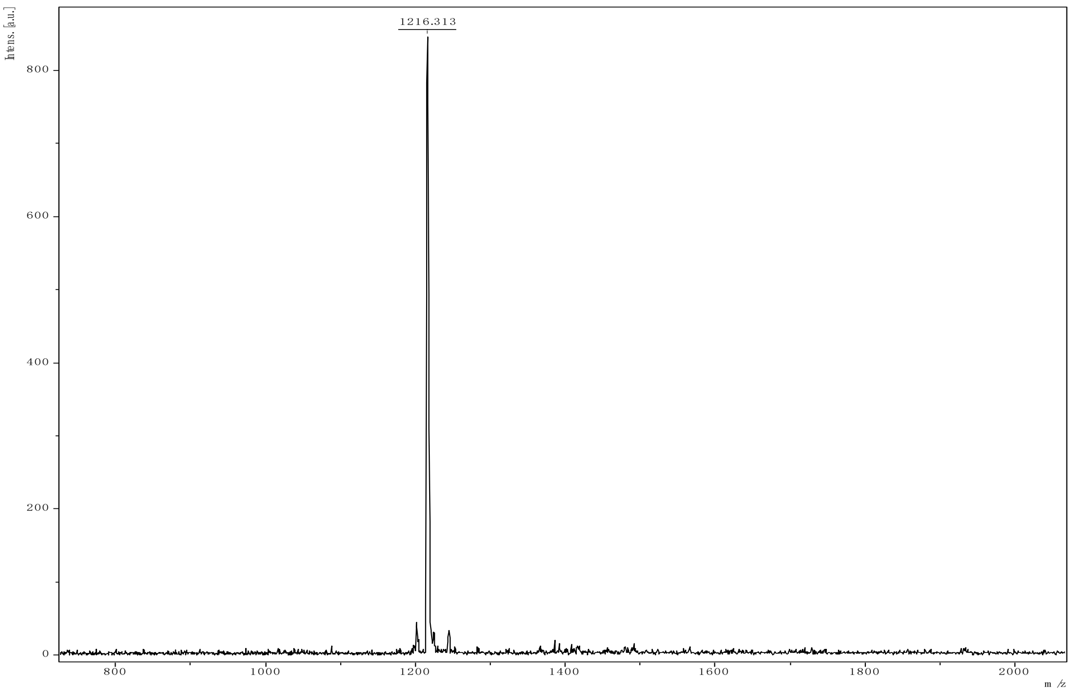

Figure S36. MALDI-TOF-MS spectrum of MePIP-β-CD.

Mono-(6-*N*-(*N*-methylmorpholin)-6-deoxy)-β-CD (MeMO-β-CD) (8)

^{1}H NMR (600 MHz, D_2O): δ (ppm) 5.21-5.02 (m, 7H, H-1), 4.61 (t, 3J = 9.5 Hz, 1H, H-3'), 4.16-3.45 (m, 50H, H-2, H-3, H-4, H-5, H-α, H-β), 3.36 (s, 3H, CH_3).

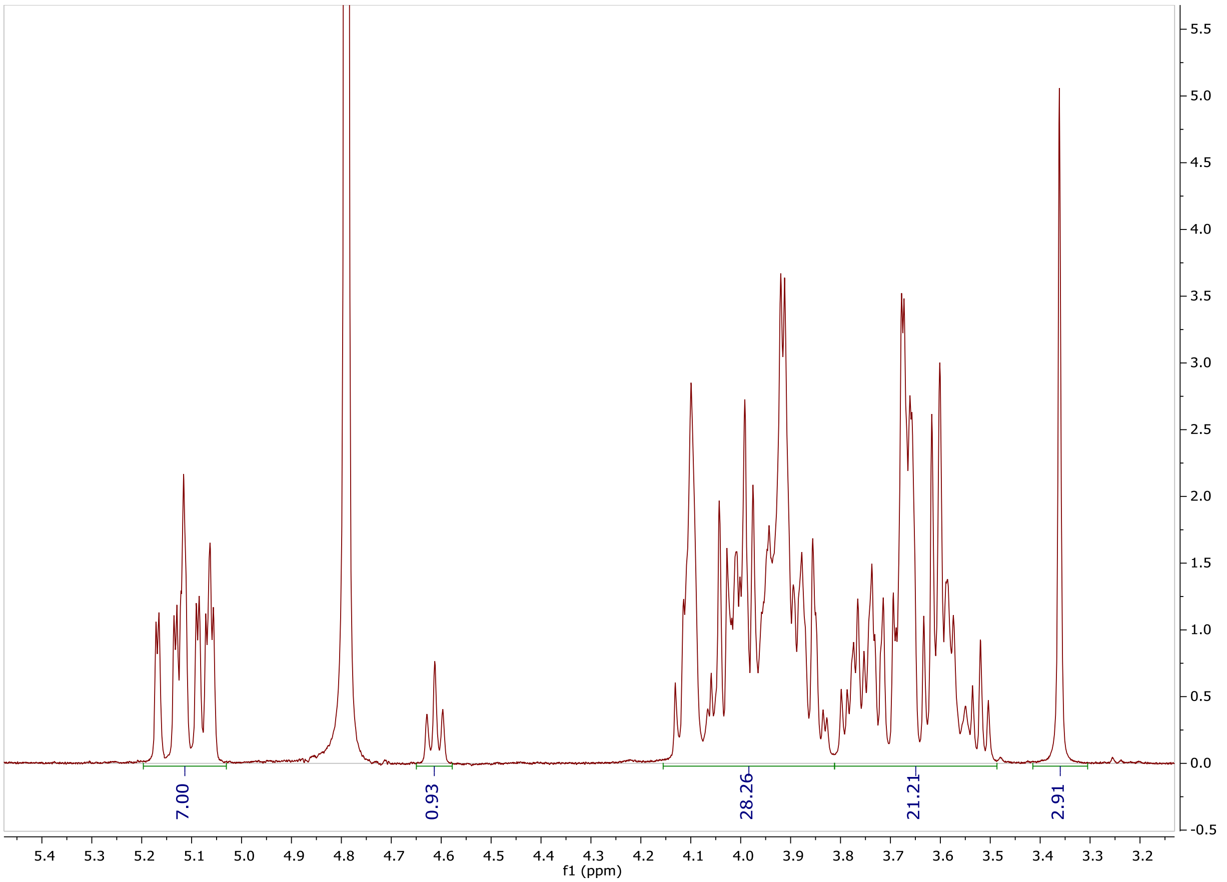

Figure S37. ^{1}H NMR spectrum of MeMO-β-CD with integration (600 MHz, 298 K, D_2O).

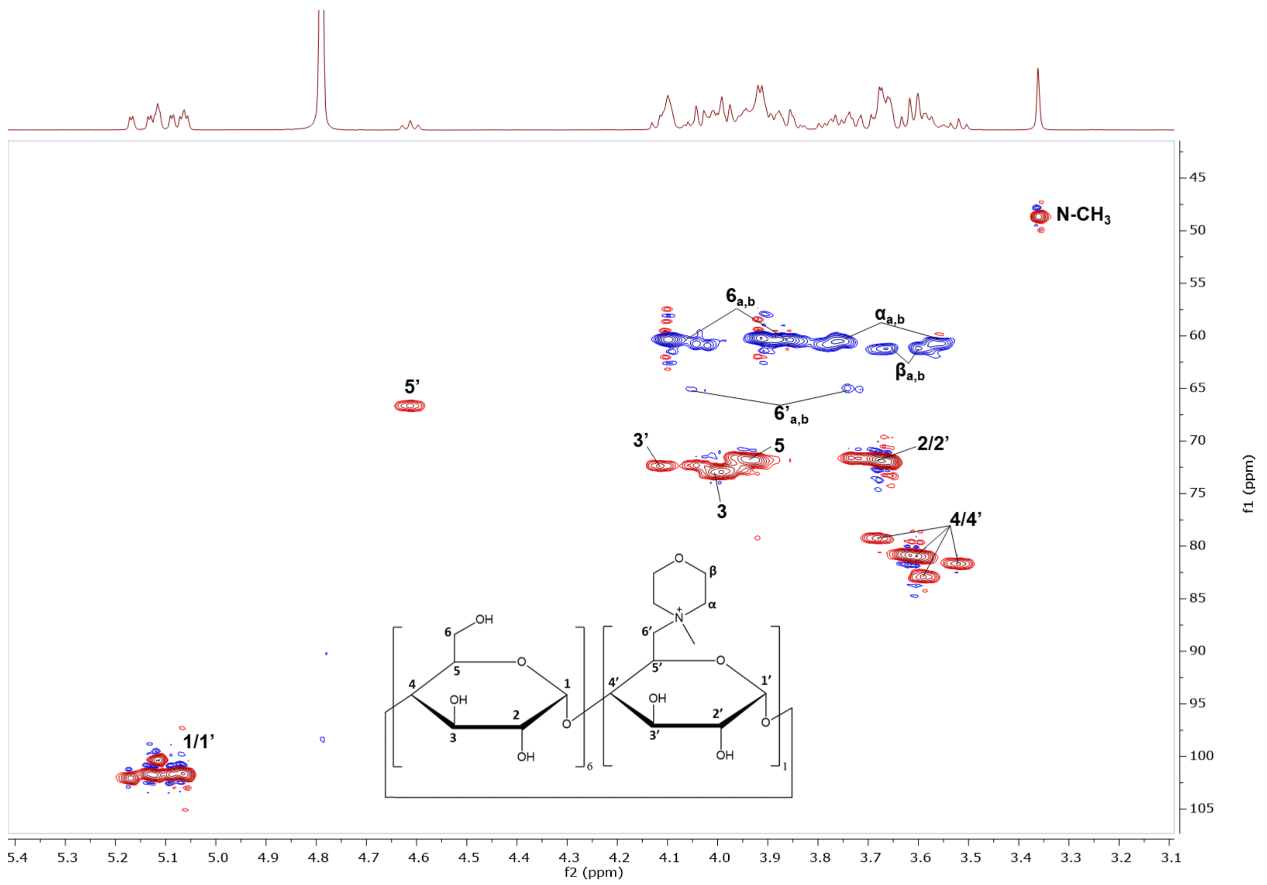

Figure S38. DEPT-edited HSQC spectrum of Me-MO-β-CD with assignment (600 MHz, 298 K, D_2O).

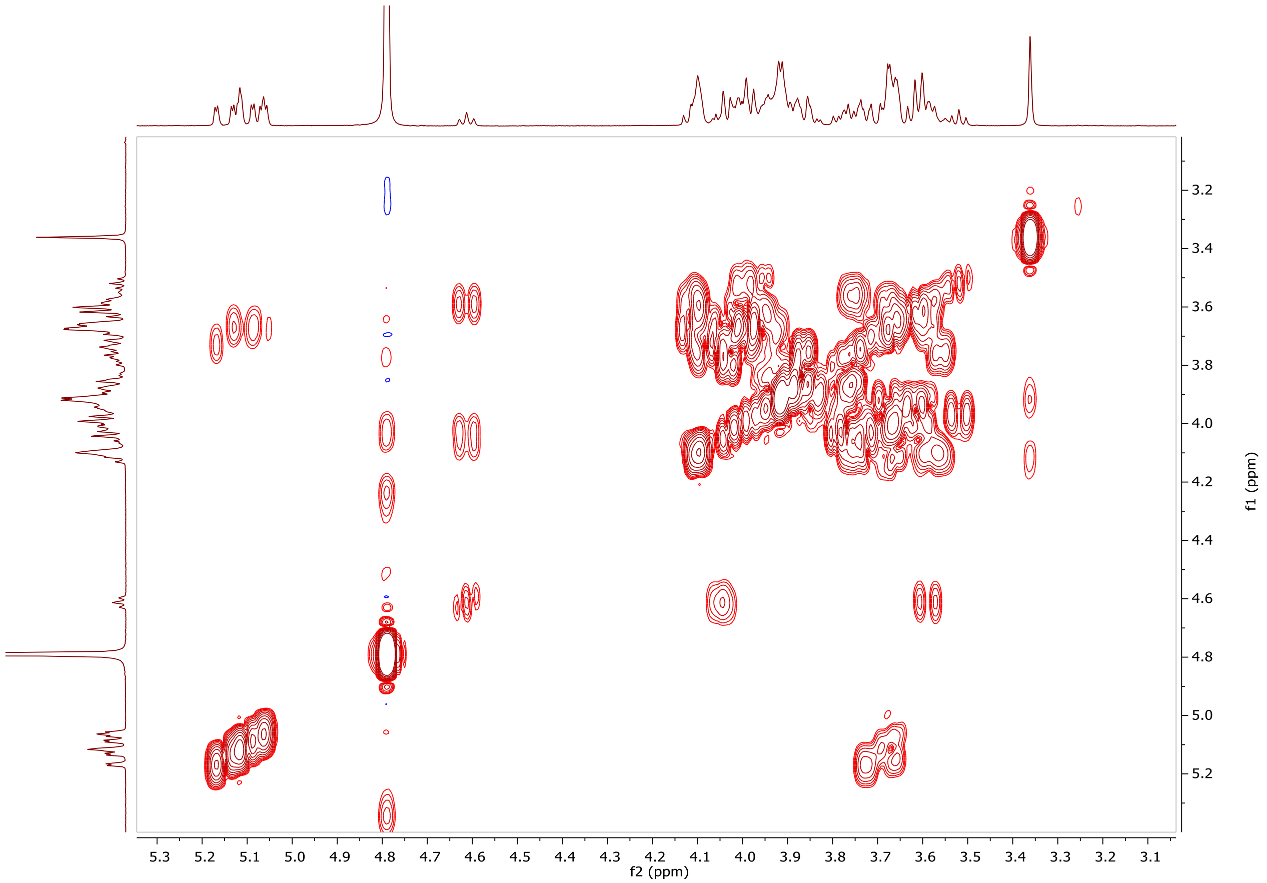

Figure S39. 2D COSY spectrum of Me-MO-β-CD (600 MHz, 298 K, D_2O).

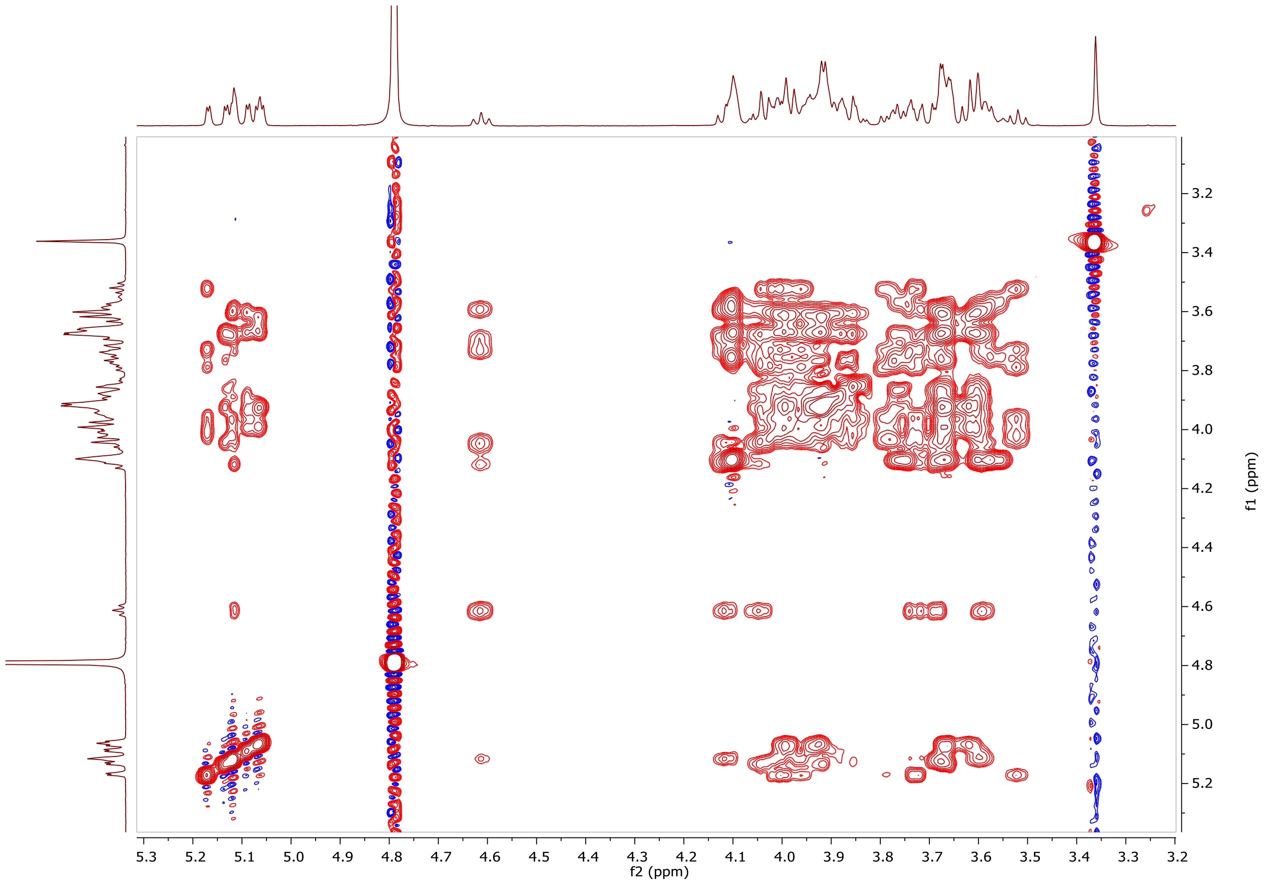

Figure S40. 2D TOCSY spectrum of Me-MO-β-CD (600 MHz, 298 K, D_2O).

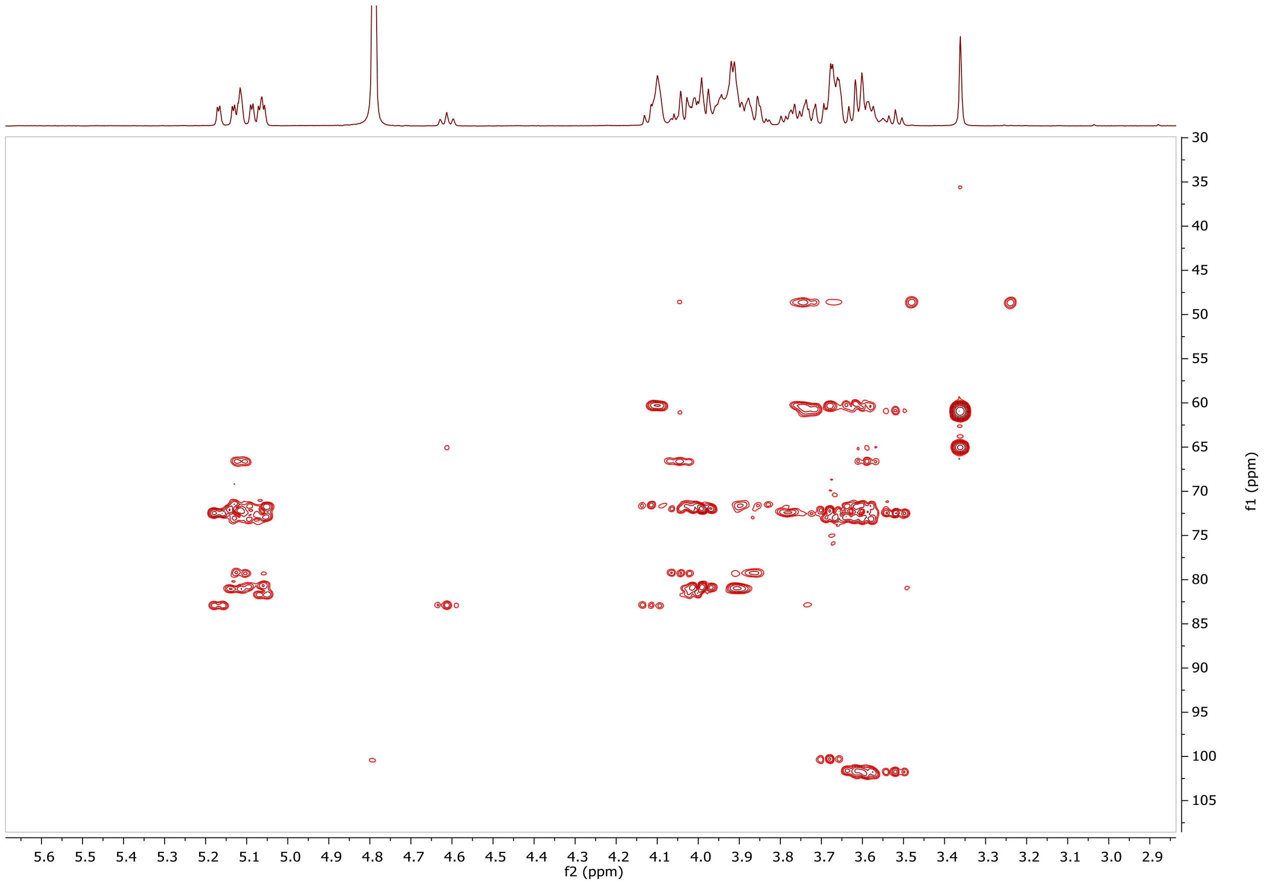

Figure S41. HMBC spectrum of Me-MO-β-CD (600 MHz, 298 K, D_2O).

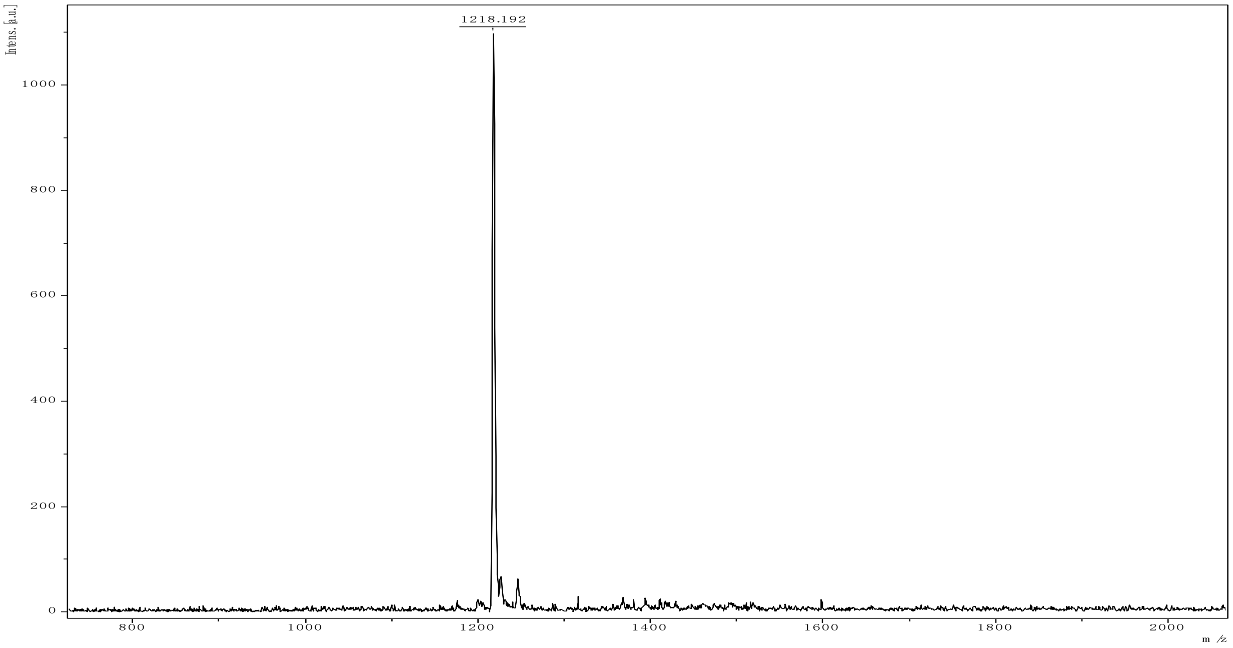

Figure S42. MALDI-TOF-MS spectrum of MeMO-β-CD.

Mono-(6-*N*-(4,4-*N*,*N*-dimethylpiperazine)-β-CD (MePIPA-β-CD)(9)

^{1}H NMR (600 MHz, D_2O): δ (ppm) 5.21-4.98 (m, 7H, H-1), 4.18-3.39 (m, 44H, H-2, H-3, H-4, H-5, H-6), 3.17 (s, 6H, N-CH_3), 3.08-2.79 (m, 8H, H-α, H-β, overlapping with traces of DMF).

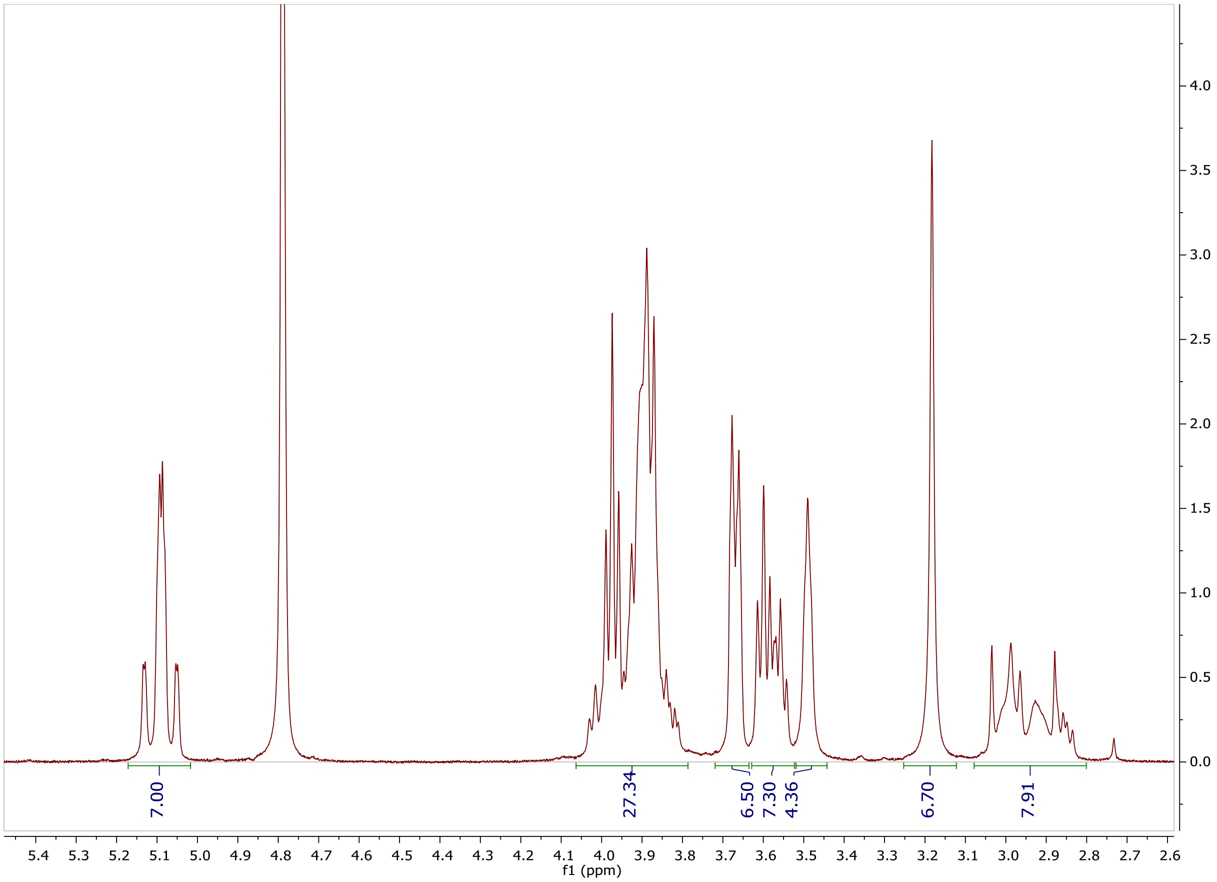

Figure S43. ^{1}H NMR spectrum of MePIPA-β-CD with integration (600 MHz, 298 K, D_2O).

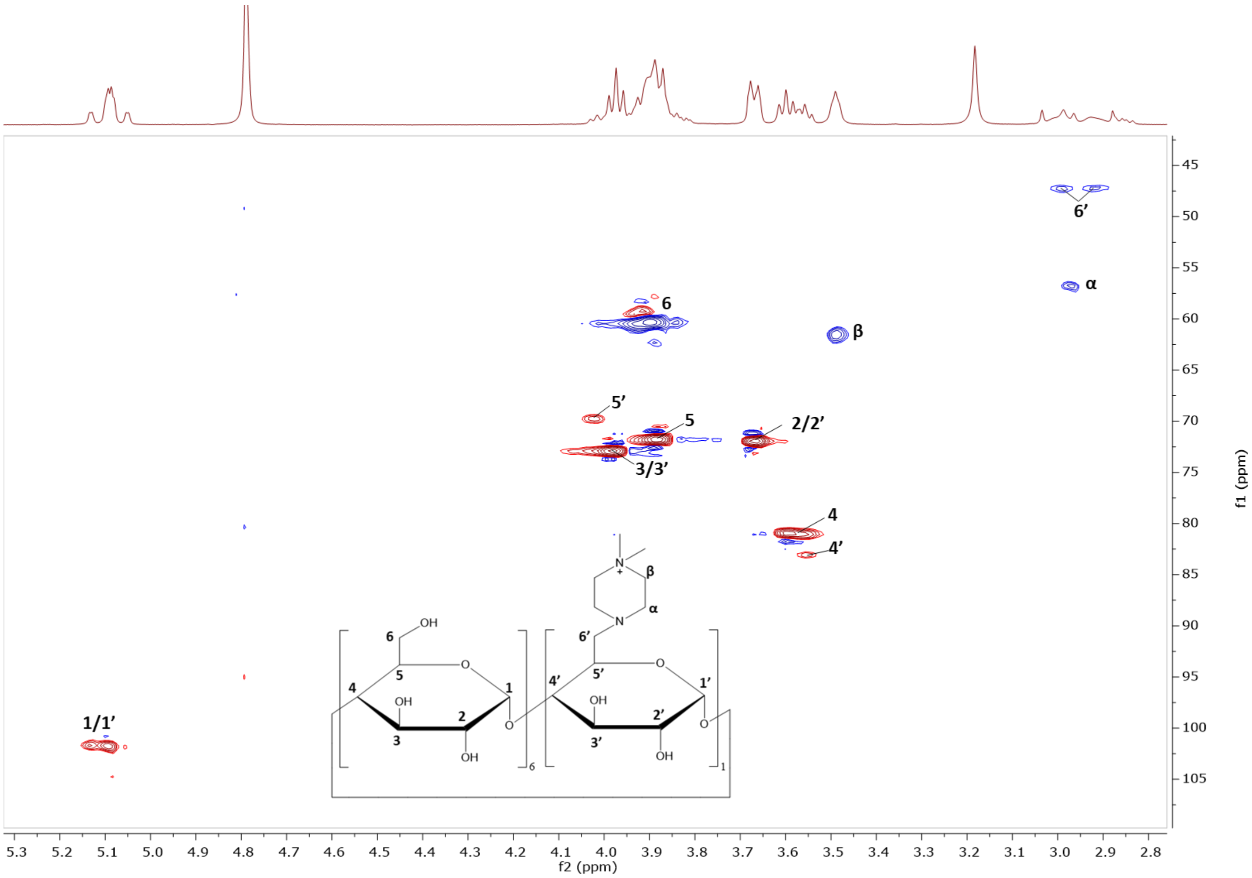

Figure S44. DEPT-edited HSQC spectrum of Me-PIPA-β-CD with assignment (600 MHz, 298 K, D_2O).

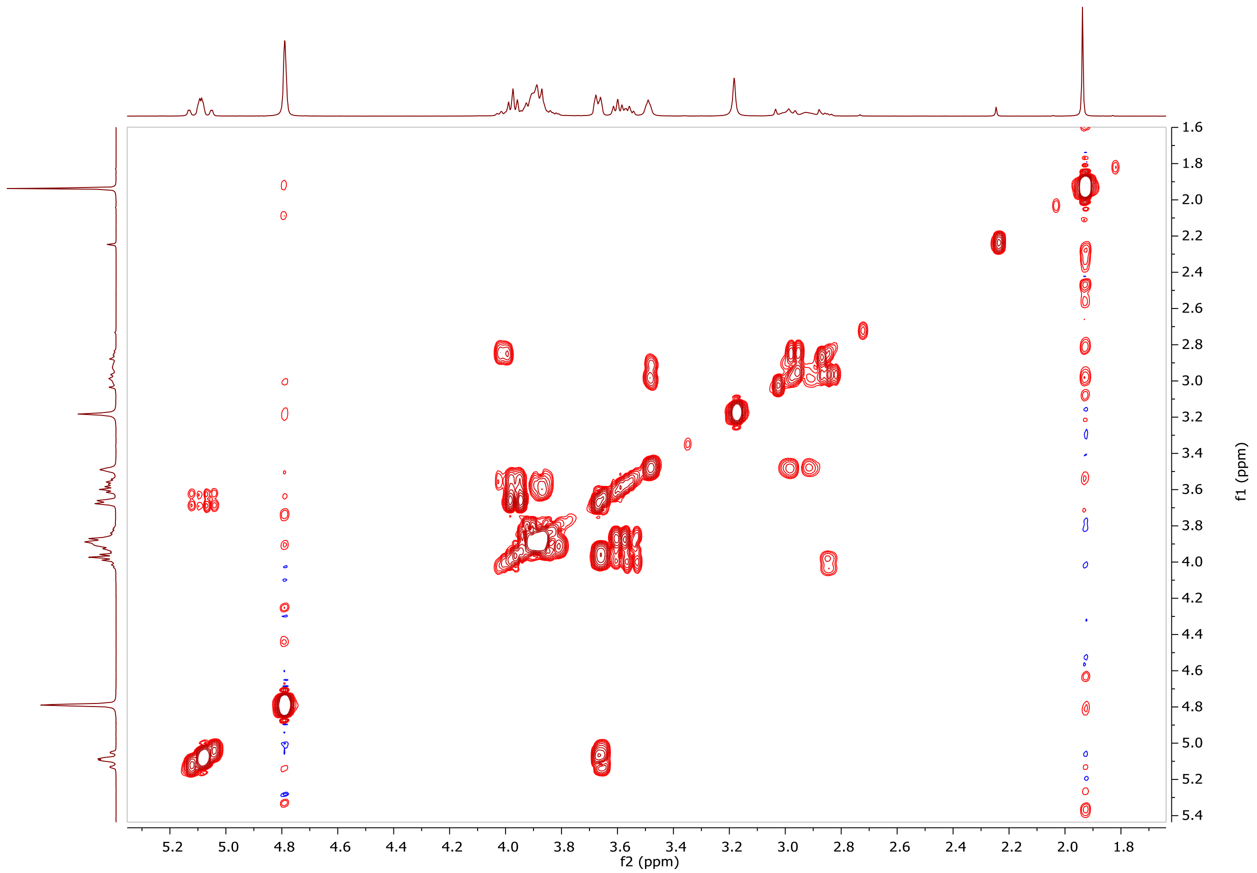

Figure S45. 2D COSY spectrum of Me-PIPA-β-CD (600 MHz, 298 K, D_2O).

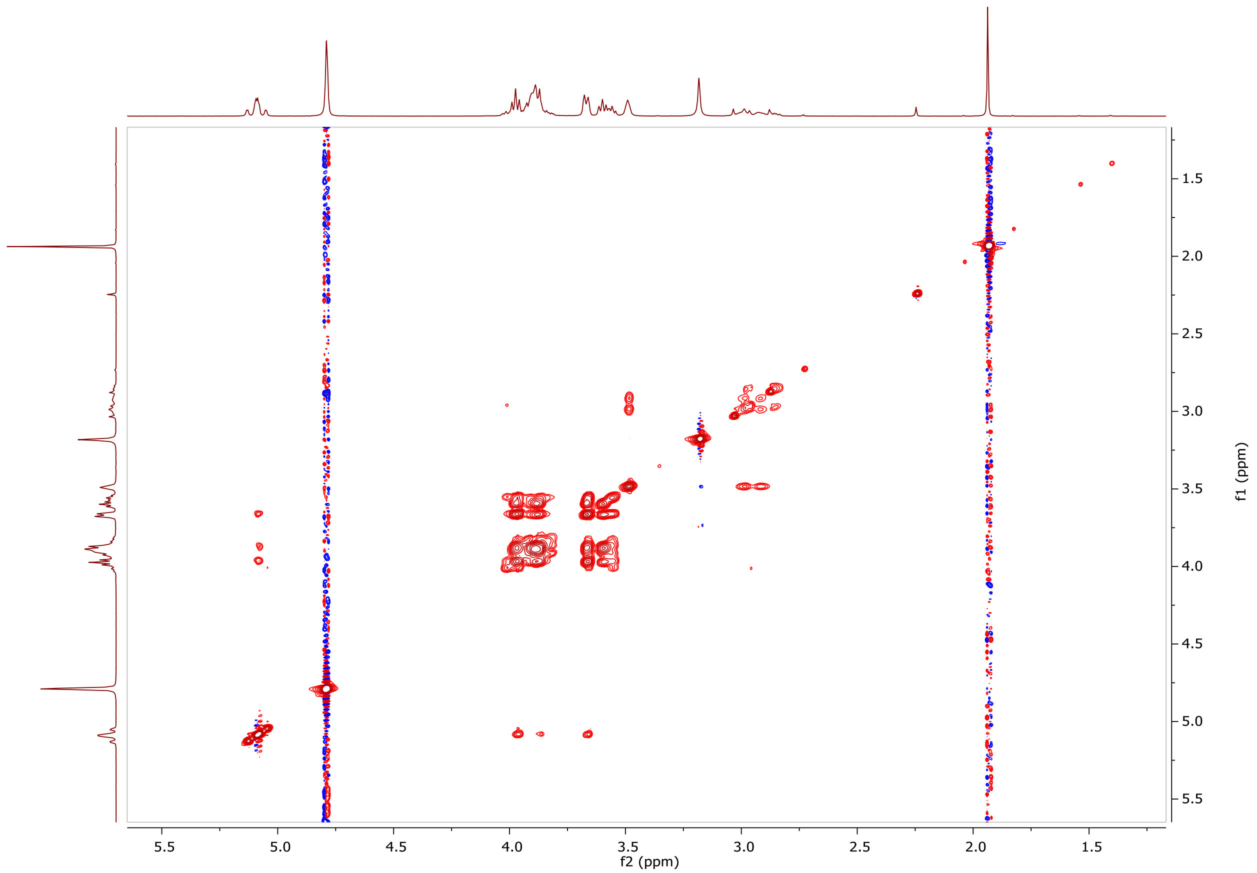

Figure S46. 2D TOCSY spectrum of Me-PIPA-β-CD (600 MHz, 298 K, D_2O).

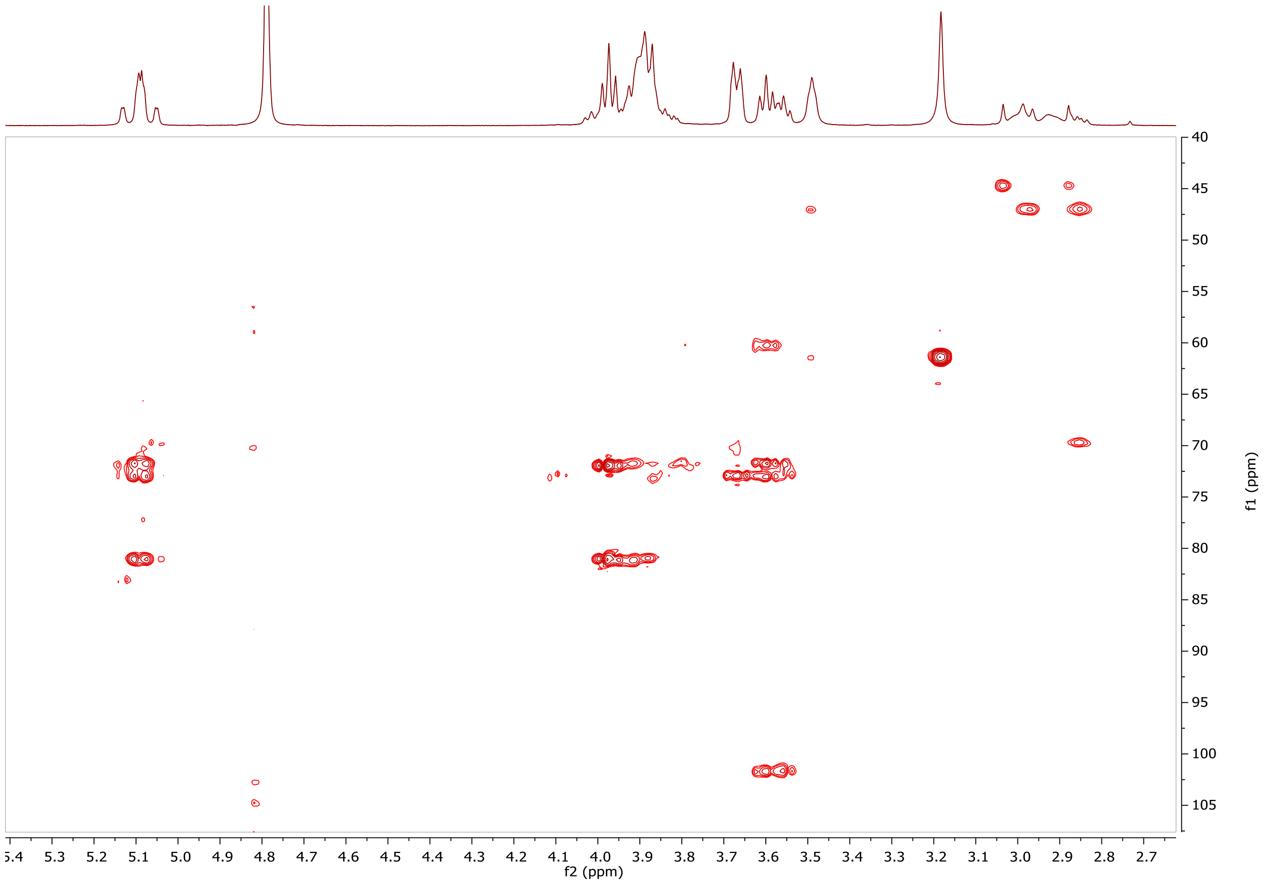

Figure S47. HMBC spectrum of Me-PIPA-β-CD (600 MHz, 298 K, D_2O).

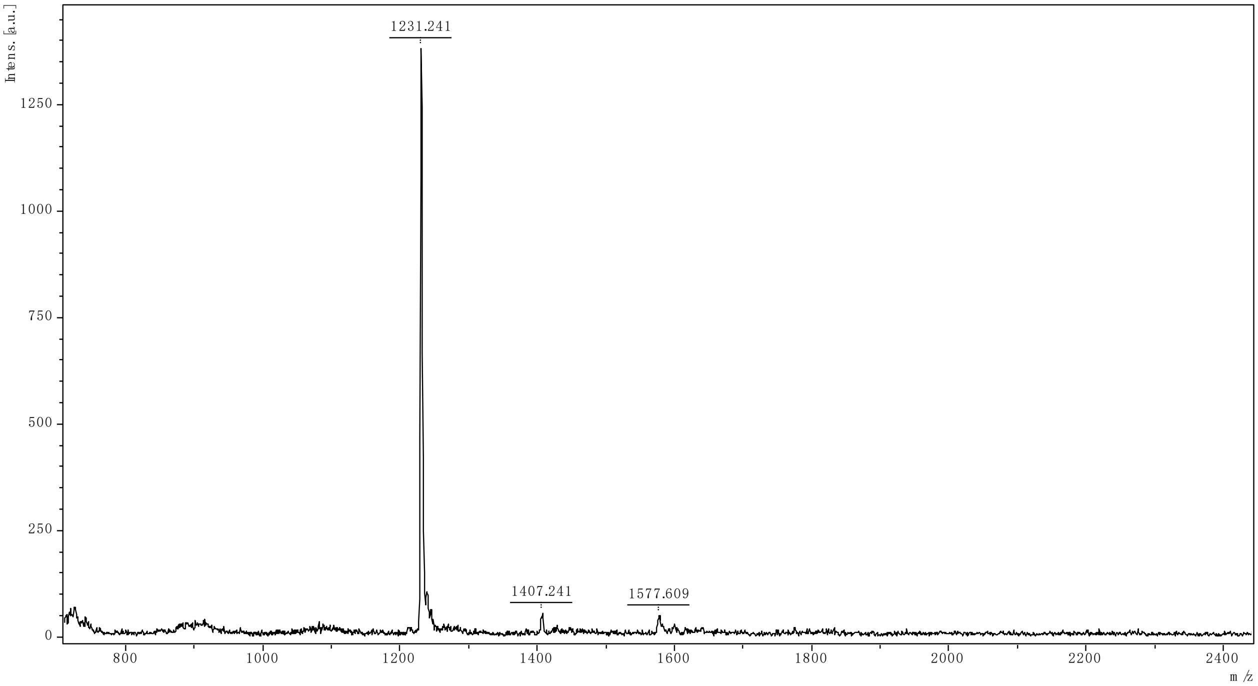

Figure S48. MALDI-TOF-MS spectrum of Me-PIPA-β-CD.

PYR-β-CD (2)

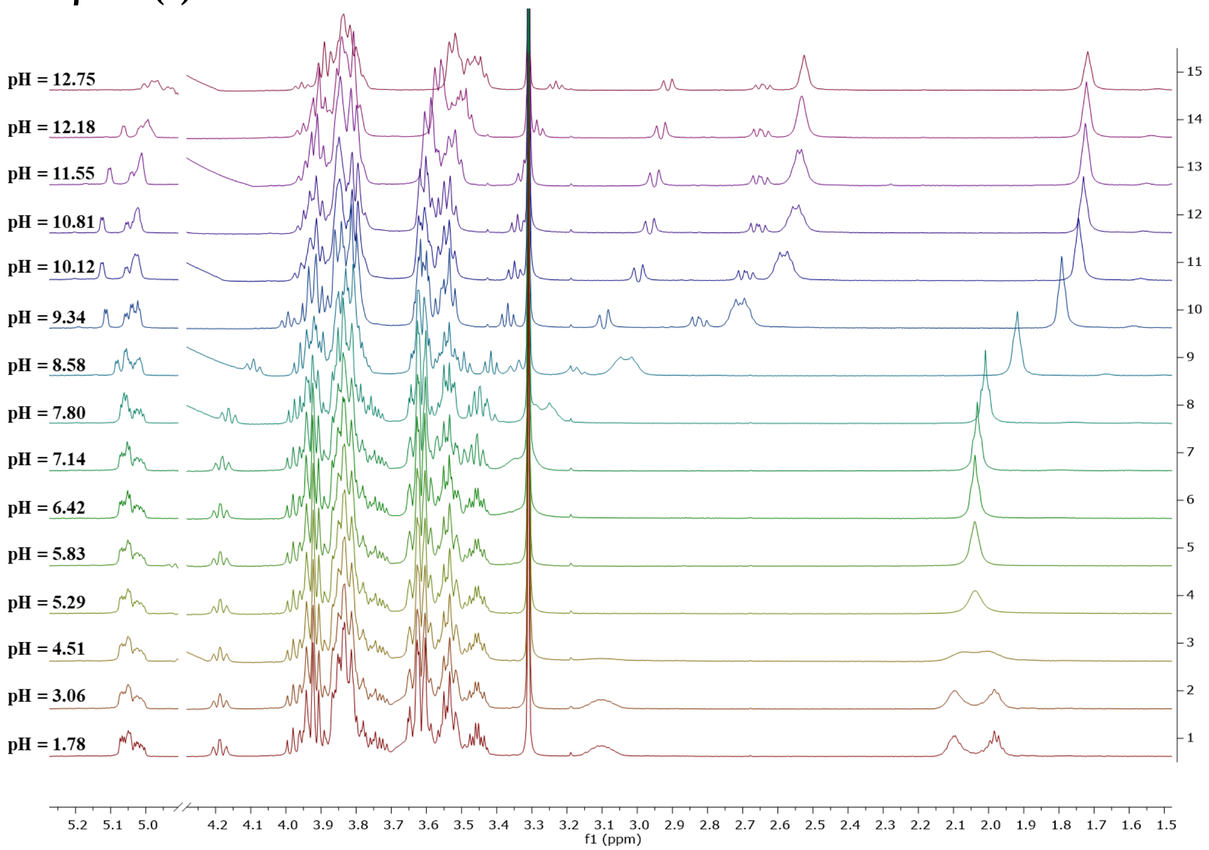

Figure S49. ^{1}H NMR-titration of PYR-β-CD for the determination of the pK_a.

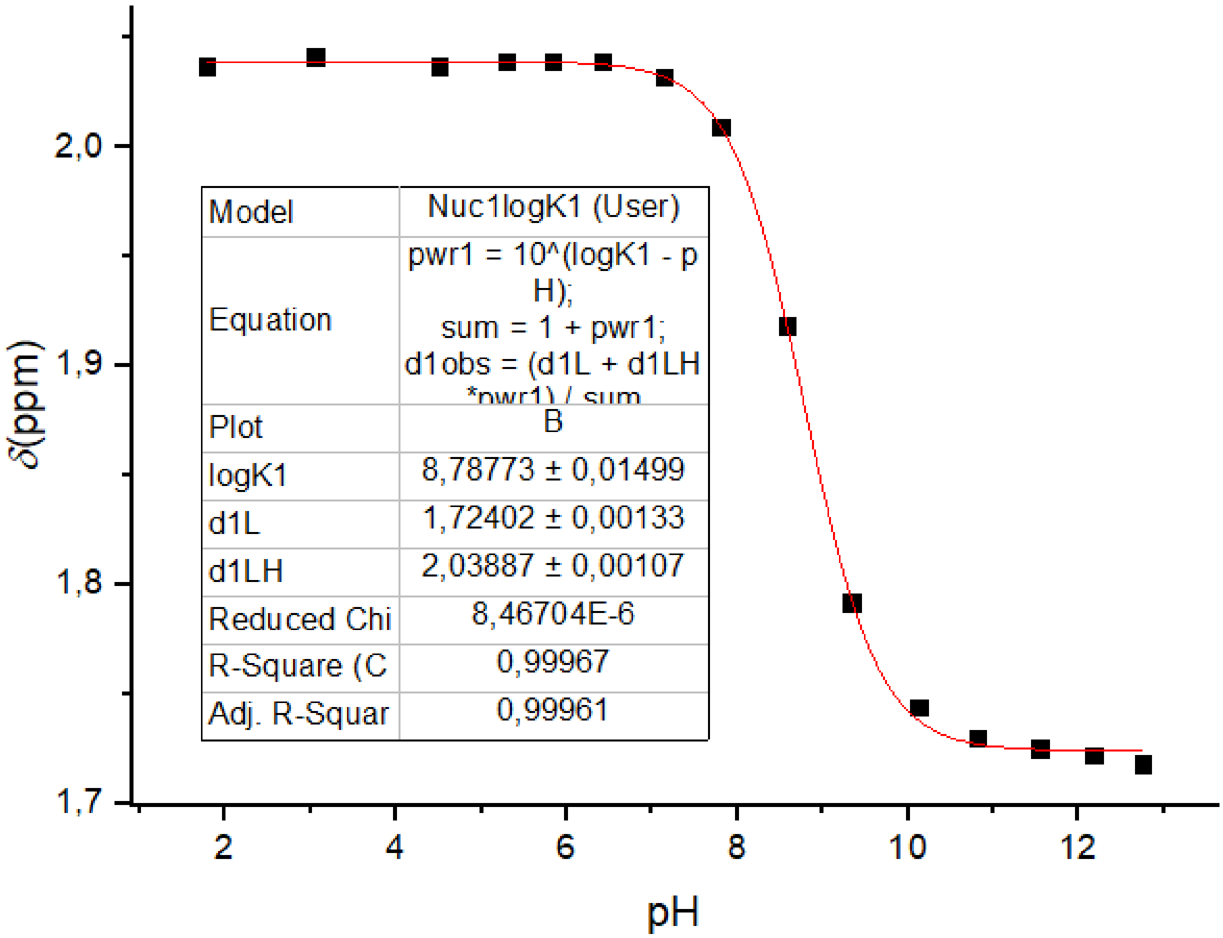

Figure S50. ^{1}H chemical shift of Hβ of PYR-β-CD plotted as a function of pH for the determination of the pK_a.

PIP-β-CD (3)

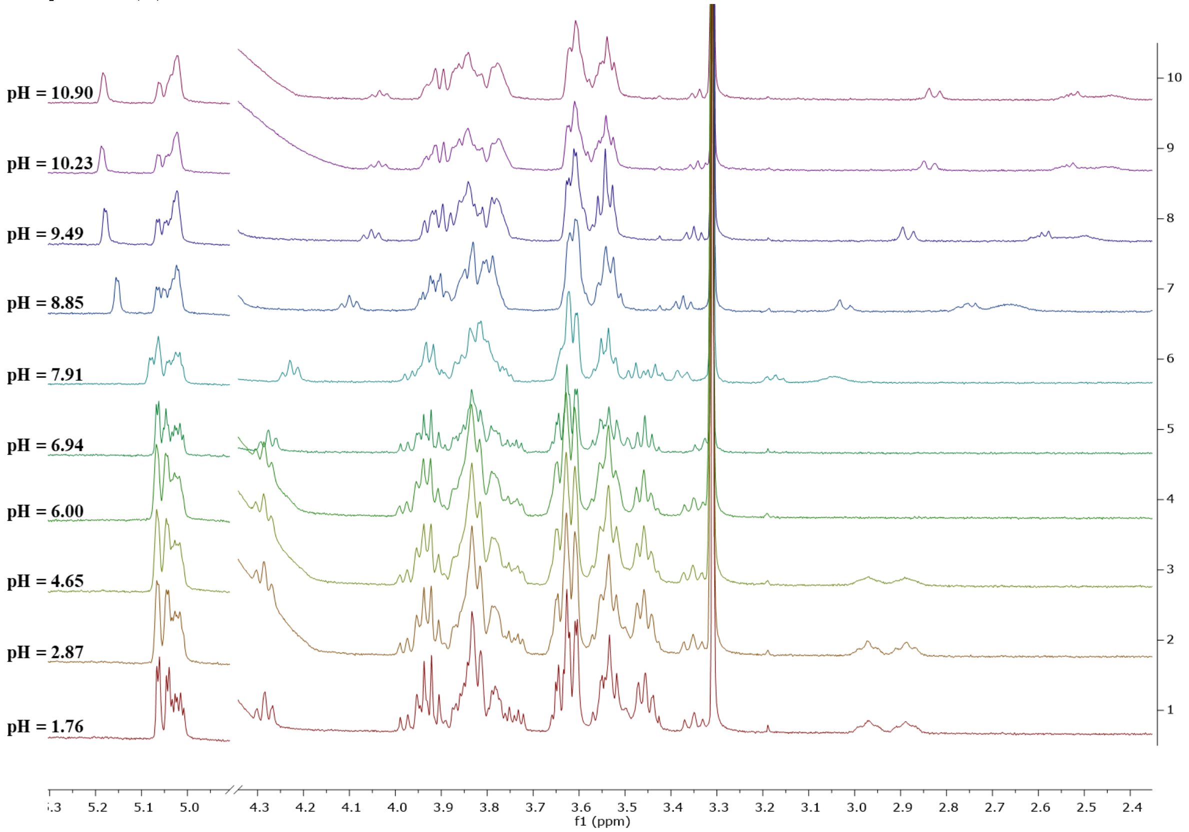

Figure S51. ^{1}H NMR-titration of PIP-β-CD for the determination of the pK_a.

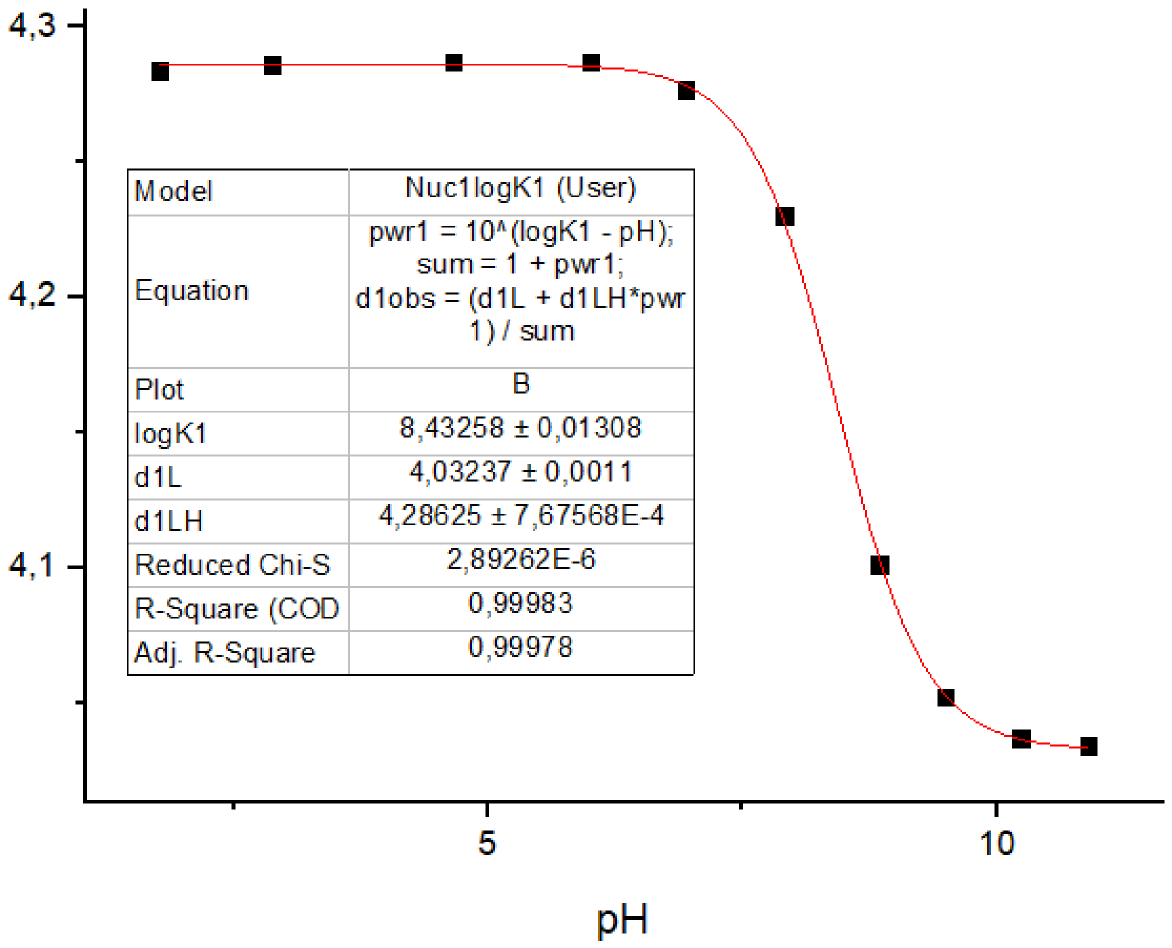

Figure S52. ^{1}H chemical shift of H5' of PIP-β-CD plotted as a function of pH for the determination of the pK_a.

MO-β-CD (4)

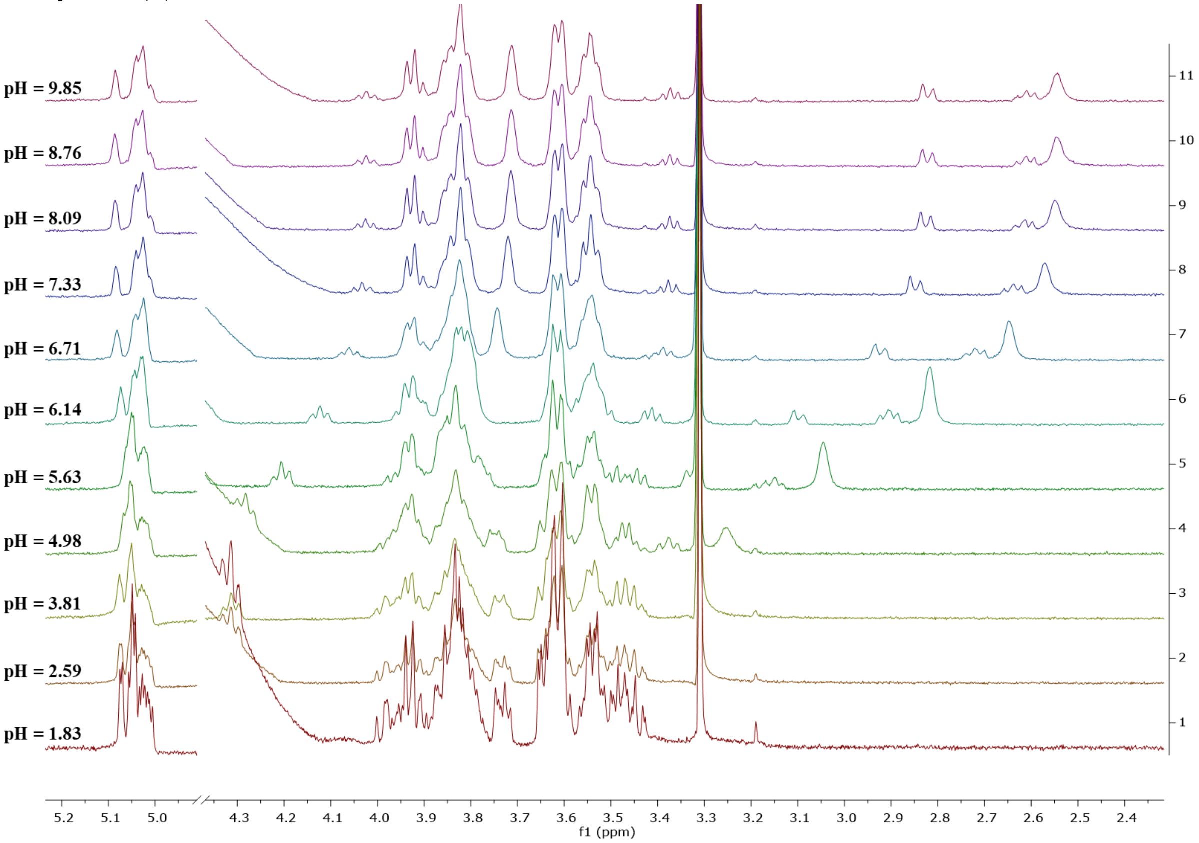

Figure S53. ^{1}H NMR-titration of MO-β-CD for the determination of the pK_a.

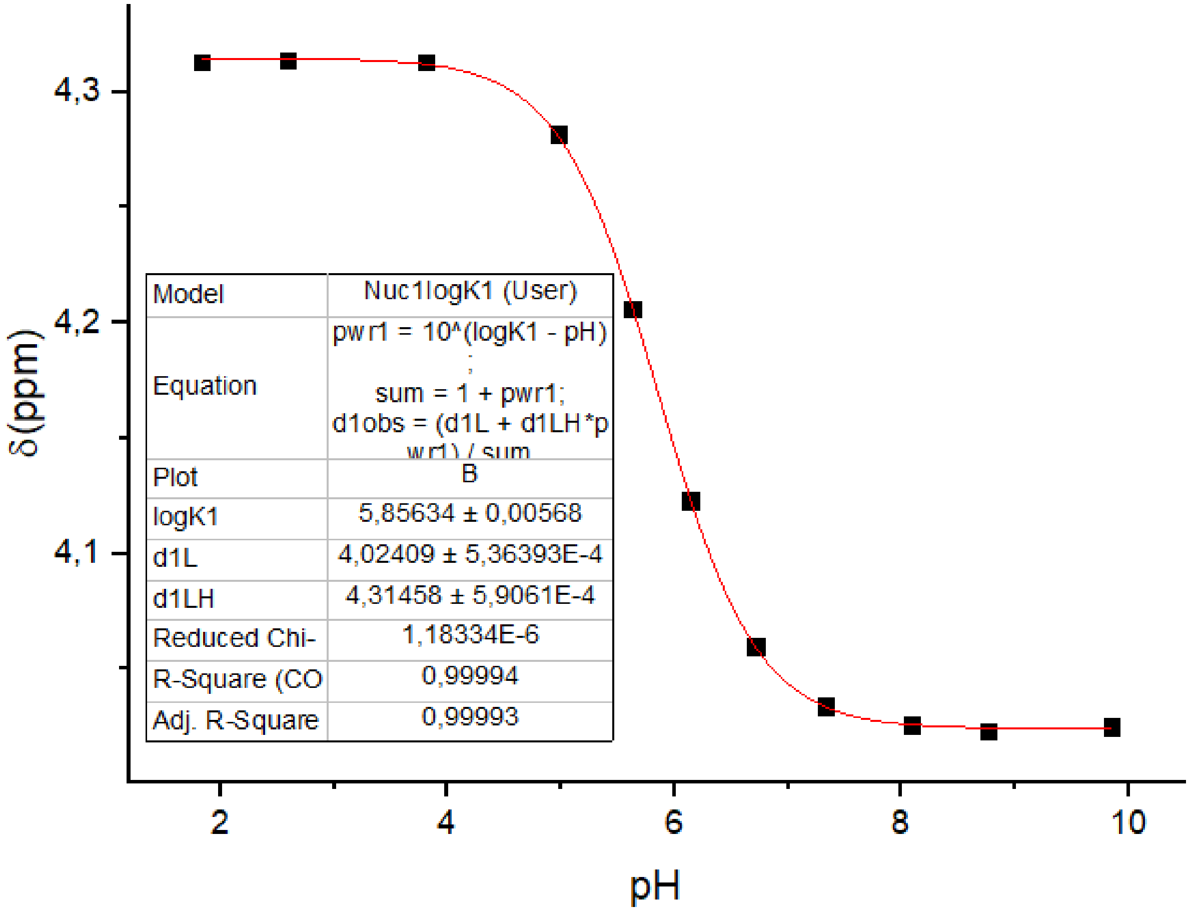

Figure S54. ^{1}H chemical shift of H5' of MO-β-CD plotted as a function of pH for the determination of the pK_a.

PIPA-β-CD (5)

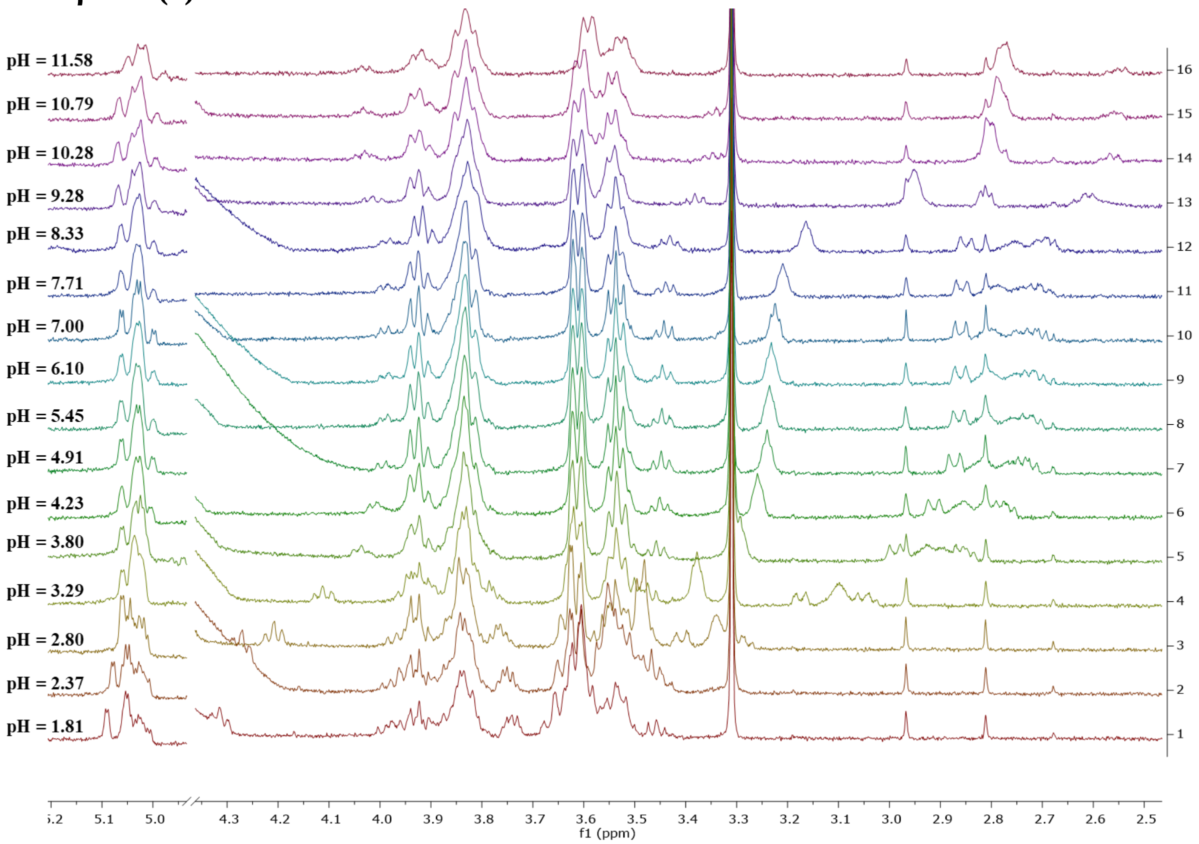

Figure S55. ^{1}H NMR-titration of PIPA-β-CD for the determination of the pK_a.

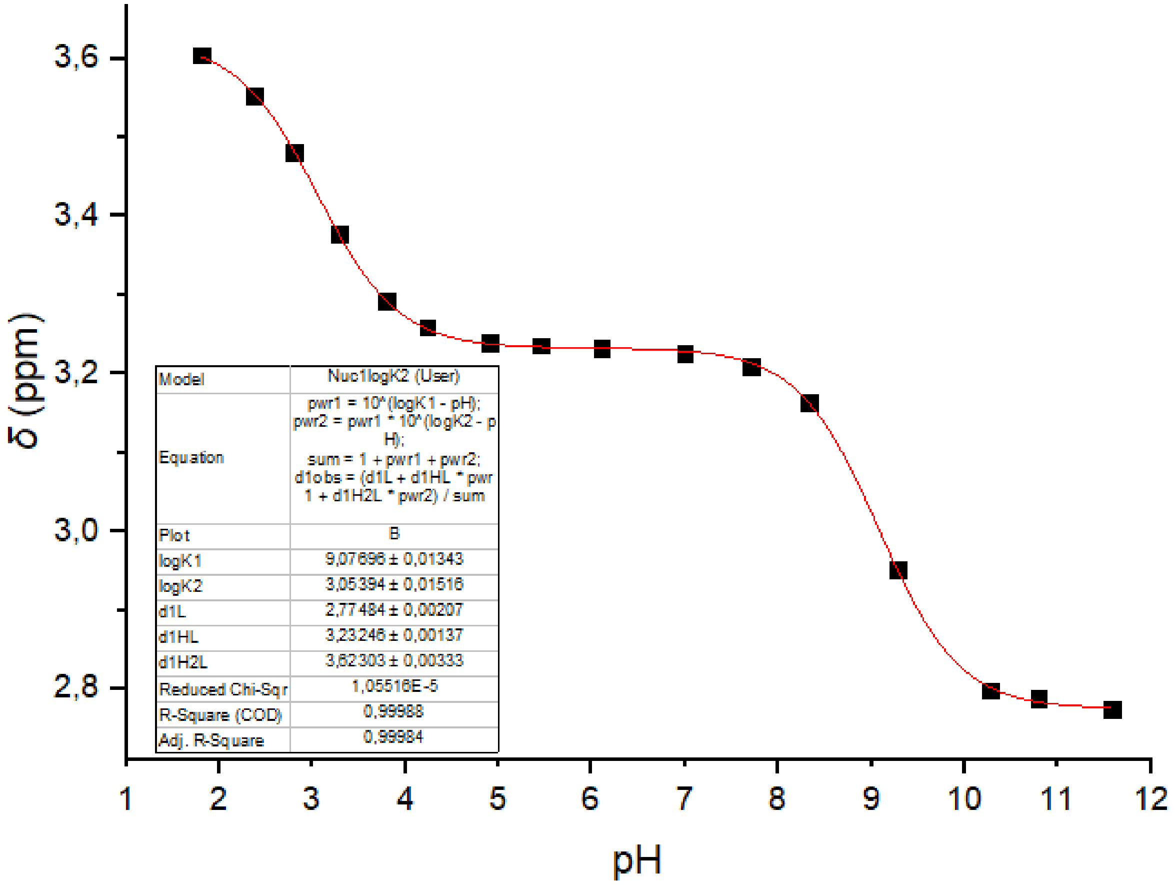

Figure S56. ^{1}H chemical shift of H5’ of PIPA-β-CD plotted as a function of pH for the determination of the pK_a.

2. Enantioseparation performance of the ionizable *N*-heterocyclic CDs at pH 10.0 and at pH 4.75

Table S1. Enantioseparation of the tested model racemates with ionizable *N*-heterocyclic CDs (the first set of CD derivatives) at pH 10.0 and at pH 4.75, indicating the maximum resolution values (*Rs*).

Legend: -: no separation, n.a .: electropherogram could not be evaluated (due to peak distortion caused by the proximity of the EOF)

	**pH 10.0**				**pH 4.75**
	**PYR-β-CD**	**PIP-β-CD**	**MO-β-CD**	**PIPA-β-CD**	**MO-β-CD**
	**0.5 mM**	**0.5 mM**	**0.5 mM**	**0.5 mM**	**2.5 mM**
**tPA**	-	-	-	0.40	-
**2-PPA**	-	-	-	-	**5.41**
**Chlorprop**	-	-	-	-	**6.47**
**Mecoprop**	-	-	-	-	1.49
**Trichlorprop**	-	-	-	0.27	**3.14**
**Dns-Ser**	0.33	-	0.41	0.41	-
**Dns-Thr**	-	-	-	0.18	-
**Dns-Val**	-	-	-	-	**5.31**
**Cetirizine**	-	-	-	0.55	-
**Fexofenadine**	-	0.33	-	0.05	-

CE conditions: 48.5/40 cm, 50 µm id capillary, 0.5-5 mM CD in 20 mM $Na_2B_4O_7$-NaOH pH 10.0 or 20 mM acetic acid-Tris pH 4.75 buffer, 25 kV; 25 °C; 200 nm.

3. Enantioseparation performance of the native- and the acyclic derivatives

Table S2. Enantioseparation of the tested model racemates with native β-CD, mono-6-amino-β-CD (A-β-CD), mono-6-*N*,*N*-dimethyl-amino-β-CD (DMA-β-CD), and mono-6-*N*,*N*,*N*-trimethyl-amino-β-CD (TMA-β-CD), at **pH 6.0**, indicating the maximum resolution values (*Rs*).

Legend: -: no separation, n.a .: electropherogram could not be evaluated (due to peak distortion caused by the proximity of the EOF)

	**pH 6.0**														
	**β-CD**			**A-β-CD**				**DMA-β-CD**				**TMA-β-CD**			
	**1 mM**	**2.5 mM**	**5 mM**	**0.5 mM**	**1 mM**	**2.5 mM**	**5 mM**	**0.5 mM**	**1 mM**	**2.5 mM**	**5 mM**	**0.5 mM**	**1 mM**	**2.5 mM**	**5 mM**
**MA**	-	-	-	-	-	-	-	-	0.93	n.a.	n.a.	-	-	-	-
**TA**	-	-	-	-	-	-	-	-	0.09	n.a.	n.a.	-	-	-	-
**cCA**	-	-	-	1.01	1.20	**1.80**	**1.56**	1.37	1.27	**2.53**	n.a.	-	-	0.55	0.66
**tPA**	-	-	-	0.79	0.85	**1.62**	1.23	1.36	0.84	n.a.	n.a.	-	-	-	-
**2-PPA**	-	0.26	0.22	0.26	0.57	0.73	0.92	-	n.a.	n.a.	n.a.	-	-	-	-
**Chlorprop**	-	0.63	0.38	0.53	0.48	**2.41**	**2.23**	0.35	0.39	0.78	0.93	-	-	-	-
**Mecoprop**	-	-	-	-	-	0.21	0.52	-	-	-	-	-	-	0.47	0.63
**Dichlorprop**	-	-	0.22	-	-	-	-	-	-	-	-	-	-	-	-
**Trichlorprop**	-	-	0.84	0.56	n.a.	n.a.	-	-	-	-	-	-	-	-	-
**Dns-Ser**	-	0.34	0.50	-	-	-	-	-	0.51	0.52	0.67	-	0.57	0.79	1.06
**Dns-Thr**	-	0.12	0.38	-	-	-	-	-	-	-	-	-	-	-	-
**Dns-Val**	-	0.31	0.51	-	-	-	-	-	-	-	-	-	-	-	-
**Flurbiprofen**	-	-	-	-	-	-	0.78	-	-	-	-	-	-	-	-
**Ibuprofen**	-	-	-	-	-	0.79	-	-	n.a.	n.a.	n.a.	-	-	-	-
**Gatifloxacin**	-	-	-	0.63	-	-	-	0.30	-	-	-	-	-	-	-

CE conditions: 48.5/40 cm, 50 µm id capillary, 0.5-5 mM CD in 20 mM NaH_2PO_4-NaOH pH 6.0 buffer, 25 kV; 25 °C; 200 nm.

Representative electropherograms of the enantioseparation of Dns-Val

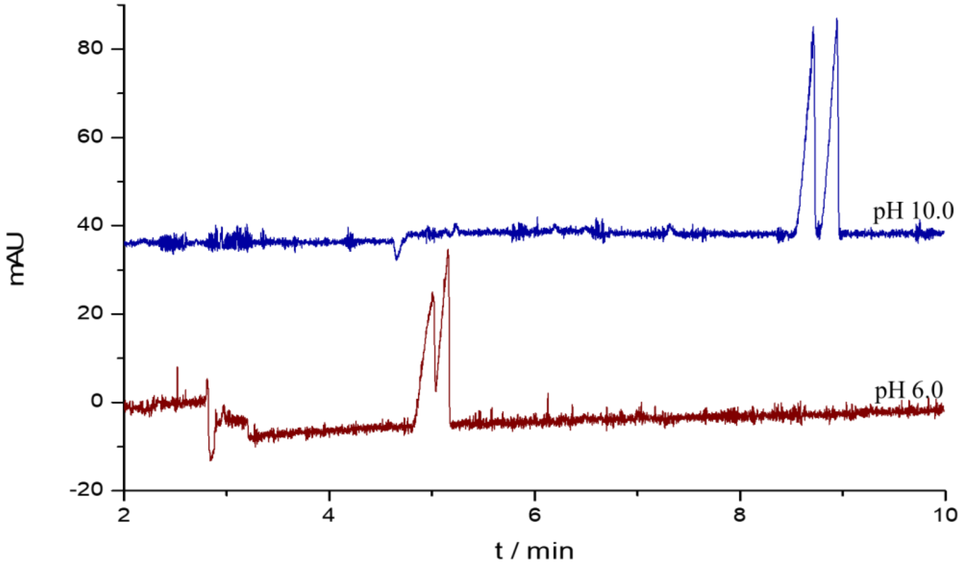

Figure S57. Representative electropherograms of the enantioseparation of Dns-Val demonstrating the effect of the pH on the enantioresolution with 5 mM MePYR-β-CD

4. Enantiomer migration orders, complex mobilities and apparent complex stability constants

Effective mobility values were calculated as:

$$\mu_{eff} = \frac{l_c l_t}{U} * \left(\frac{1}{t} - \frac{1}{t_{EOF}}\right) \quad (1)$$

where l_t is the total length of the capillary, l_c is the length of the capillary to the detector, U is the applied voltage, while t and t_{EOF} are the peak appearance times of the analyte and the EOF marker, respectively.

To obtain the CD-Dns-Val binding constants, the experimental μ_{eff} versus c_{CD} dataset was fitted by the following function in Microcal Origin (OriginLabs),

$$\mu_{eff} = \frac{\mu_{free} + \mu_{cplx}\ K[CD]}{1 + K[CD]} \quad (2)$$

where μ_{free} is the mobility of Dns-Val in the absence of CD, μ_{cplx} and K are the electrophoretic mobility and the binding constant of the CD-Dns-Val complex, respectively.

Table S3. The enantiomer migration order, the complex mobilities and the apparent complex stability constants of the first and second migrating **Dns-Val** enantiomers at pH 6.0 phosphate buffer

	**PYR-β-CD**		**PIP-β-CD**		**MO-β-CD**		**PIPA-β-CD**	
**EMO**	***D, L***		***L, D***		***D, L***		***D, L***	
$\mu_{Complex1}$ (10^{-5} $cm^2V^{-1}s^{-1}$)	2.8	±1.1	-3.5	±1.9	-9.1	±2.8	-1.1	±0.8
$\mu_{Complex2}$ (10^{-5} $cm^2V^{-1}s^{-1}$)	5.8	±1.3	-5.2	±1.2	-12.2	±0.2	-0.8	±0.9
K_{stab1}	**154**	±13	**398**	±96	**261**	±122	**237**	±21
K_{stab2}	**109**	±8	**471**	±89	**929**	±77	**198**	±18

5. NMR study of the complex formation

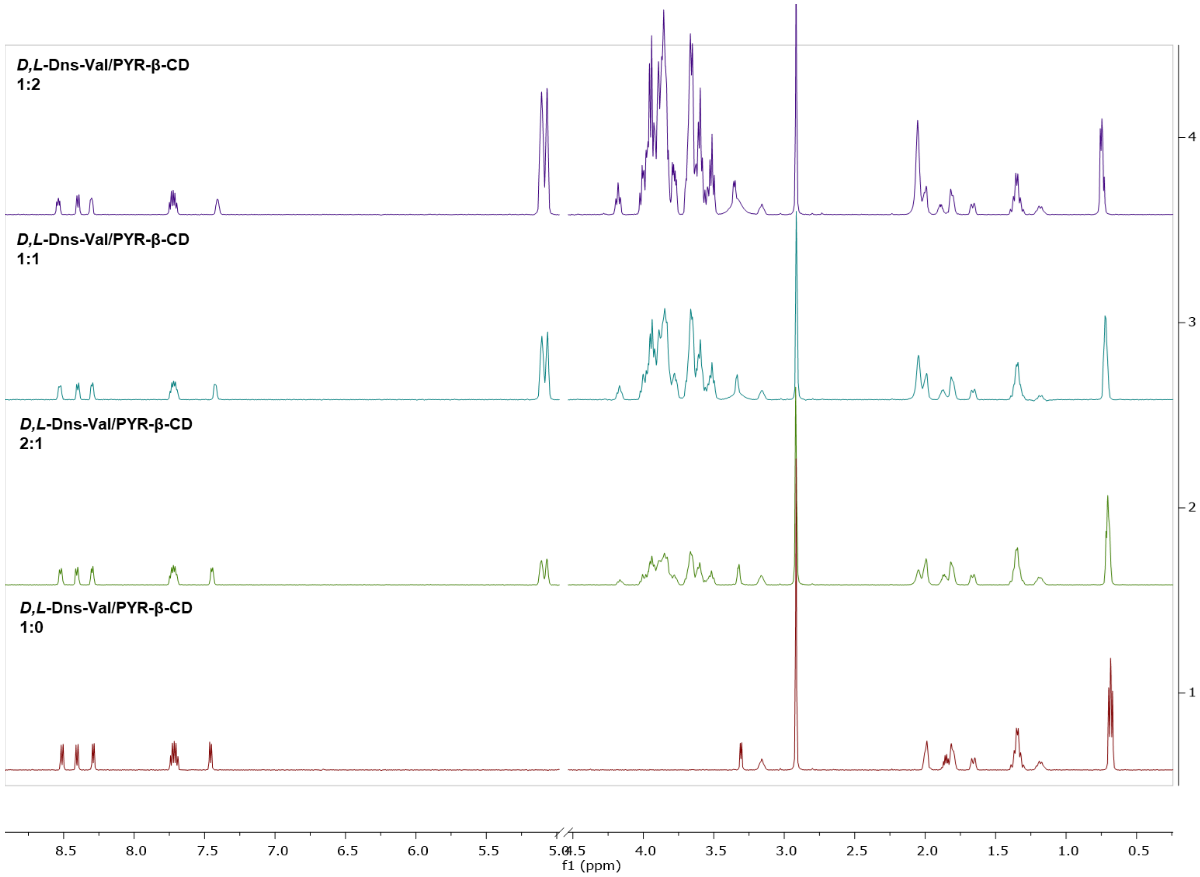

Figure S58. Stacked ^{1}H NMR spectra of *D,L*-Dns-Val/PYR-β-CD system in 20 mM NaH_2PO_4 buffer at pH* 6.0 with increasing PYR-β-CD concentration (from bottom to top) (600 MHz, 298 K).

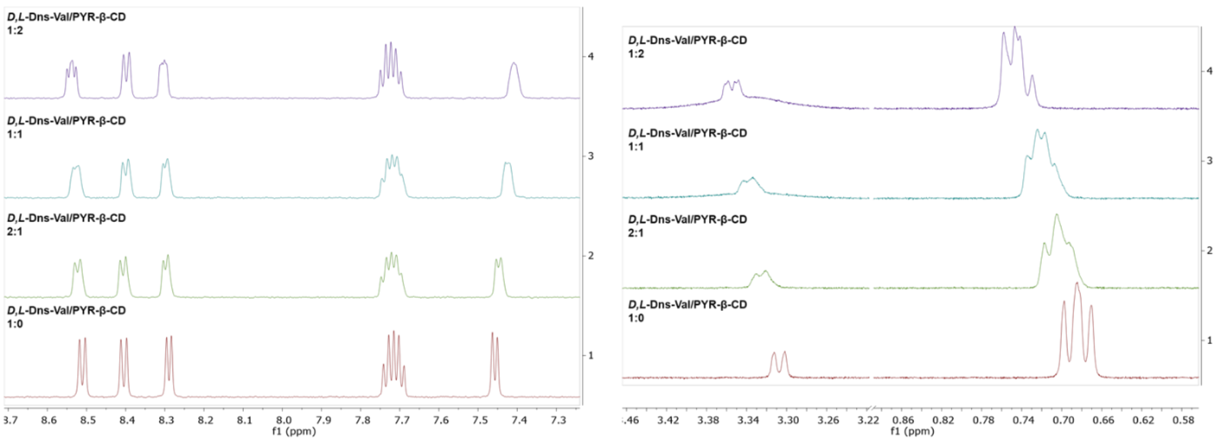

Figure S59. Stacked partial ^{1}H NMR spectra of *D,L*-Dns-Val/PYR-β-CD system in 20 mM NaH_2PO_4 buffer at pH* 6.0 with increasing PYR-β-CD concentration (from bottom to top) (600 MHz, 298 K).

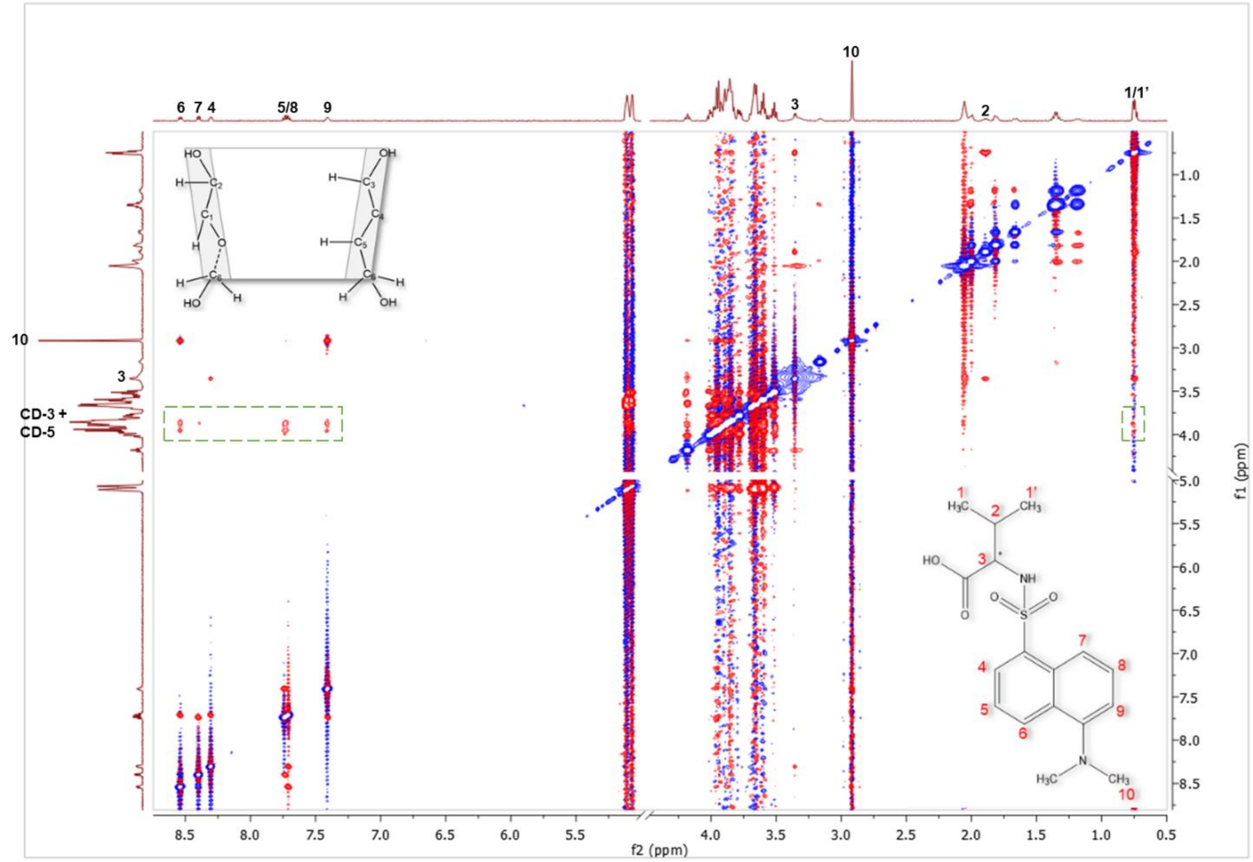

Figure S60. 2D ROESY spectrum of *D*,*L*-Dns-Val/PYR-β-CD system with intermolecular correlations emphasized and structure of the compounds (600 MHz, 298 K, D_2O).

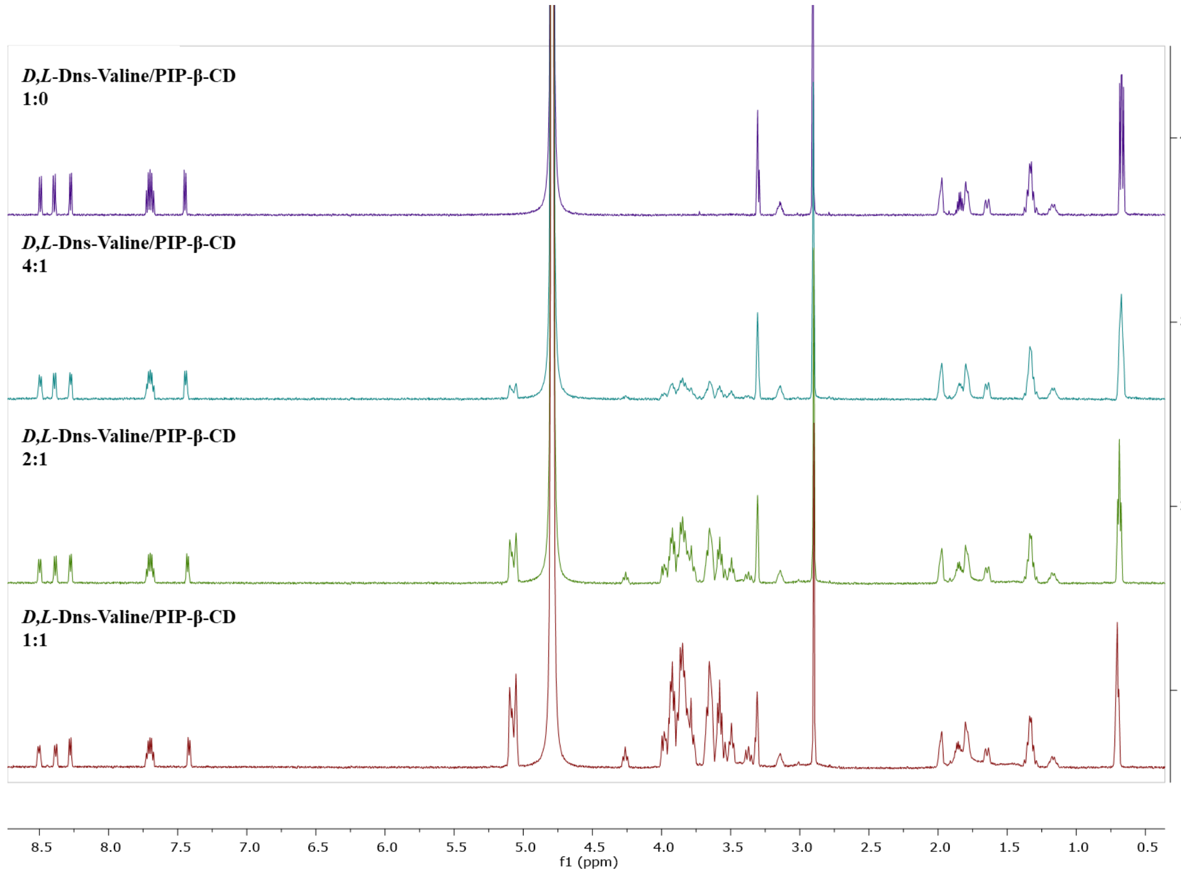

Figure S61. Stacked ^{1}H NMR spectra of *D,L*-Dns-Val/PIP-β-CD system in 20 mM NaH_2PO_4 buffer at pH* 6.0 with increasing PIP-β-CD concentration (from top to bottom) (600 MHz, 298 K).

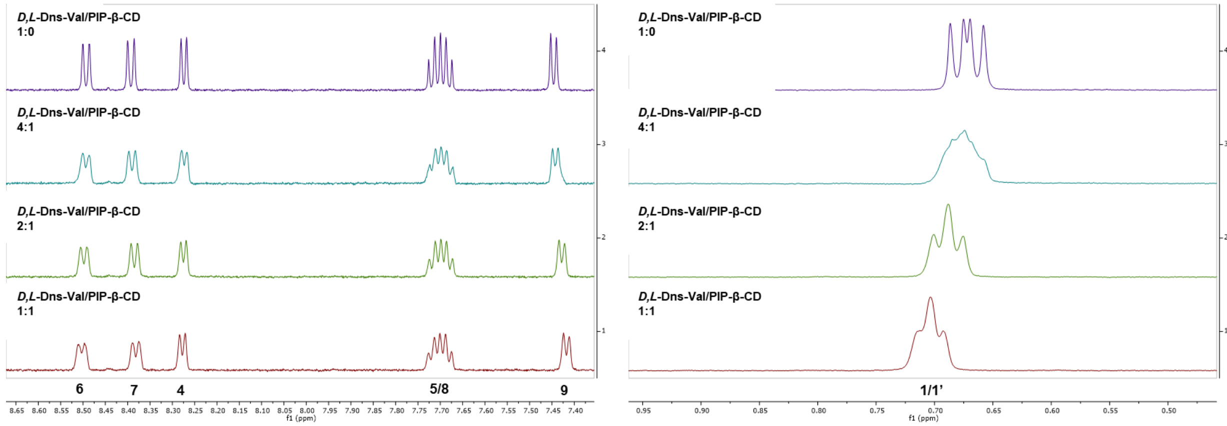

Figure S62. Stacked partial ^{1}H NMR spectra of *D,L*-Dns-Val/PIP-β-CD system in 20 mM NaH_2PO_4 buffer at pH* 6.0 with increasing PIP-β-CD concentration (from top to bottom) (600 MHz, 298 K).

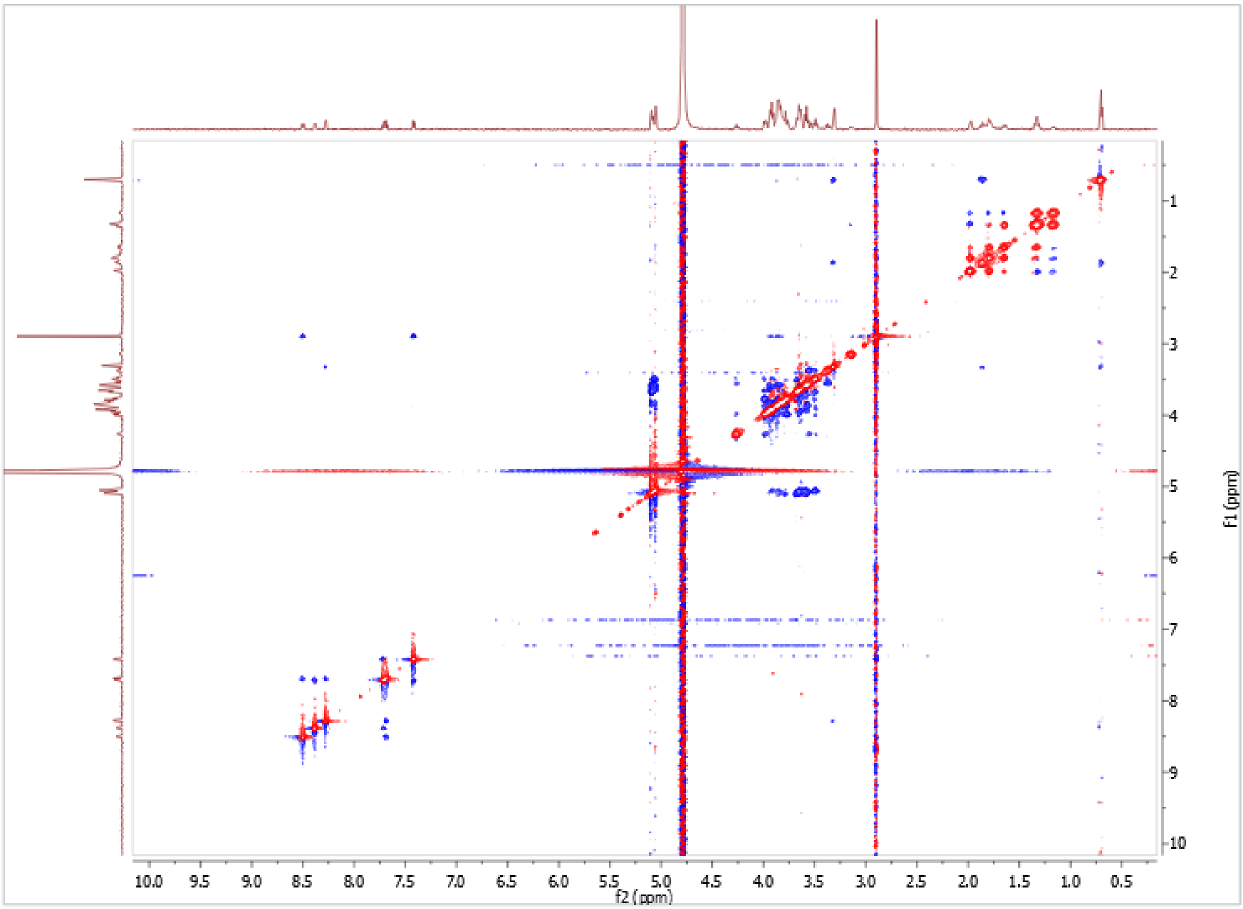

Figure S63. 2D ROESY spectrum of *D*,*L*-Dns-Val/PIP-β-CD (600 MHz, 298 K, D_2O).

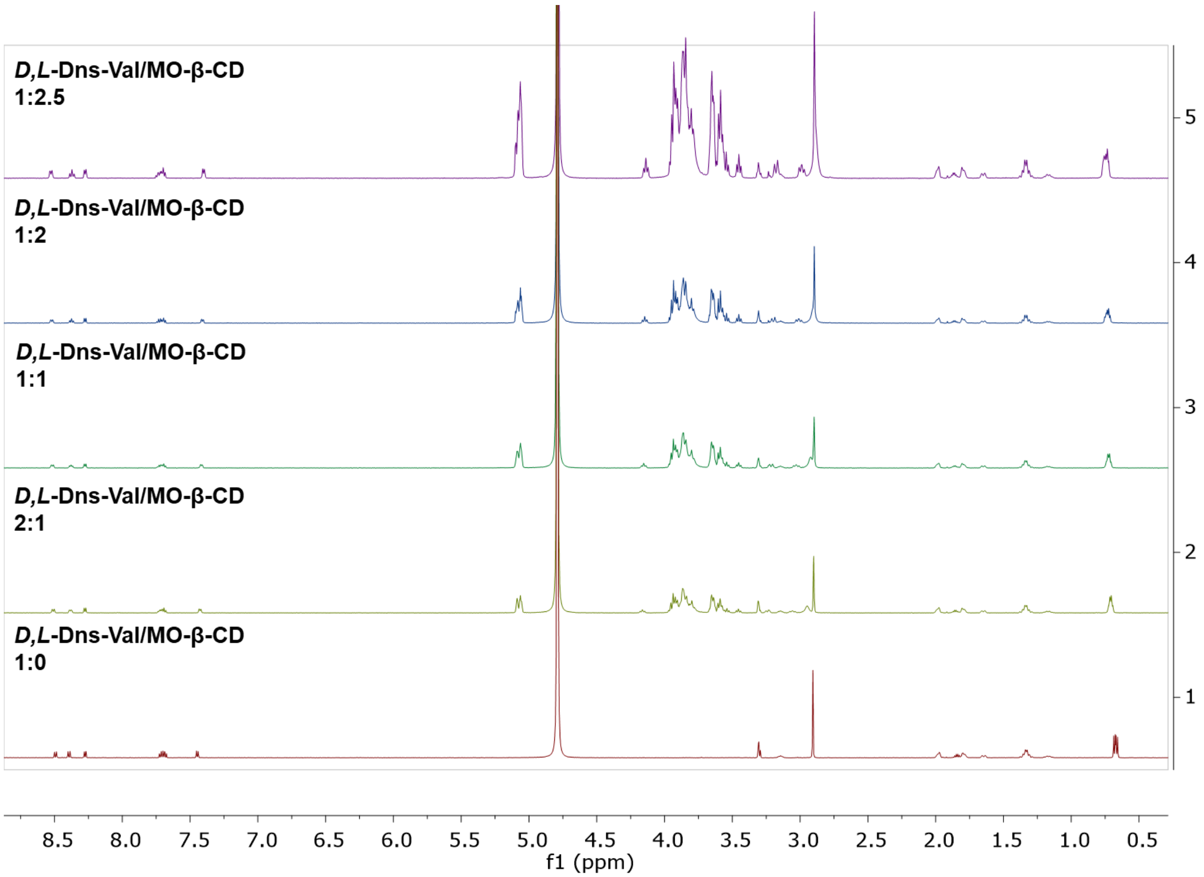

Figure S64. Stacked ^{1}H NMR spectra of *D,L*-Dns-Val/MO-β-CD system in 20 mM NaH_2PO_4 buffer at pH* 6.0 with increasing MO-β-CD concentration (from bottom to top) (600 MHz, 298 K).

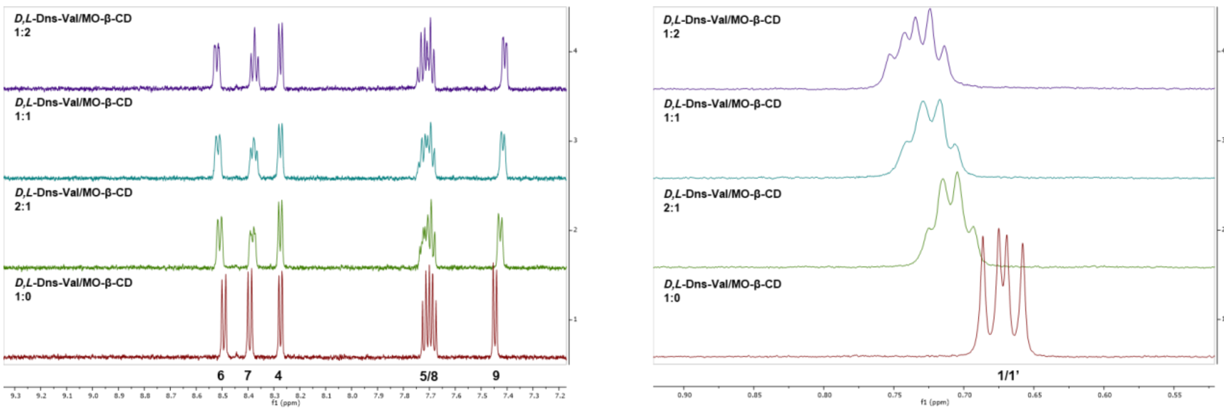

Figure S65. Stacked partial ^{1}H NMR spectra of *D,L*-Dns-Val/MO-β-CD system in 20 mM NaH_2PO_4 buffer at pH* 6.0 with increasing MO-β-CD concentration (from bottom to top) (600 MHz, 298 K).

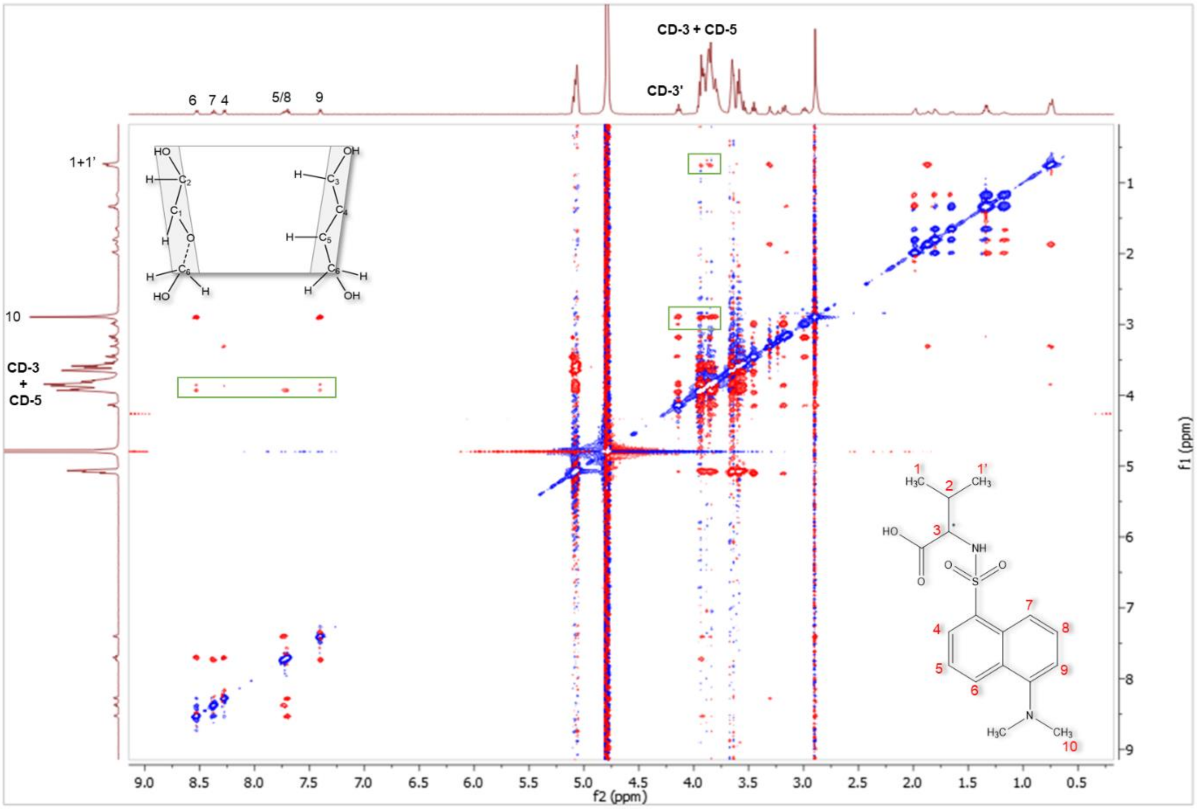

Figure S66. 2D ROESY spectrum of *D*,*L*-Dns-Val/MO-β-CD system, with intermolecular correlations emphasized and structure of the compounds.

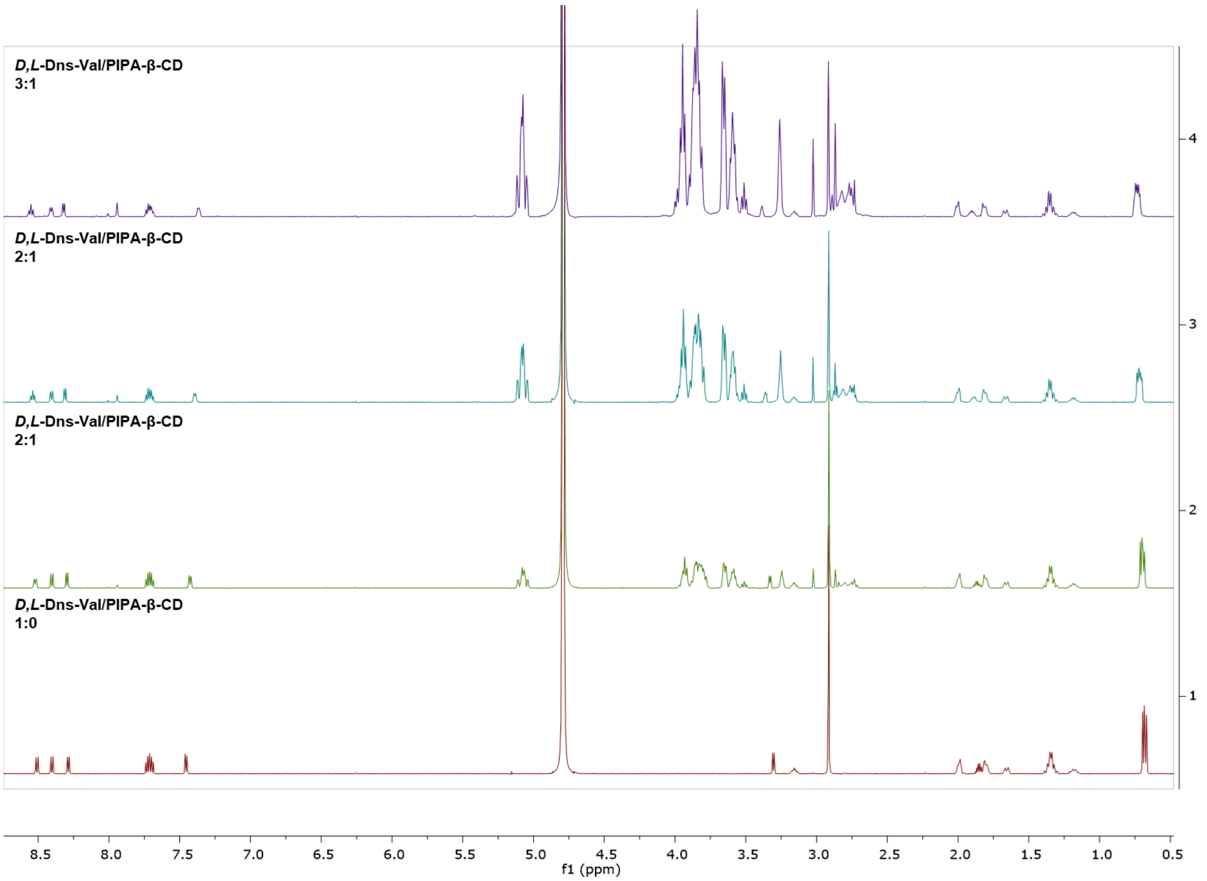

Figure S67. Stacked ^{1}H NMR spectra of *D*,*L*-Dns-Val/PIPA-β-CD system in 20 mM NaH_2PO_4 buffer at pH* 6.0 with increasing PIPA-β-CD concentration (from bottom to top) (600 MHz, 298 K).

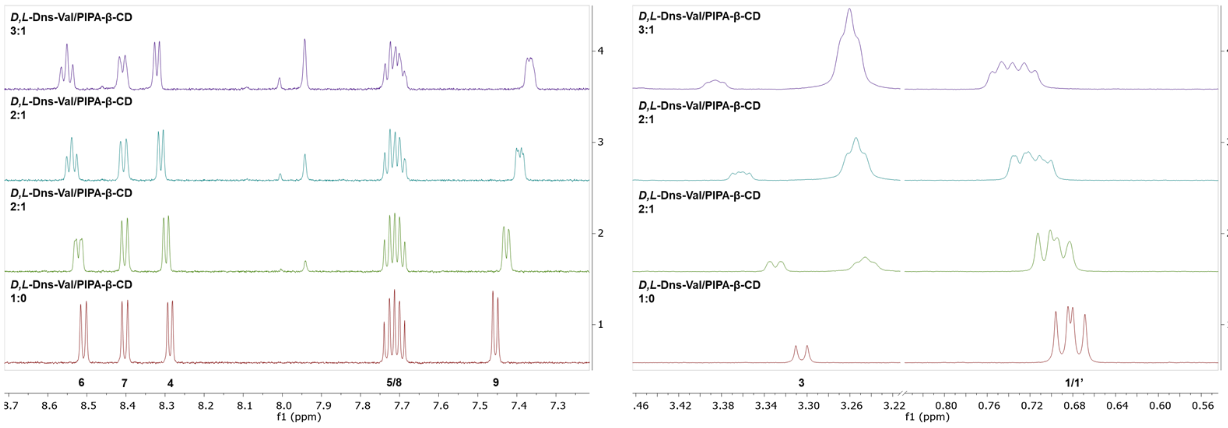

Figure S68. Stacked partial ^{1}H NMR spectra of *D*,*L*-Dns-Val/PIPA-β-CD system in 20 mM NaH_2PO_4 buffer at pH* 6.0 with increasing PIPA-β-CD concentration (from bottom to top) (600 MHz, 298 K).

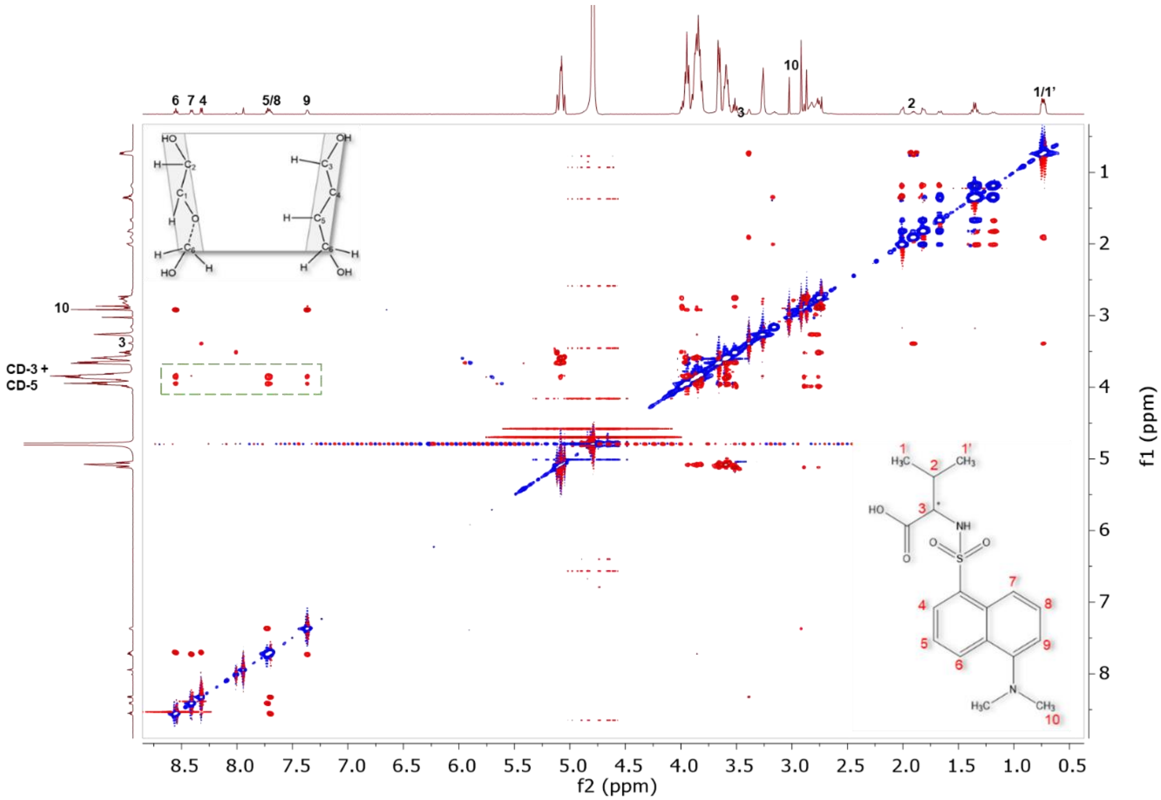

Figure S69. 2D ROESY spectrum of *D*,*L*-Dns-Val/PIPA-β-CD system, with intermolecular correlations emphasized and structure of the compounds (600 MHz, 298 K, D_2O).

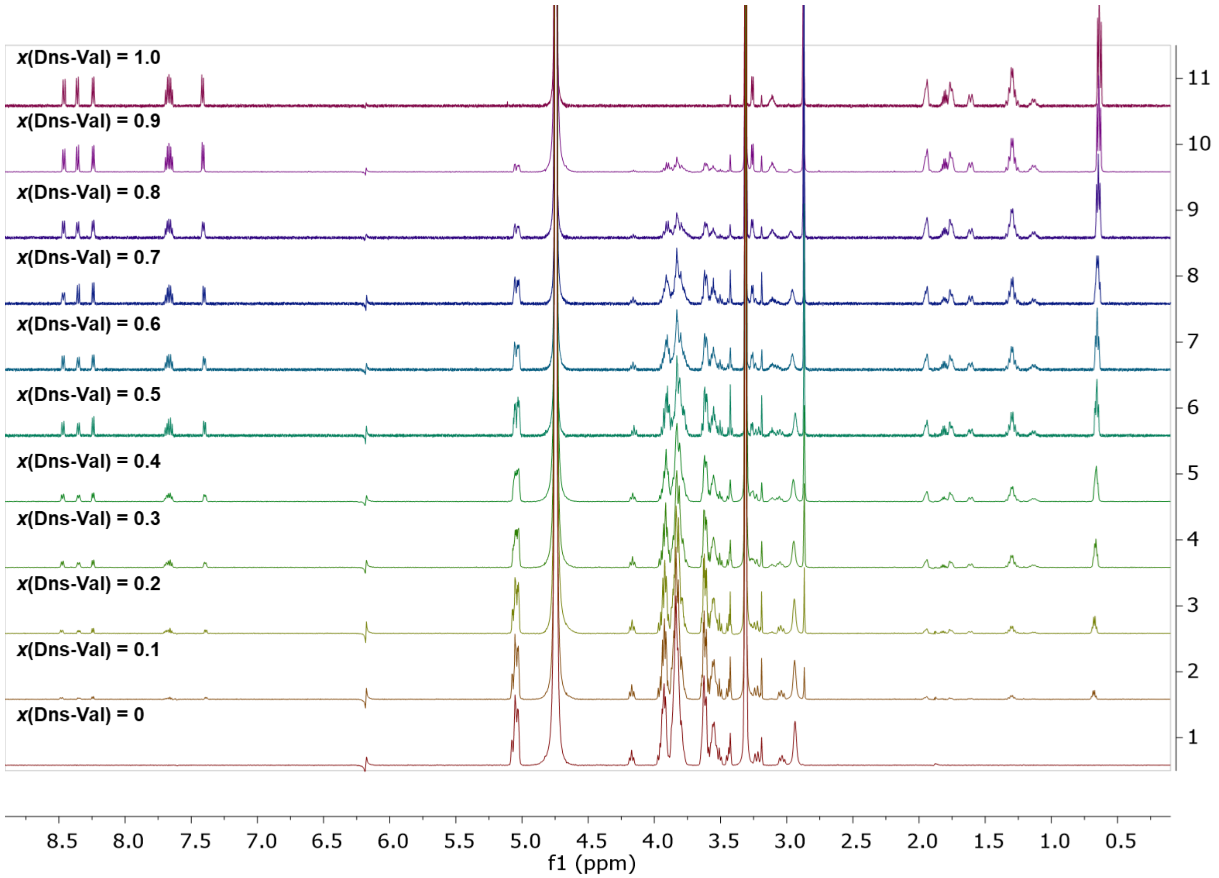

Figure S70. Stacked series of ^{1}H NMR spectra of Dns-Val/MO-β-CD system with increasing molar fraction of MO-β-CD from top to bottom

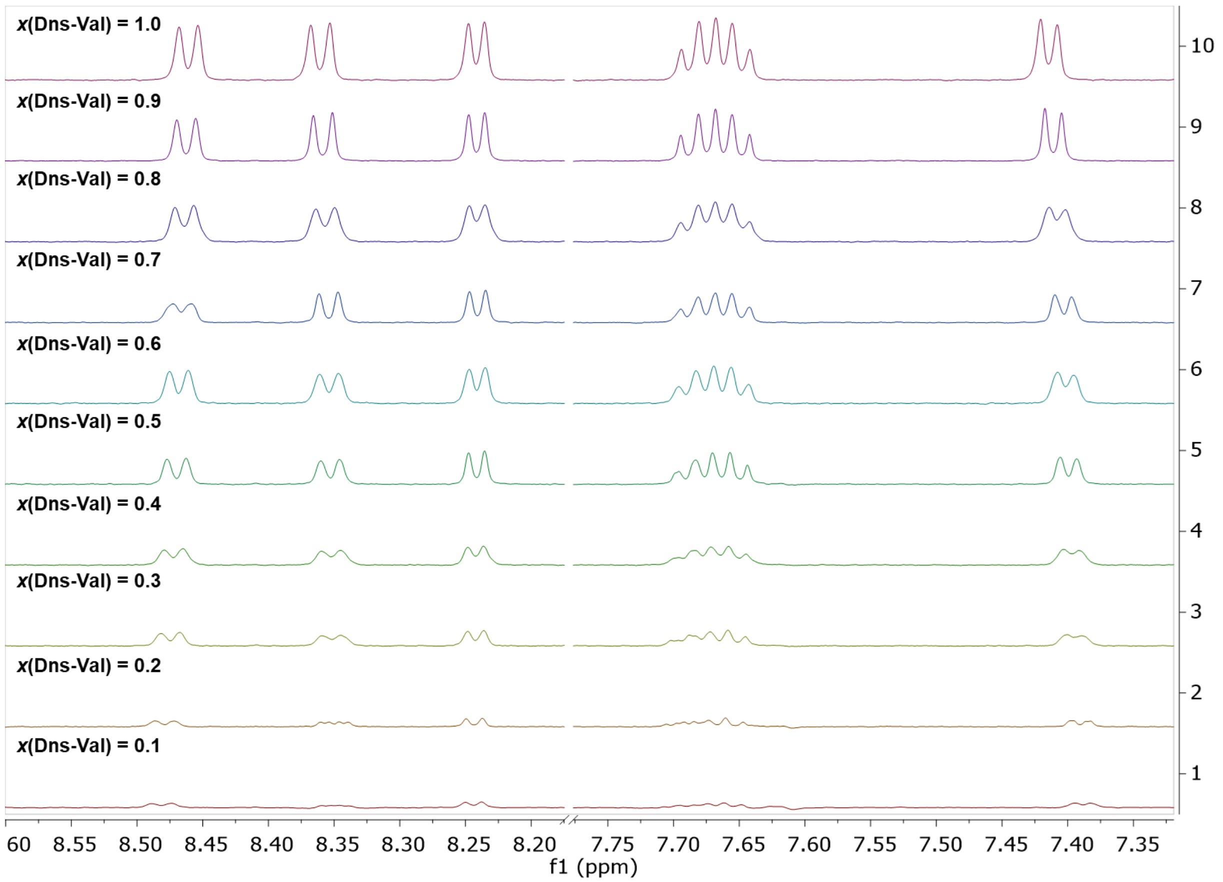

Figure S71. Chemical shift changes of the aromatic protons of Dns-Val upon changing the mole fraction of Dns-Val in the presence of MO-β-CD

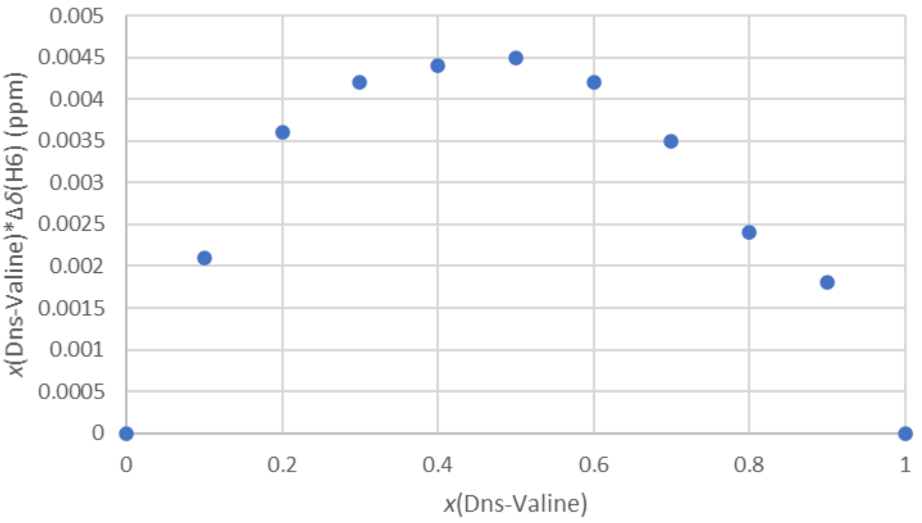

Figure S72. Job's plot derived from the chemical shift changes of H6 proton of Dns-Val in presence of MO-β-CD
